# *Lgr4* Regulates Oviductal Epithelial Secretion Through the WNT Signaling Pathway

**DOI:** 10.3389/fcell.2021.666303

**Published:** 2021-09-24

**Authors:** Xue Tan, Lingling Zhang, Tianqi Li, Jianmin Zhan, Kun Qiao, Haili Wu, Shenfei Sun, Meina Huang, Fangxi Zhang, Meixing Zhang, Changwei Li, Runsheng Li, Hongjie Pan

**Affiliations:** ^1^National Health Commission (NHC) Key Laboratory of Reproduction Regulation, Shanghai Institute for Biomedical and Pharmaceutical Technologies, School of Pharmacy, Fudan University, Shanghai, China; ^2^Center for Reproductive Medicine, Tenth People’s Hospital of Tongji University, Shanghai, China; ^3^Shanghai Endangered Species Conservation and Research Centre, Shanghai Zoo, Shanghai, China; ^4^State Key Laboratory of Genetic Engineering, School of Life Sciences, Fudan University, Shanghai, China; ^5^Shanghai Key Laboratory for Prevention and Treatment of Bone and Joint Diseases With Integrated Chinese-Western Medicine, Ruijin Hospital, Shanghai Institute of Traumatology and Orthopedics, Shanghai Jiao Tong University School of Medicine, Shanghai, China

**Keywords:** secretory cell, oviduct, LGR4, WNT/CTNNB1, NR5A2

## Abstract

The WNT signaling pathway plays a crucial role in oviduct/fallopian development. However, the specific physiological processes regulated by the WNT pathway in the fallopian/oviduct function remain obscure. Benefiting from the *Lgr4* knockout mouse model, we report the regulation of oviduct epithelial secretion by LGR4. Specifically, the loss of *Lgr4* altered the mouse oviduct size and weight, severely reduced the number of oviductal epithelial cells, and ultimately impaired the epithelial secretion. These alterations were mediated by a failure of CTNNB1 protein accumulation in the oviductal epithelial cytoplasm, by the modulation of WNT pathways, and subsequently by a profound change of the gene expression profile of epithelial cells. In addition, selective activation of the WNT pathway triggered the expression of steroidogenic genes, like *Cyp11a1* and *3*β-*Hsd1*, through the activation of the transcriptional factor NR5A2 in an oviduct primary cell culture system. As demonstrated, the LGR4 protein modulates a WNT-NR5A2 signaling cascade facilitating epithelial secretory cell maturation and steroidogenesis to safeguard oviduct development and function in mice.

## Introduction

The oviduct, also known as the fallopian tube in humans, is a part of the female reproductive tract. For decades, a growing body of evidence indicates the oviduct serves not merely as a pipeline allowing the transit of gametes and embryo but also provides structural, environmental, and nutritional support for the conception (Menezo et al., 2015; Li and Winuthayanon, 2017; Lee et al., 2018; No et al., 2018; Wang and Larina, 2018). The physiological oviduct functions include the pickup of oocytes by the leaf-like folds in the infundibulum, the maturation of the ovum, and favoring the oocyte fertilization in the ampulla and early embryo development in the ampulla and isthmus (Li et al., 2019). Impairments of fallopian tube functions lead to frequent failures to conceive (Shaw and Horne, 2012; Hendriks et al., 2020). However, altogether the oviduct is less well understood for its contribution to reproduction compared with other female reproductive organs, like the ovary and uterus. This is because the assisted reproductive technologies (ARTs), such as *in vitro* fertilization (IVF) and intracytoplasmic sperm injection (ICSI), bypass the human fallopian tubes entirely.

A member of the WNT/CTNNB1 signaling pathway, the Wnt1, has been described for the first time about 35 years ago (Nusse and Varmus, 1982). Over the years the scientists recognized that this signaling pathway is one of the most fundamental pathways throughout the animal kingdom (Nusse and Clevers, 2017). The canonical WNT signaling pathway is initiated by the specific binding of WNTs to frizzled class receptors (FZDs), followed by the degradation of glycogen synthase kinase 3 beta (GSK3β) and the activation of CTNNB1 (β-catenin) in the cytoplasm. Consequently, accumulated CTNNB1 becomes able to translocate into the nucleus, where it interacts appropriately with a variety of transcription factors to modulate gene expression. Various studies revealed precisely that the canonical WNT plays a crucial role in ontogenesis. WNT signaling regulates the mouse Müllerian duct development, which is a necessary precursor of the female reproductive tract (Deutscher and Hung-Chang Yao, 2007; Prunskaite-Hyyrylainen et al., 2016). Moreover, the elongation and coiling of the oviduct are controlled by Wnt7a (Parr and McMahon, 1998). It also has been shown that an enhanced CTNNB1 is positively associated with a higher frequency of tubal pregnancies which accounts for 98% of ectopic pregnancies in humans (Li et al., 2013; Wang et al., 2019). Ciliary and secretory cells are the two most abundant cell types in the oviduct/fallopian epithelium, which are structurally pseudostratified on the stroma. However, selective ablation of CTNNB1 from the oviduct ciliated cells does not affect embryo transport, which might be because of compensatory mechanisms *via* intact CTNNB1 expression in adjacent epithelial secretory cells (Li et al., 2017). Further, an *in vivo* cell lineage tracing work reveals that the canonical WNT pathway is required for the homeostasis of the ratio of ciliated and secretory epithelial cells (Ghosh et al., 2017). These studies imply the secretory epithelial cells have vital physiological roles; however, the specific action of CTNNB1 in secretory cells remains to be deciphered.

A key regulator of WNT/CTNNB1 signaling is the leucine-rich repeat containing G protein-coupled receptors (LGRs). LGR4, LGR5, and LGR6, three closely related LGRs, function as the receptors for R-spondins, which modify precisely the WNT effect. LGR5 is identified as the mark of stem/progenitor cells in oviductal epithelia in mice (Ng et al., 2014). In addition, it has been shown that LGR6 is required for human fallopian organoid development (Kessler et al., 2015). We and others demonstrated that *Lgr4* expresses in a variety of tissues and plays a significant role in amplifying the WNT/CTNNB1 signal in multiple tissues and organs, like hair follicles (Zak et al., 2016), ovaries (Pan et al., 2014), kidneys (Vidal et al., 2020), intestine (Liu et al., 2013), male reproductive tract (Qian et al., 2013), uterus (Sone et al., 2013), and many others (Wang et al., 2013; Han et al., 2014; Planas-Paz et al., 2016). However, to date, the role of LGR4 in the oviduct/fallopian tube is still obscure.

Here, we hypothesize that besides homeostasis, the canonical WNT pathway regulates the physiological function of secretory cells in mouse oviducts and that this regulation requires the precise action of LGR4.

## Materials and Methods

### Ethics Statement

All mice were kept under controlled photoperiod conditions (lights on 07:00–19:00 h) and were supplied with food and sterilized H_2_O *ad libitum*. Experiments were conformed to the regulations drafted by the Association for Assessment and Accreditation of Laboratory Animal Care in Shanghai. The ethics committee approval was obtained from the Institutional Ethics Committee of Shanghai Institute of Planned Parenthood Research Center (now Shanghai Institute for Biomedical and Pharmaceutical Technologies) for the commencement of the study.

### Mice

The C57BL/6 *Lgr4* gene-trap mouse strain was generated as previously described (Weng et al., 2008). Genomic DNA was extracted from tail biopsies and analyzed using the TaKaRa Taq^TM^ Hot version (Cat# R007A). Primers for genotyping are listed in Supplementary Table 1. The 23- to 25-day-old females were sacrificed to perform a series of *in vivo* analyses.

### Hematoxylin and Eosin Staining and Immunohistochemistry

Oviducts were collected and fixed in Bouin’s solution for hematoxylin and eosin (H&E) staining and in 4% paraformaldehyde (PFA) for immunohistochemistry (IHC). For IHC assay, sections (4–5 μm) were deparaffinized in xylene and rehydrated in gradient alcohol concentrations. After antigen retrieval, the sections were blocked by 5% bovine serum albumin (BSA). The sections were then incubated with primary antibodies overnight at 4°C. The next day, the sections were incubated with secondary antibodies for 20 min and then developed with 3,3′-diaminobenzidine and counterstained with hematoxylin. Antibodies were diluted as follows: OVGP1 at 1:100 (Santa Cruz, sc-377267), PAX8 at 1:100 (Proteintech Cat# 60145-4-lg), CTNNB1 at 1:100 (BD Transduction Laboratories^TM^, Cat# 610153), NR5A1 at 1:200 (Abcam Cat# ab217317), and NR5A2 at 1:500 (Abcam Cat# ab189876). Samples from at least three animals were evaluated to ensure reproducibility of the results.

### Alcian Blue Staining

Deparaffinized and hydrated slides were incubated in 3% acetic acid for 3 min, followed by staining in 1% Alcian blue solution (pH 2.5) for 30–60 min. The sections were washed in running tap water for 2 min and rinsed in diH_2_O. After dehydration, tissue sections were mounted and observed under a microscope.

### Periodic Acid–Schiff Staining

Deparaffinized and hydrated slides were incubated in 0.5% periodic acid solution for 5 min. After rinsing in three changes of distilled water, sections were placed in Schiff’s reagent for 15 min, followed by washing in tap water for 5 min. After dehydration, the sections were mounted and observed under a microscope.

### β-Gal Staining

The frozen sections were fixed in 0.2% glutaraldehyde in phosphate-buffered saline (PBS) for 15 min on ice then washed in PBS for 10 min. Then the sections were immersed in 1 mg/ml X-gal staining solution overnight at 37°C in the dark. A post-fixation in 4% PFA for 10 min was required. After rinsing in PBS, the sections were counterstained with nuclear fast red then rinsed in distilled water for 2 min. After dehydration, sections were mounted and observed under a microscope.

### Mouse Oviduct Epithelial Cell Isolation, Cell Culture, and ELISA Assay

Briefly, the mouse oviduct was surgically isolated and placed into an enzymatic media [Dulbecco’s minimum essential medium DMEM/F12 supplemented with 0.25% collagenase type IV (Invitrogen, 17104019), and 10 U/ml DNase I (Sigma, 10104159001)] for 15 min at 37°C. The softened tissue was then washed two times with PBS and transferred into a dissection medium [PBS with 0.5% penicillin/streptomycin and 1% fetal bovine serum (FBS)]. The oviduct tissue was then mechanically dissected using micro-scissors. The oviductal cell suspension was diluted with an equal volume of medium DMEM/F12 supplemented with 5% fetal bovine and centrifuged twice at 300 × *g* for 5 min at 4°C. The supernatant was removed, and the pellet of oviductal cells was re-suspended in culture media (DMEM/F12 supplemented with 10% FBS, 1% insulin, transferrin, and selenium solution) and seeded in a 24-well plate at 37°C and 5% CO_2_ in a humidified incubator. The culture medium was changed every 2 days. Once the adhered cells reached 70% confluence, we conducted the co-culture assay or E2 stimulated ELSA assays (OVGP1 kit: MyBioSource, Cat# abx576628, San Diego, CA, United States; CXCL8/IL-8 kit: R&D, Cat# D8000C). Before the E2 stimulation, cells were cultured in a medium without phenol red and FBS for at least 24 h.

### Collection of Mouse Germinal Vesicle Oocytes and Co-culture

On the day of oocyte collection, the medium of the epithelial cell culture was changed from the DMEM/F12 to M2 medium (Sigma, M7167). Germinal vesicle stage oocytes (GV oocytes) were collected from 6-week-old C57BL/6 female mice injected with 5 IU of pregnant mare’s serum gonadotropin (PMSG; Sigma) 40 h before. Then the cumulus–oocyte complexes were obtained by puncturing the antral follicles, denuded by repeat pipetting using a stripper in 0.3 mg/ml hyaluronidase (Sigma H4272), and classified by GV stage. GV oocytes were co-cultured with epithelial cells in M2 media. Oocytes were observed at 12 and 24 h for oocyte maturation. Grading of oocytes was performed by microscopic examination. Progression to the metaphase II (MII) stage was identified by extrusion of the first polar body into the perivitelline space, which was observed under a microscope.

### Chromatin Immunoprecipitation Assay

Mouse oviductal epithelial cells (MOECs) were treated with or without proteins WNT4, RSPONDIN2, or compound CT99021 overnight, and chromatin immunoprecipitation (ChIP) analysis was performed as previously described (Wu et al., 2007). DNA was precipitated with a polyclonal NR5A2 (Abcam Cat# ab189876) antibody or mouse IgG as a negative control. Precipitated DNA was amplified with the primers designed to the *Cyp11a1* and *Hsd3b1* promoter regions. See the detailed information of the primers in Supplementary Table 1. PCR products were confirmed by sequencing.

### RNA-Seq Data Analysis

#### RNA-Seq Library Preparation

For RNA sequencing, four *Lgr4* knockout (KO) and control mice were sacrificed, and the oviduct tissues were homogenized in TRIzol reagent and followed by RNA isolation. Isolation of mRNA was performed using the NEBNext PolyA mRNA Magnetic Isolation Module (New England Biolabs, Ipswich, MA, United States). The mRNA was then used for RNA-seq library preparation with the NEB Next Ultra Directional RNA Library Prep Kit for Illumina (New England Biolabs, Ipswich, MA, United States). The library was subjected to Illumina sequencing with paired-end 2 × 150 as the sequencing mode.

#### Quality Control and Alignment of Sequencing Data

Raw reads were filtered to obtain high-quality clean reads by removing sequencing adapters, short reads (length <35 bp), and low-quality reads using Cutadapt v1.18 and Trimmomatic v0.35. Then FastQC (with default parameters) is used to ensure high reads quality. The clean reads were mapped to the mm10 genome by HISAT2 v2.1.0.

### Analysis of Differential Expression

Gene expression levels were estimated using fragments per kilobase of exon per million fragments mapped (FPKM) by StringTie v1.3.4d (Pertea et al., 2015). EdgeR v3.24.2, a R package, was used to measure differential gene expression (Robinson et al., 2010). The false discovery rate (FDR) control method was used to calculate the adjusted *p*-values in multiple testing to evaluate the significance of the differences. Genes with an adjusted *p*-value < 0.001, |log2FC| ≥ 2, and FPKM > 1 were used for subsequent analysis.

### Functional Enrichment Analysis

A gene annotation file was retrieved from ensembl genome browser 96 database.^1^ To annotate genes with Gene Ontology (GO), clusterProfiler v3.4.4 (R package) was used.

The raw next-generation sequencing (NGS) data were deposited to the NCBI SRA database under project number (PRJNA691984). Reverse transcription polymerase chain reaction (RT-PCR) was performed to verify the mRNA-seq results using SYBR Premier EX Taq (TaKaRa). Genes were amplified with the indicated primers (Supplementary Table 1). Relative levels of mRNAs were calculated using MX3500pro software and normalized to the levels of endogenous actin in the same samples.

### Statistical Analysis

Statistical data were analyzed with GraphPad Prism version 5. The statistical data of oviduct weight, numbers of specific stained secretory cells in the oviduct epithelium, and percentages of different stages of oocyte and RT-PCRs were presented as means ± SEM. Analysis of variance or Student’s *t*-test was used for statistical comparison to determine significance. Statistical significance was set as NS, *p* > 0.05; ^∗^*p* ≤ 0.05; ^∗∗^*p* ≤ 0.01; and ^∗∗∗^*p* ≤ 0.001. All presented results were from at least three independent experiments. The investigators were blinded to the group allocation during the experiment and when assessing the outcome.

## Results

### The Expression of *Lgr4* in Mouse Oviduct

**FIGURE 1:**
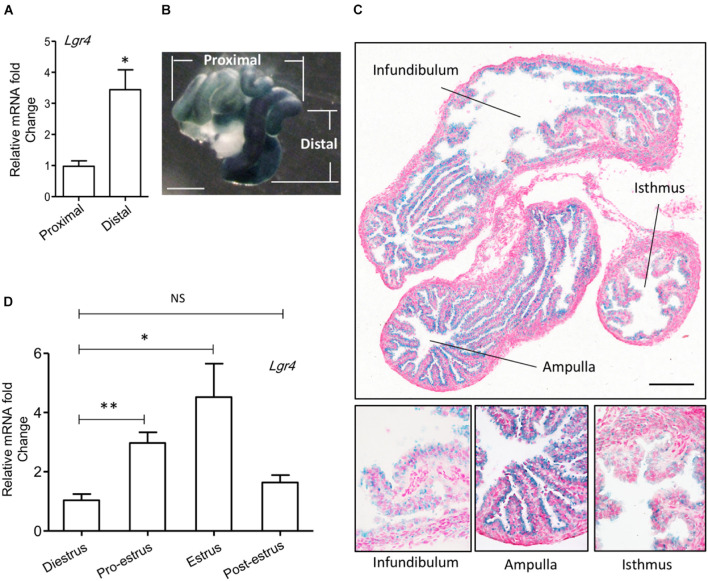
Expression of *Lgr4* in oviducts of mice. **(A)** The relative expression of *Lgr4* mRNA in the specific regions of the oviduct of the WT mice. **(B)** A whole mount *LacZ* staining represented the spatial expression pattern of *Lgr4* in a heterozygous mouse oviduct. **(C)** A LacZ staining of a cryo-section of an *Lgr4* heterozygous mouse oviduct. **(D)** The relative expression of *Lgr4* mRNA in the mouse oviduct at different stages of the estrus cycle. At least three independent experiments were carried out. Data are presented as mean ± SEM of three mice. ^∗^*p* ≤ 0.05; ^∗∗^*p* ≤ 0.01. Scale bar, 1,000 μm in **(B)**; 200 μm in **(C)**.

To study the role of *Lgr4* in the oviduct, we analyzed its gene expression pattern. The mRNA level of *Lgr4* was significantly enhanced by 3.52-fold in the distal region compared with the proximal region of the mouse oviduct (Figure 1A). As LGR4, LGR5, and LGR6 are the three most closely related leucine-rich repeat family of G-protein-coupled receptors (de Lau et al., 2012), we also determined the mRNA levels of *Lgr5* and *Lgr6*. Unlike *Lgr4*, the expressions of *Lgr5* and *Lgr6* showed no differences between the distal and proximal regions in the mouse oviduct (Supplementary Figures 1A,B). To further identify the distribution of LGR4 in the oviduct, we took advantage of the β-gal reporter gene, which was inserted into the first intron of the *Lgr4* genomic locus to track endogenous *Lgr4* mRNA expression (Weng et al., 2008). The β-gal signal was enhanced strongly in the distal region (dark blue) compared with the slightly stained proximal region (Figure 1B). From the tissue morphology, the β-gal positive cells were oviductal epithelial cells. Although a positive signal could be detected in the epithelial layer of all three regions (infundibulum, ampulla, and isthmus), it was clear that the signal in the ampulla was the strongest compared with that in the other two regions (infundibulum and isthmus) (Figure 1C). Under specific hormonal controls during different stages of the estrus cycle, the oviduct produces different levels of fluid and macromolecules that optimize the microenvironment for gamete maturation. Therefore, we examined mRNA levels of *Lgr4* expression. RT-PCR assay revealed that the *Lgr4* expression was enhanced by more than 2.85-fold at the pro-estrus stage and then reached its peak level at 4.35-fold at the estrus stage compared with the di-estrus stage. This result suggested the expression of *Lgr4* fluctuated dynamically during the estrus cycle (Figure 1D). The data showed that *Lgr4* was present in the oviductal epithelium and with a significantly enhanced expression level at the distal region, especially in the ampulla, suggesting a physiological function of *Lgr4* in this specific region within the estrus cycle.

### Knockout of *Lgr4* Led to Histological Deficiencies in the Oviduct

**FIGURE 2:**
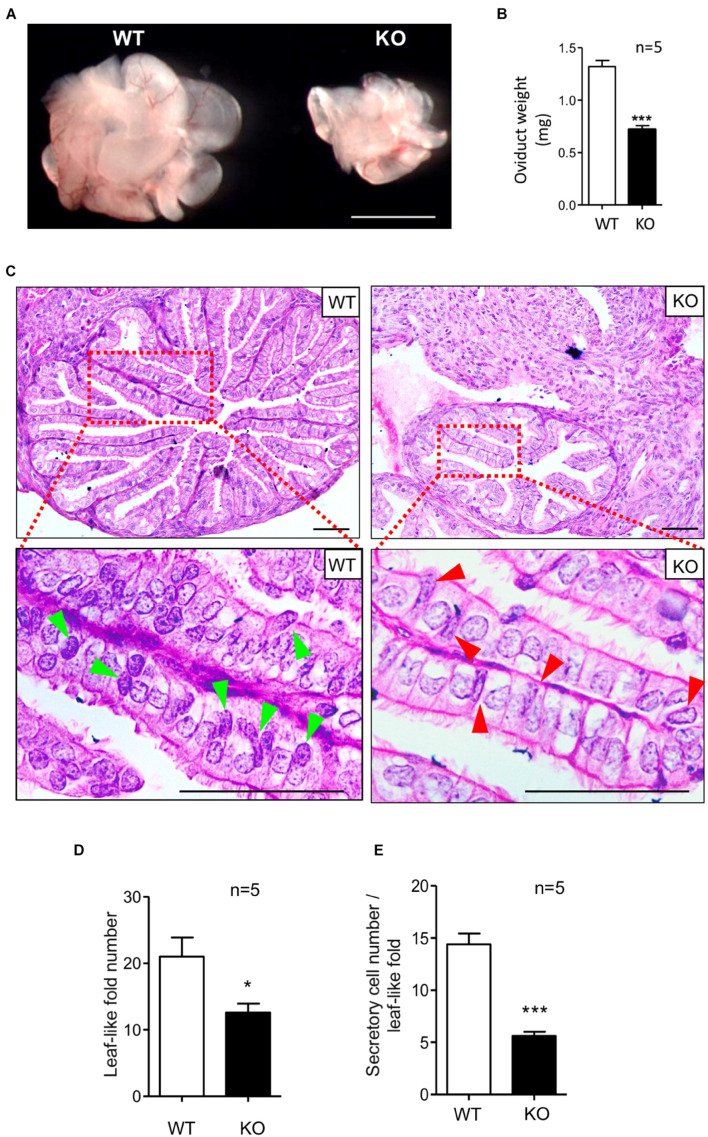
Defects of oviducts in response to *Lgr4* knockout. **(A)** Morphology of oviducts. **(B)** The mean weights of the *Lgr4* knockout and WT mouse adult oviducts. **(C)** Histology of the WT and *Lgr4* KO oviducts was determined by hematoxylin and eosin staining. The red boxed areas are shown at higher magnification beneath. **(D)** The number of the leaf-like folds. **(E)** The number of the secretory cells per leaf-like folds. *p ≤ 0.05; ***p ≤ 0.001. Scale bar, 1,000 μm in **(A)**; 50 μm in **(C)**.

To determine the physiological role of the LGR4 protein in the murine oviduct, we compared the mutant and WT oviduct. The oviducts of KO female mice were significantly reduced in size (Figure 2A). In addition, the mean weight of isolated oviducts was 0.72 mg in the mutant mice, which was significantly lower than that in the WT group (1.32 mg) (Figure 2B). H&E staining was performed to determine the histological changes between the *Lgr4* KO and WT oviducts. The cross-sectional area of the *Lgr4* KO ampulla region was reduced in comparison with the WT control (Figure 2C, upper panel). The average number of the leaf-like folds in the WT ampulla was 21.00, while it was 12.60 in the mutant ampulla (Figure 2D). This result suggested that the loss of *Lgr4* led to a reduced oviductal leaf-like folds. Furthermore, the columnar secretory cells with spot-like granules but no cilia were stained in dark blue in the WT (arrowed in bright green), while such type cells with less spot-like granules stained were reduced in the *Lgr4*-deficient oviduct (arrowed in red) (Figure 2C, lower panel). In the WT leaf-like fold, the average number of these secretory cells was 14.40, while in the mutant leaf-like fold, the number was reduced significantly to 5.60 (*p* < 0.001) (Figure 2E). As an ovarian dysfunction resulted from *Lgr4* ablation (Pan et al., 2014), we removed the ovaries from both the WT and KO mice, and the ovariectomized female mice were then treated with daily injections of 100 ng per mouse of 17-β-estradiol or 1 mg per mouse of progesterone dissolved in 100 μl of sesame oil according to a previous report (Xiao et al., 2002). Histological defects with reduced numbers of leaf-like folds and secretory cells were still observed in the mutant ampulla (Supplementary Figures 2A–C). Together, these results suggested that the *Lgr4* gene has an important role in the secretory cells in mouse oviduct.

### Ablation of *Lgr4* Resulted in Secretory Deficiency of the Oviduct

**FIGURE 3:**
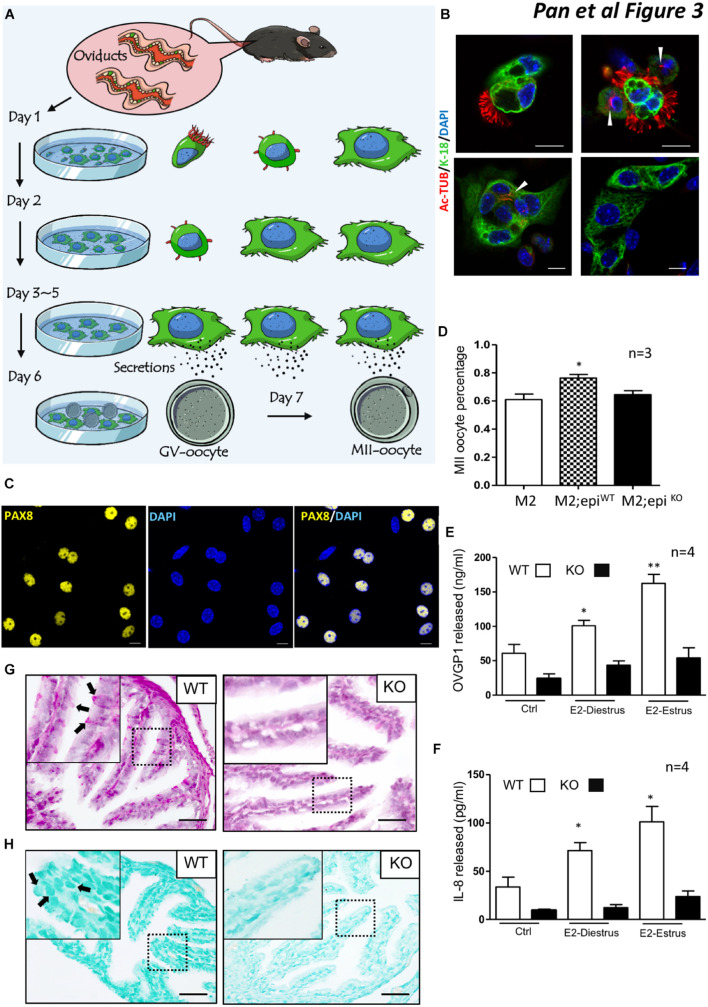
Secretion deficiencies in the oviducts of *Lgr4* KO mice. **(A)** A schematic showing the procedure for mouse oviduct epithelial cell–oocyte co-culture system. **(B)** Immunofluorescence analysis of cytokeratin 18 (KRT-18) and alpha-tubulin (Ac-Tub) to the mouse oviduct epithelial cells. **(C)** Immunofluorescence analysis of PAX8. **(D)** The average percentage of the MII oocytes in response to different treatments. **(E)** The released protein level of OVGP1. **(F)** The released protein level of IL-8. **(G)** The periodic acid–Schiff (PAS) staining between the WT and *Lgr4* knockout oviduct. **(H)** The Alcian blue staining between the WT and *Lgr4* knockout oviduct. ^∗^*p* ≤ 0.05; ^∗∗^*p* ≤ 0.01. Scale bar, 10 μm in **(B,C)**, 25 μm in **(G,H)**. At least three independent experiments were carried out.

One fundamental role of the oviduct is to secrete factors to support and promote oocyte maturation. We used a two-type cell co-culture assay as outlined in the methods to detect whether ablation of *Lgr4* led to a deficiency in oviduct secretion (Figure 3A). Before the examination, we measured the cytokeratin (cytokeratin-18) and alpha-tubulin (Ac-Tub) levels in MOEC cultures. One day after seeding, free-floating columnar MOECs with apparent cilia on the external surface (left) and adhered MOECs with reduced cilia (right) were observed (Figure 3B, upper panel). Two days later, the main cell type in the well was the adhered MOECs with (left) or without reduced cilia (right) (Figure 3B, lower panel). Three days later, almost all the adherent MOECs lost their cilia. PAX8 is reported to be a typical secretory cell marker in the oviduct (Ghosh et al., 2017; Ito et al., 2020). Most of the adhered cells at this time expressed PAX8 (Figure 3C). The data suggested that the cultured oviductal epithelial cells retained their secretory cell characteristic without cilial cells. As the co-culture system is widely used to promote the maturation of oocytes (Lee et al., 2011, 2017; Dadashpour Davachi et al., 2016), it can also show the secretory function of the oviduct epithelial cell in promoting oocyte maturation. When both the WT and KO adhered MOECs reached 70% confluence, we co-cultured the MOECs with the mouse GV oocytes for 12–24 h. The percentage of the GV oocytes that developed to the MII stage (MII oocyte) in the MII medium only was 61.00%. When the GV oocytes were co-cultured with WT mouse oviduct epithelial cells, the percentage enhanced significantly to 76.26%. However, when the GV oocytes were co-cultured with the *Lgr4* mutant MOECs, the percentage upregulated slightly but not significantly to 64.57% compared with the MII medium only group (Figure 3D). The oviduct epithelium is covered with a mucosal surface containing mucins (MUCs) at its apical site, which provides a physical barrier against invading pathogens. After pathogen recognition through Toll-like receptors, epithelial cells release several chemokines, e.g., interleukin 8 (IL-8), to attract the immune system (Lagow et al., 1999; Zerbe et al., 2003; Chaturvedi et al., 2008; Kowsar et al., 2013). Oviduct-specific glycoprotein 1 (OVGP1), also named MUC9, is one of the most important glycoproteins secreted directly by the oviduct epithelial cells (Aviles et al., 2010; Saint-Dizier et al., 2014; Algarra et al., 2016; Lamy et al., 2018; Zhao and Kan, 2019). Therefore, we examined the protein levels of released OVGP1 and IL-8 in the WT and KO cultured MOEC supernatants. The oviduct is highly sensitive to the fluctuating ovarian hormone levels, so we supplemented the cultured MOECs with 17-β-estradiol (E2) mimicking the estrus effect, as the E2 influences the secretion of MOECs positively (Chen et al., 2013, 2018). The average OVGP1 level of the WT MOEC supernatant was 60.88 ng/ml, while this protein level was induced significantly to 100.89 ng/ml at the E2-diestrus stage (E2 = 10.00 pg/ml). The level was further enhanced to 162.45 ng/ml in the WT MOEC supernatant at the E2-estrus stage (E2 = 50.00 pg/ml). However, the average OVGP1 levels of the KO MOEC supernatant were 24.27, 43.51, and 54.30 pg/ml at each specific stage without any significant changes (Figure 3E). Similarly, the average IL-8 levels were 33.70, 71.45, and 101.22 ng/ml specifically, which were enhanced significantly under the stimulation of different concentrations of E2 in the WT MOEC supernatant. The average IL-8 levels of the KO MOEC supernatant were 9.65, 12.32, and 23.82 pg/ml at each specific stage without significant changes (Figure 3F).

To further examine the role of *Lgr4* in the secretion function of the mouse oviduct *in vivo*, we performed a set of histochemistry assays on the WT and *Lgr4* KO oviduct in the estrus stage. The periodic acid–Schiff (PAS) staining was utilized to detect the presence of carbohydrates and carbohydrate compounds such as glycogen in the oviduct. The specific staining was observed in the WT group (arrowed in black), while in the *Lgr4*-deficient group only a low number of the specific stainings were detected (Figure 3G). In the *Lgr4* KO group, the average number of the positive PAS staining signal was 2.00 per leaf-like fold, which was significantly lower than the wild type counterpart, 10.16 (Supplementary Figure 3A). A similar staining pattern was also found in the Alcian blue assay which was used to detect the acid mucosubstances and MUCs. Specifically, apparent signals were observed in the WT group (arrowed in black), while in the deficient group, the signals were reduced severely (Figure 3H). In the *Lgr4* KO group, the average number of the positive Alcian blue staining signal was 2.33 per leaf-like fold, which was significantly lower than that in the WT, 23.00 (Supplementary Figure 3B). Together, all the *in vitro* and *in vivo* assays suggested that the secretion function of the MOECs was impaired by a deletion of the *Lgr4* gene.

### Deletion of *Lgr4* Altered the Expression of Genes Involved in Oviductal Epithelial Secretion

**FIGURE 4:**
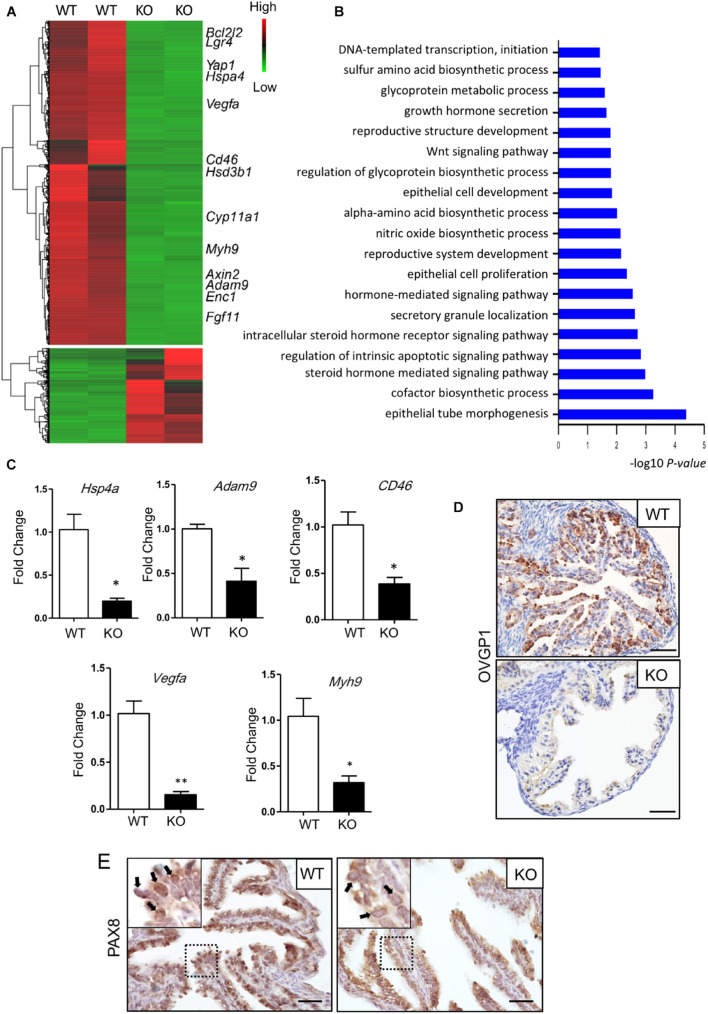
Molecular defects in the oviduct of *Lgr4*-deficient mice. **(A)** RNA-seq for mouse oviducts (the estrus stage). Clustered heat map of log2-transformed FPKMs showing the differentially expressed genes after LGR4 deletion. Indicated genes are marked on the right side of the heat map. **(B)** Gene Ontology analysis. **(C)** RT-PCR assay showing the relative changes of some representative small molecular factor genes. **(D)** IHC assays to detect the protein level of OVGP1 in the WT or *Lgr4*-deficient oviducts. **(E)** IHC assays to detect the location of PAX8 in the WT and *Lgr4*-deficient oviductal epithelial cells. ^∗^*p* ≤ 0.05; ^∗∗^*p* ≤ 0.01. Scale bar, 25 μm in **(D,E)**. RT-PCR, reverse transcription polymerase chain reaction; IHC, immunohistochemistry.

To reduce the influence of the estrus cycle on the transcriptom and to study the effect of *Lgr4* deficiency on the transcriptom, we analyzed the wild type and mutant oviducts in the estrus stage. The RNA-seq revealed a downregulation of 2,597 genes and an upregulation of 762 genes with *p*_*adj*_ value < 0.001, |log_2_fold change| > 2, and FPKM > 1 (Figure 4A and Supplementary Table 2). GO highlighted that differentially expressed genes (DEGs) were mainly involved in the regulation of epithelial tube morphogenesis; biosynthetic and secretory process, like sulfur amino acid, glycoprotein, and hormones; WNT signaling pathway; DNA-templated transcription, and others (Figure 4B). A set of secreted factor genes was checked between the WT and KO oviducts also at the estrus stage (Figures 4C,D). The mRNA level of *Hspa4*, the heat-shock protein family A (HSP70) member 4 (HSPA4), which is important for the sperm fertilizing ability, was downregulated significantly by 5.20-fold in the *Lgr4* mutant oviduct compared with the WT oviduct. The mRNA levels of disintegrin and metalloproteinase domain 9 (*Adam9*), and CD46 molecule (*CD46*), which were reported to have functions in the fertilization process, respectively (Alminana et al., 2017), were both significantly reduced by 2.42- and 2.64-fold. Another downregulated gene was *Vegfa*. VEGFA is a critical factor in the oviduct throughout the animal estrous cycle regulating the dynamics of oviduct fluid secretion and tubal contractibility. The mRNA of *Vegfa* was also detected to be significantly attenuated by 6.60-fold in the *Lgr4* mutant oviduct. Myosin-9, also known as MYH9, has been identified on human sperm as a binding partner of OVGP1 (Kadam et al., 2006). The mRNA level of *Myh9* was decreased significantly by 3.27-fold in the *Lgr4* mutant oviduct compared with the WT oviduct. Accordantly, we checked the protein level of OVGP1, observing that this protein was reduced significantly in the oviducts of *Lgr4* KO mice compared with the WT (Figure 4D). The IHC assay on the PAX8 protein showed specific 3,3′-diaminobenzidine (DAB) signals in the WT oviductal epithelial nuclei, while no apparent positive signals were observed in the KO mouse epithelial nuclei (Figure 4E, arrowed in black) at the stage of estrus. Collectively, the data demonstrated that *Lgr4* deficiency led to a hypofunction of oviducts *via* downregulating of genes linked to secretory processes.

### *Lgr4* Deficiency Attenuated the CTNNB1/NR5A2 Signaling in Oviductal Secretory Cells

**FIGURE 5:**
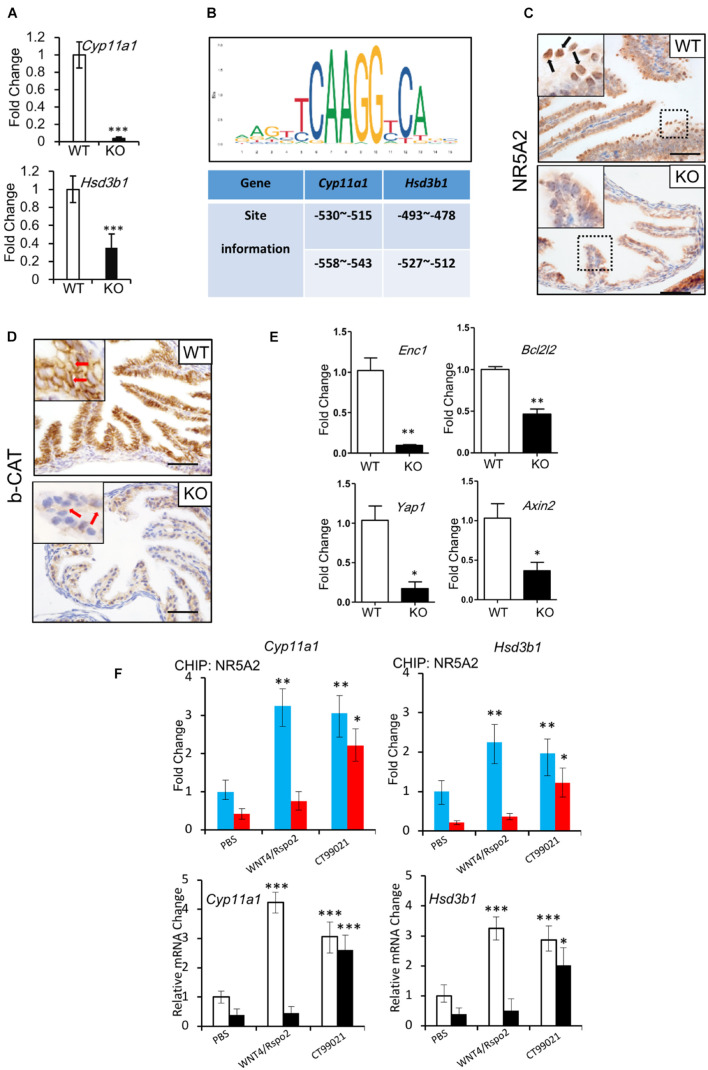
Regulation of NR5A2 activity by LGR4 mediated canonical WNT/CTNNB1 signaling pathway. **(A)** The mRNA levels of *Cyp11a1* and *Hsd3b1* in the WT and LGR4 mutant oviducts at the estrus stage. **(B)** Diagram of conserved NR5A1/2 binding sites in the *Cyp11a1* and *Hsd3b1* promoters predicted *in silico*. **(C)** IHC assays to detect the NR5A2 protein location in the WT or *Lgr4*-deficient oviducts. **(D)** IHC assays to detect the CTNNB1 protein level in the WT or *Lgr4*-deficient oviducts. **(E)** The mRNA levels of WNT target genes in WT and LGR4 mutant oviducts at estrus stage. **(F)** The chromatin of primary oviductal epithelial cells from WT or *Lgr4* mutant mice was immunoprecipitated with a NR5A2 antibody. Immunoprecipitated DNA was amplified with the sets of specific primers for the indicated NR5A2 binding sites in *Cyp11a1* and *Hsd3b1* promoters (upper panel). The relative fold change of *Cyp11a1* and *Hsd3b1* genes of the primary oviductal epithelial cells after the treatment of WNT4/RSPO2 or CT99201 (lower panel) are presented. ^∗^*p* ≤ 0.05; ^∗∗^*p* ≤ 0.01; ^∗∗∗^*p* ≤ 0.001. Scale bar, 50 μm in **(C,D)**. IHC, immunohistochemistry.

Hormone synthesis is predominantly controlled by members of the cytochrome P-450 (CYP) enzyme family and hydroxysteroid dehydrogenases (HSDs) (Chatuphonprasert et al., 2018). CYP11A1 and 3β-HSD are responsible for the formation of almost all the steroid hormones (Luu-The, 2013). Since we observed deficits in the secretory function of the oviducts of the *Lgr4* KO mice, we evaluated mRNA levels of the *Cyp11a1* and *3*β-*Hsd1* genes. The relative mRNA levels of the two genes were downregulated significantly (Figure 5A and Supplementary Table 2) at the stage of estrus. To determine how LGR4 regulated these steroidogenesis genes, we identified the WNT response elements in the *Cyp11a1* and *3*β-*Hsd1* gene promoters. By the method of *in silico* analysis,^2^ we found that the two genes had two putative highly conserved NR5A1/2 binding sites in the proximal promoter regions (Figure 5B). Although the NR5A1 and NR5A2 share the same DNA binding sequences, these two transcription factors work in a non-overlapping way. These might suggest that NR5A1 and NR5A2 are presented in different systems. To demonstrate, we checked the presence of NR5A1 and NR5A2 in oviducts, respectively. By the IHC assay, we detected the apparent presence of NR5A2 but not NR5A1 in the WT mouse oviductal epithelium. Note that the corresponding signal was reduced severely in the mutant oviductal epithelial nuclei (Figure 5C, arrowed in black and Supplementary Figures 4A,B). The mis-location of NR5A2 protein observed in nuclei implied an impairment of the DNA binding ability with the transcriptional factor NR5A2.

LGR4 has been widely considered as a WNT effector amplifier. To study if LGR4 has a similar function in our model, we compared the protein level of CTNNB1 between the WT and KO mice. The accumulation of CTNNB1 in the cytoplasm of cells was detectable in oviduct epithelial cells of WT mice at the stage of estrus. In contrast, the accumulation of CTNNB1 in *Lgr4* KO oviductal epithelial cells was significantly downregulated (Figure 5D, arrowed in red). In addition, a set of WNT/CTNNB1 signaling target genes like *Enc1* (Fujita et al., 2001), *Bcl2l2* (Lapham et al., 2009), *Yap1* (Konsavage et al., 2012), and *Axin2* (Yan et al., 2001; Jho et al., 2002; Lustig et al., 2002) were also downregulated significantly in the *Lgr4* KO oviducts compared with the WT control at the estrus stage (Figure 5E). These data further confirmed the regulatory function of LGR4 on the canonical WNT signaling pathway in the oviduct epithelium.

We hypothesized that the activated CTNNB1 might increase NR5A2 binding to the *Cyp11a1* and *3*β-*Hsd1* gene promoters in the mouse oviduct. To this end, we performed the ChIP-qPCR assay utilizing primary culture cells of the WT and *Lgr4* mutant mouse oviducts. In the WT, the capacity of the transcription factor NR5A2 to bind with *Cyp11a1* and *3*β-*Hsd1* promoter regions was improved by ∼3.26- and ∼2.25-fold, respectively, in response to the stimulation of factors WNT4/RSPO2. Such improved binding capacity of NR5A2 was not detectable in cultured cells of *Lgr4* mutant mice. However, CT99021, a commercial GSK-3α/β inhibitor that activates the canonical WNT pathway, enhanced the DNA binding abilities in WT and mutant primary oviductal epithelial cells. Specifically, in the WT group, the capacity of DNA binding to the *Cyp11a1* and *3*β-*Hsd1* promoters of the transcription factor NR5A2 was increased by ∼3.02- and ∼1.97-fold, respectively, while in the mutant group, such ability of NR5A2 to the *Cyp11a1* and *3*β-*Hsd1* promoters was rescued by ∼5.11- and ∼5.54-fold, respectively (Figure 5F, upper panel). The relative mRNA fold change of the *Cyp11a1* and *3*β-*Hsd1* genes after WNT4/RSPO2 or CT99021 application in the WT or *Lgr4* mutant oviductal epithelial cells further suggested the activity of the transcription factor NR5A2 was regulated by the canonical WNT cascade (Figure 5F, lower panel).

## Discussion

A previous study showed that the epithelial cell-specific [keratin-5 (K5) Cre] knockout of *Lgr4* in female mice resulted in subfertility. The researchers pointed out that this reproductive defect was due to alterations of epithelial differentiation characterized by a reduced uterine gland number (Sone et al., 2013). Although keratin-5 is expressed in the oviduct (Paik et al., 2012; Stadnicka et al., 2018), they did not mention the oviduct. The oviduct is derived from the Müllerian duct in mice (Stewart and Behringer, 2012). *Lgr4 K5-Cre* knockout reduced the numbers of fourfold and 8-cell–stage embryos and impaired embryo morphology at the blastocyst stage (Mohri et al., 2010). These observations indicated a potential involvement of *Lgr4* in the functionality of oviducts. In this present study, we elucidated the physiological role of LGR4 in oviducts of mice in greater detail. We showed that *Lgr4* was expressed in the epithelium of mouse oviducts and inducible at the estrus stage in ampulla region. Knockout of the *Lgr4* gene resulted in a deficiency of the mouse oviduct structure and led to oviductal hypo-secretion. This was mediated by the impairment of the CTNNB1 protein accumulation in the cytoplasm of the oviductal secretory cells. In addition, the *Lgr4* knockout caused a decrease in the WNT signaling that was required for the activation of the downstream transcriptional factor NR5A2.

The NR5A receptors are the orphan nuclear receptor proteins that act as transcription factors. They are evolutionarily conserved, as orthologs and paralogs are found in metazoans, from roundworms to mammals (Meinsohn et al., 2019). Despite the structural similarities between NR5A1 (steroidogenic factor-1 or SF-1) and NR5A2 (liver receptor homolog-1 or LRH-1), as well as their demonstrated ability to bind to the same DNA sequences, these two transcriptional factors are involved in the regulation of distinct physiological functions and often cannot compensate for each other (Duggavathi et al., 2008; Meinsohn et al., 2019). The two members are differentially expressed across a wide range of organs. NR5A1 exists in steroidogenic organs like the hypothalamus, pituitary gland, and testis. Both NR5A2 and NR5A1 regulated steroid synthesis, but the NR5A2 distribution was more widespread including in the liver (Xu et al., 2016; Stein et al., 2017; Miranda et al., 2018), pancreas (Benod et al., 2011; Holmstrom et al., 2011), intestine (Bayrer et al., 2015, 2018), ovary (Kawabe et al., 2013; Zhang et al., 2013; Bertolin et al., 2014), and uterus (Brosens et al., 2013; Mouzat et al., 2013). Here we showed the NR5A2, but not NR5A1, is localized in the mouse oviduct epithelium. According to the reported studies, both of the two transcription factors are co-activated by the CTNNB1. For example, NR5A1 and CTNNB1 interact in the signaling pathway to produce testosterone in Leydig cells (Jordan et al., 2003). NR5A2 interacts with CTNNB1 to promote cell cycle-dependent gene expression and regulates cell proliferation in the intestinal crypt and granulosa cells (Botrugno et al., 2004; Meinsohn et al., 2018). In addition, we show that the transcriptional control by NR5A2 of steroidogenesis required the activation of the canonical WNT pathway in oviductal epithelial cells.

The canonical WNT pathway occupies a multitude of essential roles in cell fate and tissue homeostasis. Misregulation of WNT signaling has been associated with a variety of human diseases (Clevers, 2006; Clevers and Nusse, 2012; Nusse and Clevers, 2017). Research using patient tissues detected that the CTNNB1 expression was enhanced in the oviductal epithelium in ectopic tubal pregnancies. The ectopic implantation of embryos escalated the canonical WNT signaling, which changed the environment in the fallopian tube, such as reduced E-cadherin, induced angiogenesis, and glycogen accumulation (Li et al., 2013). In this present study, we demonstrated that insufficient WNT caused secretory deficiencies in the oviduct. Hence, our study, together with previous reports, suggested that the WNT cascade required a precise regulation in the fallopian tube. Unlike *Lgr5* and *Lgr6*, we showed the *Lgr4* gene was expressed broadly in the mouse oviduct epithelium. And the RNA-seq data also suggested the level of *Lgr4* was much higher than those of *Lgr5* and *Lgr6*. Knockout of *Lgr4* severely reduced the number of secretory cells. Interestingly, the mRNA-seq revealed insignificant changes in the levels of *Lgr5* and *Lgr6*. Taking into the account that the WNT/CTNNB1 signaling is essential for the self-renewal of secretory cells in the oviduct and our data, we conclude that LGR4 but not LGR5 or LGR6 controls the canonical WNT signaling pathway in the secretory cells.

**FIGURE 6:**
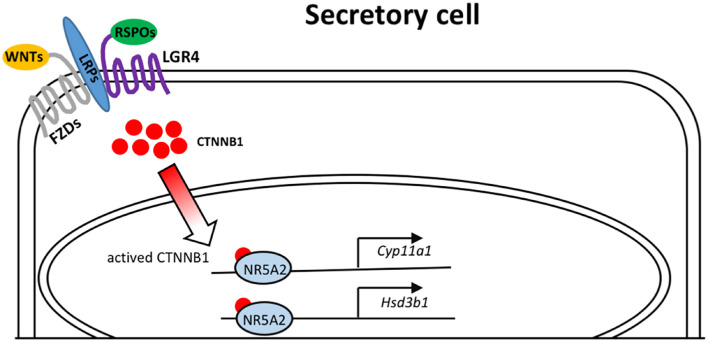
Model depicting the molecular mechanisms of LGR4 mediated WNT signaling that activates the transcription factor NR5A2 to bind on the promoters of the steroidogenic genes, *Cyp11a1* and *Hsd3b1*.

In summary, we presented data indicating a dominant role of *Lgr4* in the regulation of oviductal epithelial secretion (Figure 6). As the fallopian tube pathologies underlie a total number of 30% of infertile women worldwide (Briceag et al., 2015), identifying the essential roles of LGR4 in these patients represents an advancement that has implications for the development of diagnostic tools and as a potential drug target to treat fallopian tube secretion insufficiency.

## Data Availability Statement

The datasets presented in this study can be found in online repositories. The names of the repository/repositories and accession number(s) can be found below: BioProject (PRJNA691984) in NCBI, https://www.ncbi.nlm.nih.gov/bioproject/PRJNA691984.

## Ethics Statement

The animal study was reviewed and approved by the Institutional Ethics Committee of Shanghai Institute of Planned Parenthood Research Center (Shanghai Institute of Biomedical and Pharmaceutical Technologies now).

## Author Contributions

XT carried out all the experiments and drafted the manuscript. LZ carried out all the analysis of bio-informatics data. TL, JZ, and KQ maintained and genotyped the mice. HW and SS prepared the slides and carried out the PAS, Alcian blue, HE, and IHC staining. MH and FZ performed the isolated and maintained the mouse tubal epithelial cells. MZ and CL carried out the oocyte–epithelial co-culture assay. RL and HP provided critical suggestions, designed the research, and supervised the project. All authors contributed to the article and approved the submitted version.

## Conflict of Interest

The authors declare that the research was conducted in the absence of any commercial or financial relationships that could be construed as a potential conflict of interest.

## Publisher’s Note

All claims expressed in this article are solely those of the authors and do not necessarily represent those of their affiliated organizations, or those of the publisher, the editors and the reviewers. Any product that may be evaluated in this article, or claim that may be made by its manufacturer, is not guaranteed or endorsed by the publisher.

## Footnotes

1
http://www.ensembl.org/index.html

2
http://jaspar.genereg.net/

## Supplementary Material

Supplementary Figure 1 The mRNA level of *Lgr5* and *Lgr6* in mouse oviducts. **(A)** The mRNA levels of *Lgr5* in the specific regions of the oviduct. **(B)** The mRNA levels of *Lgr6* in the specific regions of the oviduct. At least three independent experiments were carried out. Data were presented as mean ± SEM. NS > 0.05.

Supplementary Figure 2 Loss of LGR4 led to histological abnormalities of oviducts. **(A)** Histological analysis of the WT and *Lgr4* KO oviducts were performed by hematoxylin and eosin staining after the supplement of E2. **(B)** The average number of leaf-like folds per section after the supplement of E2. **(C)** The average number of the secretory cells per leaf-like fold after the supplement of E2. ^∗^*p* ≤ 0.05; ^∗∗∗^*p* ≤ 0.001. Scale bar, 50 μm.

Supplementary Figure 3 Loss of LGR4 led to a deficiency in oviductal secretion. **(A)** The bar graphs summarized the number of positively labeled cells of PAS stained oviduct slides. **(B)** The number of the positively Alcian blue stained cells in oviducts were presented. At least three independent experiments were carried out. ^∗∗∗^*p* ≤ 0.001.

Supplementary Figure 4 Detection of NR5A1 protein in mouse oviduct. **(A)** IHC assay of NR5A1 in the WT and *Lgr4* knockout mouse oviducts. **(B)** IHC assay of NR5A1 in the WT testis as control. Note, the positive signals in the enclosed area by red dashed lines. Scale bar, 50 μm.

Supplementary Table 1 Primer list.

Supplementary Table 2 Differential expressed genes between the *Lgr4* deletion and control oviducts. Differential gene expression analysis between *Lgr4* deletion and control oviducts (the estrus stage) revealed a number of significantly different expressed genes.
