# Supplementary figures and images for: Lgr4 Regulates Oviductal Epithelial Secretion Through the WNT Signaling Pathway
Source: Front Cell Dev Biol. 2021 Sep 24;9:666303. doi: 10.3389/fcell.2021.666303 (PMC8497904; doi:10.3389/fcell.2021.666303)

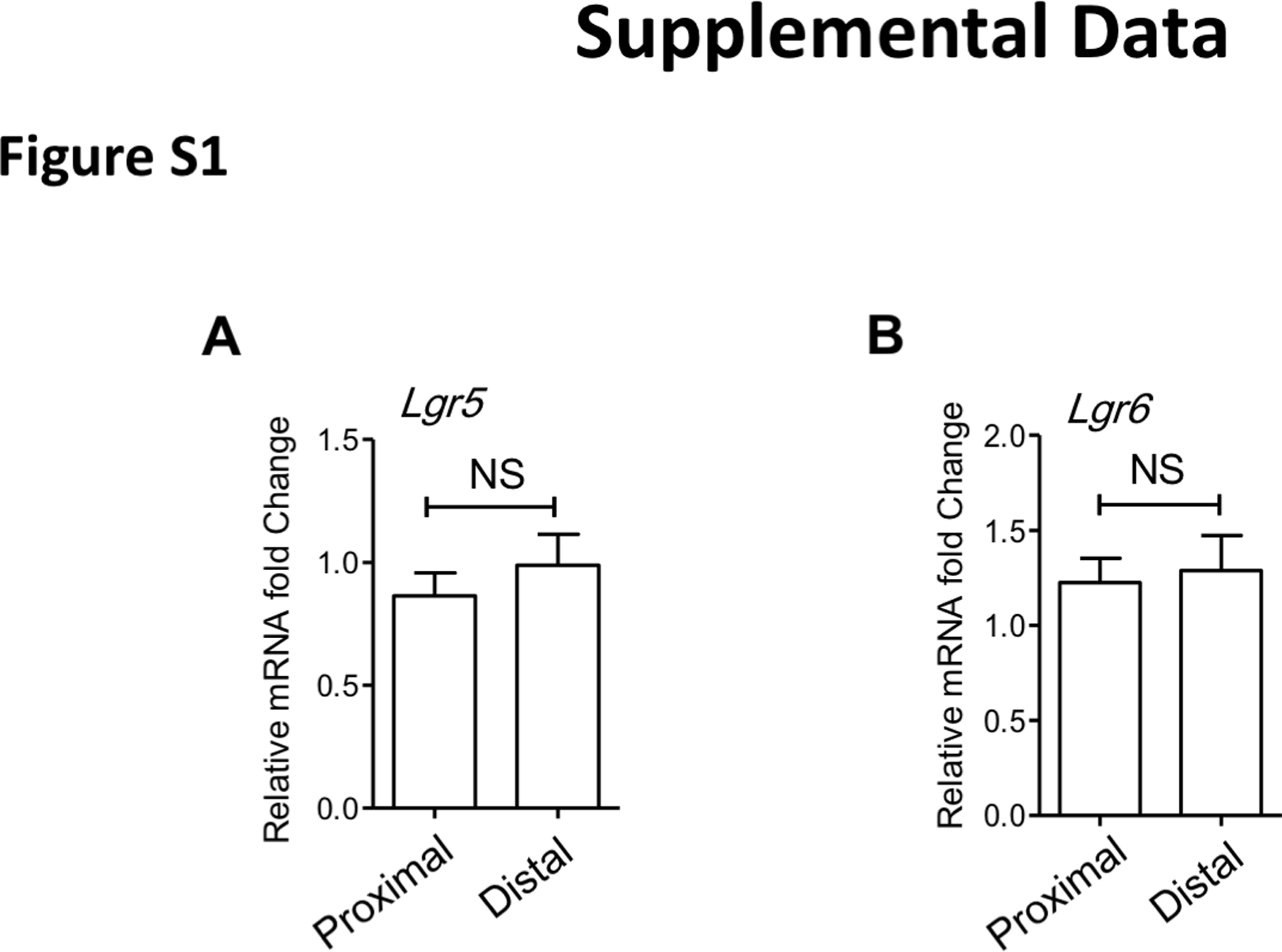

Supplement: Supplementary Figure 1 — The mRNA level of Lgr5 and Lgr6 in mouse oviducts. (A) The mRNA levels of Lgr5 in the specific regions of the oviduct. (B) The mRNA levels of Lgr6 in the specific regions of the oviduct. At least three independent experiments were carried out. Data were presented as mean ± SEM. NS > 0.05. [file Image_1.TIFF]

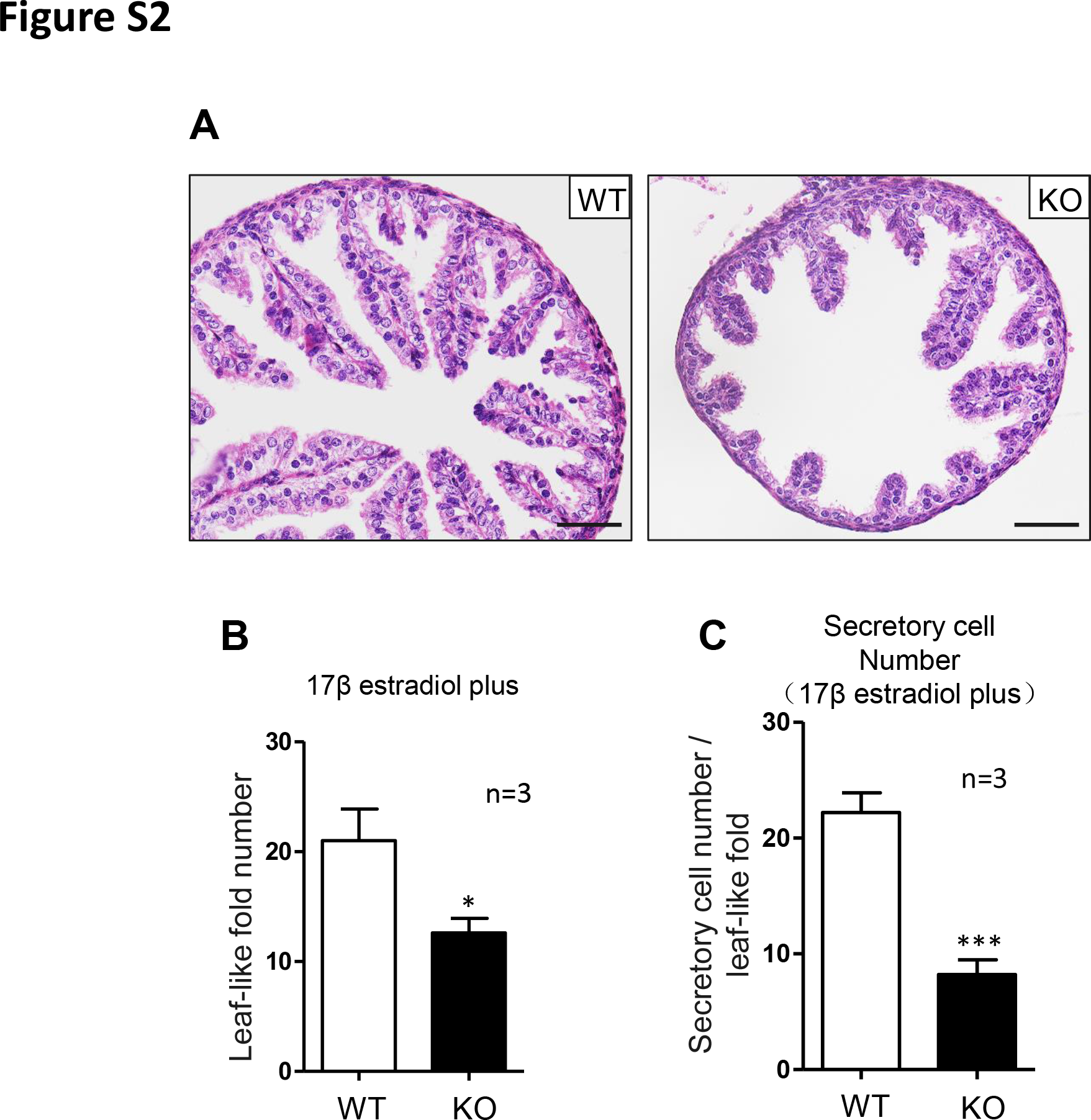

Supplement: Supplementary Figure 2 — Loss of LGR4 led to histological abnormalities of oviducts. (A) Histological analysis of the WT and Lgr4 KO oviducts were performed by hematoxylin and eosin staining after the supplement of E2. (B) The average number of leaf-like folds per section after the supplement of E2. (C) The average number of the secretory cells per leaf-like fold after the supplement of E2. ∗p ≤ 0.05; ∗∗∗p ≤ 0.001. Scale bar, 50 μm. [file Image_2.tif]

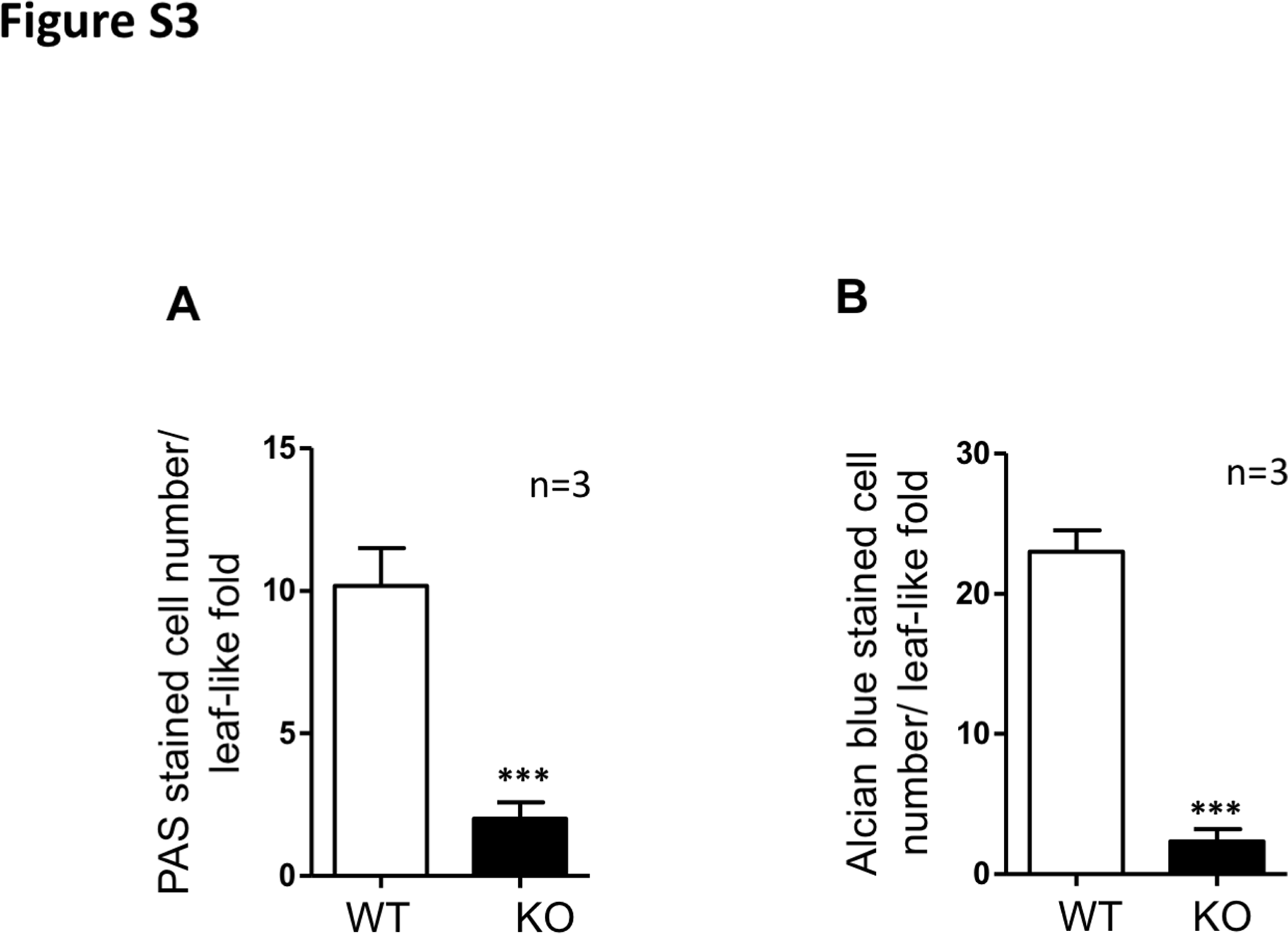

Supplement: Supplementary Figure 3 — Loss of LGR4 led to a deficiency in oviductal secretion. (A) The bar graphs summarized the number of positively labeled cells of PAS stained oviduct slides. (B) The number of the positively Alcian blue stained cells in oviducts were presented. At least three independent experiments were carried out. ∗∗∗p ≤ 0.001. [file Image_3.TIFF]

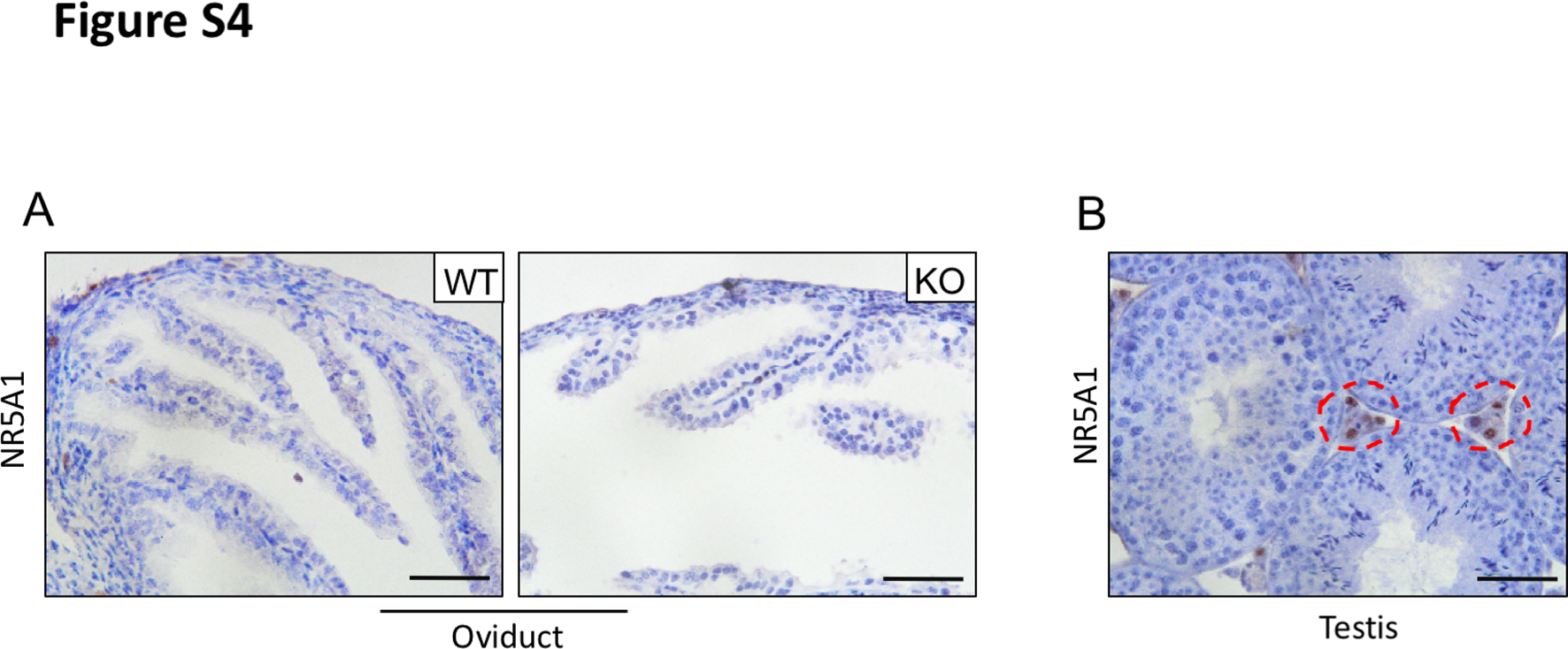

Supplement: Supplementary Figure 4 — Detection of NR5A1 protein in mouse oviduct. (A) IHC assay of NR5A1 in the WT and Lgr4 knockout mouse oviducts. (B) IHC assay of NR5A1 in the WT testis as control. Note, the positive signals in the enclosed area by red dashed lines. Scale bar, 50 μm. [file Image_4.TIFF]
