# Supplementary material for: Lgr4 Regulates Oviductal Epithelial Secretion Through the WNT Signaling Pathway
Source: Front Cell Dev Biol. 2021 Sep 24;9:666303. doi: 10.3389/fcell.2021.666303 (PMC8497904; doi:10.3389/fcell.2021.666303)
Supplement: Supplementary Table 1 — Primer list. [file Table_1.DOCX]

**Table S 1.**

| **Primer list** | | |
| --- | --- | --- |
| **Primers for genotyping** | | |
| *Lgr4* | AAGCACTTGATGGTCAGACTACATGC | sense |
|  | AAAAGCCACATTCAAATCTTAGTAACC | anti-sense 1 |
|  | GGT CTT TGA GCA CCA GAG GACATC | anti-sense2 |
| **Primers for RT-PCR** | | |
| *Lgr4* | AAGAACTCAAAGTCCTAACC | sense |
|  | CAGATGCCGCAACTGAACGA | anti-sense |
|  |  |  |
| *Lgr5* | GCCTCGCACATTACTGAA | sense |
|  | GGATGATAGGTCCAACTTTA | anti-sense |
|  |  |  |
| *Lgr6* | CCGCCACATCCCTGACTA | sense |
|  | TGCTGACCTTCCCACAAAC | anti-sense |
|  |  |  |
| *Fgf11* | TCCAAGGTGCGACTGTGC | sense |
|  | AACGACGCTGACGGTAGA | anti-sense |
|  |  |  |
| *Vegfa* | CGATGAAGCCCTGGAGTG | sense |
|  | ATGATGGCGTGGTGGTGA | anti-sense |
|  |  |  |
| *Cd46* | CTGTCGCAGAAGGAAGAG | sense |
|  | CATGTATGATTTGGCTCA | anti-sense |
|  |  |  |
| *Adam9* | ATTCGCTTAGCAAACTACCT | sense |
|  | TCCTACAAACGCCATTCC | anti-sense |
|  |  |  |
| *Hspa4* | AGCCAGGTAATTTCTAATG | sense |
|  | TAGGAACCGAGACAACAC | anti-sense |
|  |  |  |
| *Myh9* | AAACGCCAAGACGGTGAAGA | sense |
|  | CAGGAAGCGGTATTTGTTGTAT | anti-sense |
|  |  |  |
| *Cyp11a1* | TTCTGGACACCACCACTTCA | sense |
|  | AGGACGATTCGGTCTTTCTT | anti-sense |
|  |  |  |
| *Hsd3b1* | GGACAAAGTATTCCGACCAG | sense |
|  | TTGCTGTATGGGTATGGATC | anti-sense |
|  |  |  |
| *Enc1* | CCAGTGGACCAAGGTGGGAGA | sense |
|  | CAGGTGCTGACGAATGCAGTAGG | anti-sense |
|  |  |  |
| *Bcl2l2* | GCCGTCTTGTGGCATTCTTT | sense |
|  | AGGGCATTCGCTATTGTTCC | anti-sense |
|  |  |  |
| *Yap1* | TCCTCCTTTGAGATCCCTGAT | sense |
|  | TGTATTTGCTGCTGCTGGTT | anti-sense |
| *Axin2* | GACGCACTGACCGACGATTC | sense |
|  | CCAGACTATGGCGGCTTTCC | anti-sense |
| **Primers for CHIP--qPCR** | | |
| *Cyp11a1* | ACCGTCTACTGGCTGCCCGGC | sense |
|  | TTAGCCATACCTTCCTCTACCCT | anti-sense |
|  |  |  |
| *Hsd3b1* | TCTGAAATGGATGGAAATAACTT | sense |
|  | CTATAGGTTAGGAATTAATGTTC | anti-sense |
