# Supplementary material for: Lgr4 Regulates Oviductal Epithelial Secretion Through the WNT Signaling Pathway
Source: Front Cell Dev Biol. 2021 Sep 24;9:666303. doi: 10.3389/fcell.2021.666303 (PMC8497904; doi:10.3389/fcell.2021.666303)
Supplement: Supplementary Table 2 — Differential expressed genes between the Lgr4 deletion and control oviducts. Differential gene expression analysis between Lgr4 deletion and control oviducts (the estrus stage) revealed a number of significantly different expressed genes. [file Table_2.DOCX]

**Table S2 Differential expressed genes between the *Lgr4* deletion and control oviduct**

| **Symbol** | **KO/WT_log_2_FC** | ***p-*value** |
| --- | --- | --- |
| *Syngap1* | -9.8219 | 7.249E-47 |
| *Gm20716* | -9.54514 | 1.198E-35 |
| *Gm28048* | -9.50427 | 6.869E-17 |
| *Brsk1* | -8.65684 | 2.423E-16 |
| *Pla2g4b* | -8.64177 | 5.376E-17 |
| *Foxo4* | -8.58584 | 1.481E-27 |
| *Stac3* | -8.52912 | 5.392E-13 |
| *Npas4* | -8.39786 | 4.345E-18 |
| *Ccdc150* | -8.31171 | 2.096E-17 |
| *Gm9821* | -8.21332 | 6.146E-14 |
| *Ugt1a5* | -8.16691 | 5.336E-11 |
| *4930562C15Rik* | -8.15185 | 1.723E-11 |
| *Gm49601* | -8.05142 | 1.296E-13 |
| *Lmtk3* | -7.98279 | 5.310E-15 |
| *Zscan2* | -7.85663 | 4.142E-10 |
| *Cacng7* | -7.82187 | 1.633E-12 |
| *1700020D05Rik* | -7.68392 | 7.973E-11 |
| *Defb23* | -7.54632 | 5.172E-09 |
| *Syt2* | -7.5317 | 8.284E-07 |
| *Gpr162* | -7.50737 | 9.718E-11 |
| *Arl9* | -7.47562 | 3.840E-08 |
| *Hist2h3c1* | -7.27448 | 2.310E-11 |
| *Peg10* | -7.23797 | 4.219E-15 |
| *Obox2* | -7.23039 | 1.240E-10 |
| *St6galnac2* | -7.142 | 2.140E-11 |
| *Fam228b* | -7.10794 | 1.122E-07 |
| *Naip6* | -7.10125 | 2.498E-19 |
| *Gm28230* | -7.07382 | 1.180E-09 |
| *Thbs4* | -7.01715 | 1.851E-06 |
| *Mdfi* | -6.97572 | 2.146E-07 |
| *Nr5a2* | -6.94682 | 9.047E-04 |
| *Nyap1* | -6.90108 | 3.223E-10 |
| *Adam1a* | -6.89537 | 5.135E-12 |
| *5730596B20Rik* | -6.89185 | 2.323E-14 |
| *Lrp5* | -6.87891 | 1.073E-13 |
| *Mtx3* | -6.87814 | 1.079E-26 |
| *Gm38393* | -6.87571 | 5.179E-11 |
| *Zbed6* | -6.84148 | 7.690E-16 |
| *Cacna1d* | -6.77512 | 1.011E-18 |
| *Mss51* | -6.76556 | 9.519E-11 |
| *Rnft2* | -6.76453 | 5.716E-05 |
| *Rho* | -6.70836 | 1.239E-09 |
| *Sspo* | -6.70498 | 3.542E-10 |
| *Taf15* | -6.67101 | 1.063E-16 |
| *Cemip* | -6.63449 | 6.586E-14 |
| *Mroh7* | -6.62806 | 1.303E-10 |
| *Srcin1* | -6.6197 | 4.103E-15 |
| *Tatdn2* | -6.56444 | 1.514E-12 |
| *Plau* | -6.49364 | 1.074E-08 |
| *Gm3045* | -6.43657 | 2.590E-05 |
| *Ap1s3* | -6.4064 | 1.268E-10 |
| *Khsrp* | -6.40454 | 1.026E-22 |
| *Rxfp1* | -6.37971 | 7.322E-26 |
| *Dnah3* | -6.36212 | 2.575E-20 |
| *Dnah10* | -6.29861 | 1.079E-17 |
| *Plxna3* | -6.26921 | 1.944E-11 |
| *Fat3* | -6.22427 | 4.727E-20 |
| *Sema7a* | -6.19484 | 4.172E-05 |
| *Arglu1* | -6.17303 | 4.693E-20 |
| *Hsd3b1* | -6.15521 | 8.628E-08 |
| *Cyp11a1* | -6.14428 | 1.055E-15 |
| *Ybx2* | -6.11862 | 1.261E-07 |
| *Snhg11* | -6.11196 | 3.493E-14 |
| *Gm16485* | -6.05902 | 1.862E-15 |
| *Mmp10* | -6.05456 | 2.285E-14 |
| *Hhip* | -6.04487 | 1.415E-10 |
| *Gm21981* | -6.0296 | 4.757E-12 |
| *Gm45140* | -6.01245 | 1.374E-07 |
| *Myom1* | -6.00222 | 6.081E-24 |
| *Adamts6* | -5.99444 | 8.792E-14 |
| *Kcnc3* | -5.96464 | 4.036E-08 |
| *Ddx17* | -5.96056 | 1.383E-17 |
| *Cacna1c* | -5.95436 | 8.547E-13 |
| *Cxcr4* | -5.92541 | 6.057E-07 |
| *Tbc1d8b* | -5.91133 | 1.193E-15 |
| *Carmil3* | -5.90557 | 7.885E-07 |
| *Cap1* | -5.90379 | 1.094E-16 |
| *Cfap65* | -5.89943 | 1.376E-19 |
| *Mink1* | -5.89194 | 2.875E-19 |
| *Nfya* | -5.88161 | 6.529E-19 |
| *Gpc2* | -5.8679 | 7.031E-09 |
| *Aoc2* | -5.85694 | 4.640E-09 |
| *Hmbox1* | -5.84489 | 2.128E-15 |
| *Myh11* | -5.83665 | 2.448E-18 |
| *Tmem200c* | -5.82752 | 1.062E-04 |
| *Gsk3a* | -5.82543 | 3.852E-19 |
| *Tgfbr3l* | -5.81248 | 1.874E-08 |
| *Sh3pxd2a* | -5.80193 | 1.093E-17 |
| *Hoxa3* | -5.79367 | 1.021E-14 |
| *Pbx2* | -5.79163 | 1.917E-23 |
| *Luc7l2* | -5.77954 | 4.693E-20 |
| *Cdc42bpg* | -5.77387 | 1.319E-19 |
| *Muc3a* | -5.76536 | 2.057E-11 |
| *Msi1* | -5.76501 | 1.671E-10 |
| *Hoxa6* | -5.76224 | 6.258E-09 |
| *Cpeb4* | -5.76031 | 3.315E-14 |
| *Pip4k2b* | -5.75814 | 4.033E-17 |
| *Gm3636* | -5.74822 | 4.684E-06 |
| *Tbx6* | -5.73851 | 1.216E-06 |
| *Ankrd52* | -5.73634 | 7.062E-09 |
| *Greb1l* | -5.72888 | 3.980E-08 |
| *Prss35* | -5.72854 | 7.658E-06 |
| *Pgpep1l* | -5.71535 | 3.139E-06 |
| *Actn1* | -5.71292 | 1.582E-15 |
| *Fus* | -5.70708 | 7.583E-14 |
| *Hoxa2* | -5.69708 | 2.882E-09 |
| *Slc8a2* | -5.69153 | 4.689E-08 |
| *Mcf2* | -5.69106 | 6.532E-09 |
| *Itga10* | -5.68598 | 3.607E-06 |
| *Fbxl19* | -5.68438 | 4.727E-20 |
| *Smcr8* | -5.67804 | 2.700E-17 |
| *Dnah11* | -5.67681 | 2.717E-17 |
| *Vegfa* | -5.66988 | 6.895E-18 |
| *Fbxw24* | -5.66013 | 1.826E-06 |
| *Lama1* | -5.65908 | 2.169E-12 |
| *Col6a4* | -5.65454 | 8.671E-12 |
| *Frem1* | -5.64274 | 5.405E-09 |
| *Ccl28* | -5.62885 | 4.667E-06 |
| *Tspoap1* | -5.62514 | 1.002E-08 |
| *Atf7ip2* | -5.62429 | 3.293E-06 |
| *Gli1* | -5.62398 | 2.033E-17 |
| *Apba1* | -5.60003 | 7.313E-12 |
| *Zfc3h1* | -5.59976 | 2.430E-13 |
| *Rtl5* | -5.585 | 1.167E-04 |
| *Tarbp1* | -5.58375 | 1.289E-10 |
| *Bend3* | -5.56502 | 9.457E-10 |
| *Kcnj2* | -5.55441 | 1.108E-09 |
| *Trio* | -5.54809 | 1.748E-15 |
| *Duxbl1* | -5.54359 | 1.470E-05 |
| *Clec2d* | -5.52532 | 1.551E-13 |
| *Ccdc187* | -5.52494 | 2.360E-15 |
| *Cntnap1* | -5.50996 | 5.825E-13 |
| *Plin4* | -5.49804 | 3.696E-07 |
| *Tfap4* | -5.46976 | 4.781E-04 |
| *Ppp1r3f* | -5.46821 | 4.353E-06 |
| *Nwd1* | -5.46418 | 1.318E-12 |
| *Elf5* | -5.45344 | 1.816E-06 |
| *Tgm4* | -5.44522 | 1.252E-06 |
| *Fnbp4* | -5.44018 | 8.617E-14 |
| *Prrc2a* | -5.43257 | 3.179E-16 |
| *Gcnt4* | -5.40336 | 8.349E-08 |
| *Rsad1* | -5.40021 | 7.935E-16 |
| *Fam227a* | -5.3962 | 4.617E-12 |
| *Ptpn20* | -5.3951 | 3.406E-04 |
| *Pgm5* | -5.3945 | 9.628E-15 |
| *Zscan25* | -5.38274 | 6.128E-10 |
| *Marf1* | -5.37431 | 9.624E-15 |
| *Map2* | -5.36345 | 1.742E-11 |
| *Spaca6* | -5.36037 | 2.582E-14 |
| *Asb14* | -5.34034 | 3.440E-08 |
| *Lzts3* | -5.33874 | 6.541E-18 |
| *Klc2* | -5.32748 | 1.004E-07 |
| *Zbtb34* | -5.32199 | 3.128E-06 |
| *Clip2* | -5.32019 | 6.169E-07 |
| *Dhtkd1* | -5.31486 | 8.594E-05 |
| *Fat4* | -5.30494 | 3.409E-10 |
| *Gm35339* | -5.3045 | 8.460E-07 |
| *Dhx9* | -5.30437 | 1.050E-17 |
| *U2af2* | -5.27673 | 3.327E-17 |
| *Mtss1l* | -5.26626 | 8.051E-07 |
| *Dgkh* | -5.25795 | 5.941E-17 |
| *Kcng3* | -5.24868 | 7.431E-08 |
| *Dcaf6* | -5.24194 | 2.280E-19 |
| *Hoxc6* | -5.23668 | 2.942E-13 |
| *Dlg4* | -5.2347 | 3.860E-18 |
| *Plekhg2* | -5.23462 | 4.659E-13 |
| *Bak1* | -5.2342 | 2.141E-09 |
| *Spred3* | -5.22324 | 2.630E-10 |
| *Kmt2b* | -5.22061 | 3.241E-11 |
| *Epm2aip1* | -5.21889 | 7.006E-18 |
| *Cramp1l* | -5.2141 | 1.550E-18 |
| *Adcy6* | -5.21348 | 1.854E-15 |
| *Aspm* | -5.19289 | 5.966E-05 |
| *Ppm1e* | -5.18542 | 2.525E-14 |
| *Fzd5* | -5.18261 | 1.293E-08 |
| *Dnah6* | -5.18034 | 9.440E-12 |
| *Leng8* | -5.17636 | 1.532E-14 |
| *Gnao1* | -5.15712 | 3.376E-12 |
| *Mapkbp1* | -5.15187 | 3.787E-16 |
| *Eml5* | -5.14954 | 1.605E-04 |
| *Sned1* | -5.14726 | 1.011E-10 |
| *Zfp146* | -5.14103 | 2.083E-14 |
| *Gm13199* | -5.13871 | 9.358E-04 |
| *Dlec1* | -5.1364 | 4.540E-09 |
| *Szt2* | -5.136 | 1.546E-14 |
| *Pcnx* | -5.1295 | 1.465E-13 |
| *Zfp609* | -5.12872 | 5.979E-15 |
| *Slmap* | -5.12738 | 5.017E-12 |
| *Slc38a5* | -5.09935 | 6.961E-04 |
| *Slk* | -5.09908 | 4.709E-14 |
| *Rbm41* | -5.0853 | 5.454E-08 |
| *Vmn2r29* | -5.08426 | 9.886E-04 |
| *Cfap43* | -5.07919 | 4.607E-12 |
| *Pla2g3* | -5.07845 | 4.759E-07 |
| *Zbtb12* | -5.07295 | 3.318E-08 |
| *Asah2* | -5.07224 | 3.360E-12 |
| *Npcd* | -5.06972 | 3.341E-05 |
| *Chdh* | -5.06154 | 1.972E-12 |
| *Brwd3* | -5.05211 | 2.413E-13 |
| *Lgr4* | -5.04752 | 4.229E-16 |
| *Insyn1* | -5.04345 | 3.905E-04 |
| *Nav1* | -5.03785 | 9.536E-08 |
| *Noc3l* | -5.03406 | 8.734E-12 |
| *Fam151b* | -5.0307 | 4.966E-07 |
| *Kmt2a* | -5.02515 | 2.955E-16 |
| *Tnk2* | -5.02079 | 3.972E-14 |
| *Fat1* | -5.01645 | 2.019E-10 |
| *Bcl9l* | -5.0095 | 2.080E-09 |
| *Sec14l5* | -5.00609 | 2.832E-09 |
| *Med13* | -4.98669 | 7.133E-18 |
| *Kif26a* | -4.98608 | 2.488E-07 |
| *Purg* | -4.98456 | 9.488E-07 |
| *Nt5dc3* | -4.98239 | 3.867E-11 |
| *Crocc2* | -4.98165 | 2.717E-17 |
| *Clmn* | -4.98131 | 8.957E-13 |
| *Wasf3* | -4.97853 | 3.693E-09 |
| *Ube2d2b* | -4.97733 | 1.062E-05 |
| *Cacna1g* | -4.96879 | 4.063E-05 |
| *Ntrk3* | -4.96258 | 9.461E-18 |
| *Myo5c* | -4.95234 | 4.760E-16 |
| *Frmd7* | -4.945 | 2.883E-04 |
| *Rbm12b2* | -4.9415 | 4.555E-09 |
| *Mllt6* | -4.94089 | 8.792E-14 |
| *Pcdhga12* | -4.93991 | 2.720E-06 |
| *Lrp2* | -4.93585 | 4.488E-12 |
| *Nlgn2* | -4.93424 | 1.249E-16 |
| *Avil* | -4.92782 | 1.704E-06 |
| *Hnrnpul2* | -4.92046 | 1.250E-14 |
| *Tial1* | -4.91434 | 8.257E-15 |
| *Zfp280c* | -4.91404 | 2.124E-11 |
| *Kndc1* | -4.90839 | 5.799E-15 |
| *Edem3* | -4.89928 | 7.769E-16 |
| *Gabbr1* | -4.89792 | 1.358E-18 |
| *Tjp1* | -4.8943 | 1.857E-16 |
| *Nup205* | -4.89258 | 1.259E-11 |
| *Lbh* | -4.89109 | 3.829E-17 |
| *Zfp641* | -4.88552 | 1.395E-07 |
| *Sema4g* | -4.88464 | 9.416E-06 |
| *Zc3h11a* | -4.88016 | 4.553E-16 |
| *Olig3* | -4.87447 | 1.364E-04 |
| *1700109H08Rik* | -4.86813 | 4.018E-07 |
| *Gigyf1* | -4.86711 | 1.447E-11 |
| *Arhgef11* | -4.86541 | 2.340E-11 |
| *Ints6l* | -4.86119 | 1.975E-16 |
| *Akr1cl* | -4.85916 | 6.037E-04 |
| *Mdc1* | -4.85891 | 5.714E-14 |
| *Itpr1* | -4.84771 | 7.469E-10 |
| *Plxnd1* | -4.84625 | 1.097E-09 |
| *Cadm4* | -4.84059 | 2.385E-06 |
| *Sema6c* | -4.8402 | 1.254E-04 |
| *Socs7* | -4.83987 | 7.724E-14 |
| *Slc5a3* | -4.83707 | 4.488E-12 |
| *Pitpnm3* | -4.83521 | 1.958E-09 |
| *Mical3* | -4.82253 | 8.143E-13 |
| *Braf* | -4.82208 | 1.149E-17 |
| *Gon4l* | -4.81745 | 2.578E-10 |
| *Sp1* | -4.8168 | 1.645E-16 |
| *Celsr1* | -4.81637 | 1.741E-14 |
| *Phf2* | -4.81356 | 1.809E-13 |
| *Faxc* | -4.81354 | 1.180E-07 |
| *Gucy1b1* | -4.81242 | 6.741E-08 |
| *Igip* | -4.81097 | 2.080E-09 |
| *Upf1* | -4.81042 | 1.744E-14 |
| *Etnk1* | -4.80994 | 1.606E-12 |
| *Gabpb2* | -4.80529 | 3.813E-16 |
| *Hectd2* | -4.80424 | 1.175E-07 |
| *Hoxb4* | -4.80176 | 2.513E-09 |
| *Adamts12* | -4.80128 | 2.839E-13 |
| *Grem2* | -4.80006 | 7.198E-08 |
| *Arhgef9* | -4.79397 | 5.521E-09 |
| *Pik3c2a* | -4.79313 | 7.401E-09 |
| *Mtr* | -4.79211 | 3.281E-11 |
| *Stxbp5* | -4.7914 | 1.087E-07 |
| *Ubr5* | -4.78984 | 9.058E-16 |
| *Gmnc* | -4.78797 | 1.518E-09 |
| *Taok2* | -4.7822 | 1.092E-11 |
| *Abca5* | -4.7816 | 2.501E-09 |
| *Postn* | -4.78073 | 1.215E-12 |
| *Slc45a4* | -4.78041 | 1.958E-09 |
| *Nckap5l* | -4.7801 | 3.154E-07 |
| *Fam208a* | -4.77868 | 4.454E-13 |
| *Xpo1* | -4.77825 | 3.828E-12 |
| *Slc30a4* | -4.77309 | 1.245E-11 |
| *Serac1* | -4.7721 | 1.879E-05 |
| *Rev3l* | -4.77083 | 2.869E-12 |
| *Fam8a1* | -4.76168 | 6.235E-13 |
| *Lsm14b* | -4.75919 | 1.252E-11 |
| *Dusp11* | -4.75459 | 1.177E-10 |
| *Sytl2* | -4.75359 | 9.416E-06 |
| *Slco5a1* | -4.75278 | 2.506E-07 |
| *Zfp595* | -4.75 | 1.275E-08 |
| *Kcnc2* | -4.74653 | 2.238E-04 |
| *Lama3* | -4.74414 | 1.058E-09 |
| *Catsperg1* | -4.73888 | 1.326E-05 |
| *Zfp46* | -4.73776 | 5.160E-15 |
| *Scube3* | -4.73755 | 1.031E-04 |
| *Pdzd4* | -4.73709 | 4.370E-08 |
| *Hydin* | -4.73534 | 9.624E-15 |
| *Mdm4* | -4.73441 | 1.990E-14 |
| *Syne1* | -4.73403 | 2.805E-14 |
| *Zdhhc23* | -4.73025 | 1.184E-07 |
| *Sema3g* | -4.72998 | 2.677E-06 |
| *Dock9* | -4.72805 | 1.784E-13 |
| *Mib1* | -4.72319 | 1.088E-14 |
| *Slc5a11* | -4.72244 | 9.335E-05 |
| *Crebzf* | -4.72233 | 3.854E-11 |
| *Slc39a14* | -4.72001 | 5.206E-15 |
| *Trp53inp1* | -4.71741 | 1.719E-11 |
| *Dst* | -4.7161 | 4.128E-11 |
| *Chmp4b* | -4.71526 | 1.635E-15 |
| *Vps54* | -4.71516 | 3.918E-14 |
| *Mybpc1* | -4.70764 | 1.414E-13 |
| *Wdr66* | -4.69313 | 7.583E-14 |
| *Isoc2a* | -4.6927 | 4.346E-08 |
| *Zc3h12a* | -4.69264 | 6.964E-06 |
| *Pax2* | -4.69196 | 1.141E-06 |
| *Npas2* | -4.68344 | 9.774E-06 |
| *Hoxb8* | -4.6825 | 3.573E-07 |
| *Zswim8* | -4.68054 | 4.964E-15 |
| *Nr6a1* | -4.67896 | 4.296E-06 |
| *Myh9* | -4.67583 | 4.546E-15 |
| *Fut9* | -4.67366 | 1.675E-13 |
| *Erbin* | -4.66984 | 2.946E-13 |
| *Zkscan8* | -4.66881 | 1.041E-16 |
| *Zrsr1* | -4.66847 | 8.862E-13 |
| *Mid2* | -4.667 | 1.077E-11 |
| *Msl2* | -4.66546 | 4.461E-14 |
| *Zbtb10* | -4.6653 | 1.222E-11 |
| *Daam2* | -4.66442 | 4.048E-09 |
| *Vcl* | -4.66349 | 2.487E-14 |
| *Ryr3* | -4.66255 | 9.077E-09 |
| *Lix1l* | -4.65975 | 5.746E-12 |
| *Hoxd4* | -4.6569 | 1.475E-08 |
| *Scrib* | -4.65394 | 8.970E-10 |
| *Ranbp2* | -4.6504 | 2.573E-16 |
| *Ireb2* | -4.65024 | 1.157E-14 |
| *Ralgps1* | -4.64588 | 1.822E-09 |
| *Phldb3* | -4.64122 | 9.416E-06 |
| *Rps6ka3* | -4.63855 | 8.257E-15 |
| *Picalm* | -4.63644 | 7.807E-12 |
| *Epm2a* | -4.63245 | 6.022E-07 |
| *Zfp512b* | -4.62683 | 2.998E-12 |
| *Trim9* | -4.62643 | 5.676E-07 |
| *Rfx7* | -4.62627 | 6.288E-15 |
| *Gpbp1l1* | -4.62179 | 2.413E-13 |
| *Cdc42bpa* | -4.62096 | 1.993E-14 |
| *Tcf20* | -4.62086 | 2.958E-13 |
| *Kmt2d* | -4.61679 | 2.810E-12 |
| *Klhdc10* | -4.61449 | 2.427E-14 |
| *Mybl1* | -4.61435 | 2.667E-05 |
| *Lmtk2* | -4.61158 | 1.455E-10 |
| *Nrbp2* | -4.61154 | 6.599E-11 |
| *2700081O15Rik* | -4.60827 | 1.648E-09 |
| *Ano4* | -4.60425 | 4.442E-09 |
| *C1galt1* | -4.60335 | 1.730E-12 |
| *Unc13b* | -4.60164 | 7.335E-09 |
| *Zfp526* | -4.59897 | 2.956E-06 |
| *Hectd4* | -4.59876 | 2.023E-10 |
| *Plcg1* | -4.5979 | 6.472E-13 |
| *Nup155* | -4.59772 | 3.834E-12 |
| *Slc25a37* | -4.59538 | 9.364E-12 |
| *Anpep* | -4.58912 | 1.263E-12 |
| *Tnks* | -4.58558 | 1.213E-14 |
| *Mcam* | -4.58488 | 7.445E-11 |
| *Etl4* | -4.58371 | 4.241E-12 |
| *Svep1* | -4.58185 | 3.308E-09 |
| *Zbtb18* | -4.57753 | 1.271E-10 |
| *Klf8* | -4.57724 | 8.937E-06 |
| *Setd7* | -4.57704 | 2.960E-16 |
| *Erbb3* | -4.57636 | 6.437E-13 |
| *Lrba* | -4.57539 | 7.935E-18 |
| *Fam120c* | -4.57522 | 6.621E-09 |
| *Rbm26* | -4.57484 | 1.639E-14 |
| *Pcyt1b* | -4.57008 | 7.451E-10 |
| *Ano8* | -4.56676 | 1.560E-05 |
| *Xntrpc* | -4.56284 | 2.436E-06 |
| *D430041D05Rik* | -4.56207 | 1.093E-07 |
| *Rnf213* | -4.56076 | 4.256E-12 |
| *Ehbp1* | -4.55894 | 1.117E-11 |
| *Taf4* | -4.5573 | 6.340E-10 |
| *Nap1l5* | -4.55575 | 7.584E-04 |
| *Mta1* | -4.55568 | 2.024E-11 |
| *Usf3* | -4.55207 | 1.430E-08 |
| *Senp3* | -4.55101 | 2.395E-10 |
| *Setd1b* | -4.54902 | 2.297E-13 |
| *Sav1* | -4.54663 | 3.467E-09 |
| *Adgb* | -4.54633 | 6.602E-13 |
| *Pik3ca* | -4.54554 | 1.895E-15 |
| *Usp9x* | -4.54515 | 1.369E-15 |
| *Pde5a* | -4.545 | 1.326E-15 |
| *Enc1* | -4.54101 | 3.908E-06 |
| *Cdk13* | -4.54007 | 1.336E-12 |
| *Greb1* | -4.53844 | 2.234E-06 |
| *Scaf1* | -4.53805 | 2.302E-11 |
| *Trim24* | -4.53785 | 6.458E-14 |
| *Notch3* | -4.53728 | 6.795E-10 |
| *Thbs1* | -4.53646 | 3.934E-10 |
| *Ankrd28* | -4.53309 | 2.192E-11 |
| *Jrkl* | -4.53279 | 6.474E-08 |
| *Spred2* | -4.52601 | 1.865E-08 |
| *Gm43951* | -4.52547 | 1.464E-05 |
| *Wipf3* | -4.52523 | 6.260E-06 |
| *Naip5* | -4.52127 | 1.223E-11 |
| *Ccdc9* | -4.52103 | 1.015E-07 |
| *Perm1* | -4.51634 | 1.969E-06 |
| *Setd2* | -4.51363 | 2.598E-12 |
| *Trip12* | -4.50973 | 2.176E-13 |
| *Sptan1* | -4.50798 | 9.898E-12 |
| *Ubxn7* | -4.50725 | 9.320E-18 |
| *Phf12* | -4.50704 | 2.186E-13 |
| *Cyp1b1* | -4.50345 | 5.230E-12 |
| *Lats1* | -4.50011 | 1.567E-14 |
| *Ccdc141* | -4.49976 | 2.261E-10 |
| *Padi1* | -4.49885 | 1.214E-10 |
| *D630003M21Rik* | -4.49849 | 8.485E-06 |
| *Jph4* | -4.49842 | 2.342E-06 |
| *Sema4c* | -4.49429 | 3.806E-08 |
| *Map4k2* | -4.49374 | 2.312E-08 |
| *BC037034* | -4.49322 | 2.532E-08 |
| *Cdk8* | -4.49309 | 1.177E-10 |
| *Sos1* | -4.4902 | 4.455E-11 |
| *Vps13c* | -4.48909 | 9.256E-15 |
| *Kcnma1* | -4.48699 | 5.269E-04 |
| *Dach1* | -4.4862 | 6.426E-08 |
| *Trim33* | -4.48408 | 1.032E-13 |
| *Zc3h7a* | -4.47756 | 1.736E-07 |
| *Mapk8* | -4.47711 | 2.465E-11 |
| *Adcy5* | -4.47674 | 8.715E-12 |
| *Clcn6* | -4.47516 | 1.208E-06 |
| *Arhgap33* | -4.46899 | 7.683E-05 |
| *Smg1* | -4.46885 | 2.831E-18 |
| *Rapgefl1* | -4.46849 | 2.422E-07 |
| *Mtcl1* | -4.4677 | 6.358E-04 |
| *Epn1* | -4.46631 | 7.911E-13 |
| *Lama5* | -4.46431 | 1.362E-10 |
| *Gls* | -4.46324 | 6.501E-09 |
| *Shank3* | -4.46151 | 1.598E-06 |
| *Acsf2* | -4.45605 | 3.608E-07 |
| *Cacnb4* | -4.45604 | 2.735E-04 |
| *Plekhh1* | -4.45507 | 1.918E-09 |
| *Cplane1* | -4.44476 | 3.301E-10 |
| *Herc3* | -4.44457 | 3.152E-08 |
| *Hoxb3* | -4.4444 | 4.680E-15 |
| *Acpp* | -4.44413 | 6.715E-12 |
| *Ubr3* | -4.44386 | 3.008E-15 |
| *Ctnnd1* | -4.44341 | 6.867E-12 |
| *Zfp760* | -4.43927 | 2.665E-09 |
| *Ahdc1* | -4.4362 | 5.728E-05 |
| *Med12* | -4.43289 | 3.379E-13 |
| *Fbrs* | -4.43064 | 3.129E-10 |
| *Tecpr2* | -4.43001 | 1.831E-10 |
| *Nfat5* | -4.4273 | 3.989E-16 |
| *Gm7173* | -4.42702 | 5.291E-09 |
| *Npr2* | -4.42657 | 8.710E-09 |
| *Frem2* | -4.42605 | 1.224E-06 |
| *Myof* | -4.42376 | 1.639E-11 |
| *Slc12a2* | -4.42275 | 4.106E-11 |
| *Ddhd1* | -4.41276 | 5.049E-06 |
| *Dagla* | -4.41232 | 4.256E-07 |
| *Trim41* | -4.40947 | 3.827E-10 |
| *Dnah5* | -4.40932 | 7.959E-11 |
| *Ipo7* | -4.4085 | 2.665E-13 |
| *Ppp4r4* | -4.40836 | 2.722E-05 |
| *Gan* | -4.4071 | 1.547E-16 |
| *Agrn* | -4.40664 | 8.575E-13 |
| *Slc20a1* | -4.40346 | 1.846E-09 |
| *Plekha6* | -4.39789 | 1.062E-07 |
| *Zkscan2* | -4.39706 | 2.837E-07 |
| *Bmpr2* | -4.39084 | 5.831E-13 |
| *Adgra2* | -4.39022 | 1.998E-07 |
| *Slc12a5* | -4.38848 | 4.114E-05 |
| *Zfp280d* | -4.38395 | 1.449E-08 |
| *Col11a1* | -4.38319 | 4.547E-06 |
| *Samd1* | -4.37991 | 7.446E-06 |
| *Pga5* | -4.37931 | 5.024E-04 |
| *Calcrl* | -4.37495 | 4.980E-10 |
| *Zkscan16* | -4.37244 | 3.227E-05 |
| *Ptch1* | -4.37099 | 6.146E-14 |
| *Zmym6* | -4.37015 | 3.378E-13 |
| *Apcdd1* | -4.36966 | 2.727E-10 |
| *Zfp318* | -4.36795 | 1.422E-08 |
| *Pi4ka* | -4.36705 | 3.775E-09 |
| *Mdn1* | -4.3657 | 2.430E-13 |
| *Prcc* | -4.36426 | 3.691E-09 |
| *Dennd4b* | -4.36383 | 5.928E-06 |
| *Huwe1* | -4.36377 | 3.566E-15 |
| *Cdk19* | -4.36364 | 2.656E-11 |
| *Cep170b* | -4.36007 | 1.177E-05 |
| *Zfp369* | -4.35608 | 1.868E-09 |
| *2900026A02Rik* | -4.3546 | 4.265E-09 |
| *Sun2* | -4.35257 | 4.165E-08 |
| *Fam13b* | -4.35247 | 8.885E-10 |
| *Osbp* | -4.35021 | 1.288E-10 |
| *Amot* | -4.34834 | 9.784E-12 |
| *Tbc1d9* | -4.34827 | 4.080E-06 |
| *Serinc5* | -4.34747 | 6.391E-13 |
| *Kmt2c* | -4.34741 | 8.305E-13 |
| *Vamp1* | -4.34432 | 8.524E-10 |
| *Cd46* | -4.34401 | 1.367E-07 |
| *Lnpep* | -4.34061 | 1.994E-11 |
| *Chd8* | -4.34017 | 6.294E-12 |
| *Socs4* | -4.33965 | 2.433E-10 |
| *Fer1l5* | -4.33867 | 4.604E-06 |
| *Cbl* | -4.33774 | 1.415E-11 |
| *Nbeal1* | -4.33521 | 1.223E-11 |
| *Atp2b4* | -4.3337 | 1.304E-13 |
| *Rbm33* | -4.33031 | 2.079E-16 |
| *Spag9* | -4.32913 | 2.720E-12 |
| *Epc2* | -4.32886 | 2.329E-10 |
| *D930048N14Rik* | -4.32653 | 2.601E-08 |
| *Dnah7b* | -4.32349 | 7.882E-12 |
| *Hnrnpa3* | -4.32282 | 3.423E-11 |
| *Fn1* | -4.3182 | 1.286E-07 |
| *Cnot1* | -4.31807 | 1.060E-08 |
| *Atxn7l3* | -4.31736 | 9.221E-10 |
| *Dhx33* | -4.31576 | 1.783E-09 |
| *Sox12* | -4.31512 | 1.366E-09 |
| *Cdh5* | -4.31236 | 6.537E-05 |
| *Rgs22* | -4.31231 | 8.435E-12 |
| *Ppard* | -4.3116 | 3.332E-05 |
| *Zfp275* | -4.30932 | 7.751E-10 |
| *Fam135a* | -4.30862 | 2.613E-13 |
| *Pkhd1l1* | -4.30524 | 4.709E-14 |
| *Slx4* | -4.30281 | 2.295E-05 |
| *Fnbp1* | -4.30243 | 1.095E-09 |
| *Bcorl1* | -4.30215 | 5.170E-06 |
| *Dio2* | -4.30166 | 3.486E-05 |
| *Synpo2* | -4.29995 | 2.614E-07 |
| *Tiam2* | -4.29955 | 5.330E-10 |
| *Lmbrd2* | -4.29648 | 1.338E-12 |
| *Inafm2* | -4.29565 | 8.588E-06 |
| *Wnk2* | -4.29435 | 1.846E-07 |
| *Macf1* | -4.29414 | 7.776E-15 |
| *Ern1* | -4.29391 | 7.089E-17 |
| *Tnks1bp1* | -4.29333 | 1.126E-13 |
| *Fam120a* | -4.29306 | 3.473E-11 |
| *Chd3* | -4.29181 | 1.039E-08 |
| *Xk* | -4.29157 | 7.909E-07 |
| *Plce1* | -4.29133 | 1.437E-12 |
| *Zbtb33* | -4.28956 | 6.181E-06 |
| *Tek* | -4.28918 | 6.922E-07 |
| *Nf1* | -4.28891 | 1.061E-13 |
| *Usp32* | -4.28622 | 7.313E-12 |
| *Cap2* | -4.28517 | 7.092E-11 |
| *Flna* | -4.28431 | 1.678E-06 |
| *Il4* | -4.28254 | 1.323E-04 |
| *Dnah12* | -4.28088 | 2.323E-11 |
| *Sfpq* | -4.27875 | 4.013E-08 |
| *Stard9* | -4.27718 | 4.848E-10 |
| *Fam89a* | -4.27569 | 9.204E-04 |
| *Usp54* | -4.27332 | 1.652E-10 |
| *Acin1* | -4.27183 | 1.450E-08 |
| *Ppp1r12b* | -4.2714 | 9.521E-12 |
| *Ncapd3* | -4.26853 | 5.287E-09 |
| *Smarcc2* | -4.26646 | 7.670E-12 |
| *Dnhd1* | -4.26644 | 3.824E-09 |
| *Gm42715* | -4.26473 | 8.429E-10 |
| *Kif5c* | -4.26398 | 4.030E-14 |
| *Enah* | -4.26156 | 5.708E-11 |
| *Shank2* | -4.25719 | 1.166E-07 |
| *Nek10* | -4.25648 | 1.025E-09 |
| *Heca* | -4.2538 | 3.840E-09 |
| *N4bp2* | -4.25281 | 8.823E-13 |
| *Rc3h1* | -4.25236 | 1.980E-11 |
| *Gimap8* | -4.25175 | 3.171E-04 |
| *Zfhx2* | -4.25068 | 6.155E-08 |
| *AI429214* | -4.24557 | 6.932E-06 |
| *Gm20662* | -4.24452 | 2.018E-05 |
| *Alcam* | -4.24352 | 1.175E-08 |
| *Suz12* | -4.2405 | 5.236E-10 |
| *Col5a3* | -4.2389 | 1.323E-06 |
| *Zfp26* | -4.23676 | 4.031E-10 |
| *Chm* | -4.23458 | 1.923E-10 |
| *Zfp626* | -4.23262 | 2.583E-06 |
| *Sytl4* | -4.23215 | 1.118E-06 |
| *D430019H16Rik* | -4.2318 | 5.027E-06 |
| *Wdr90* | -4.23167 | 1.274E-07 |
| *Acacb* | -4.22961 | 7.445E-07 |
| *Col15a1* | -4.22684 | 5.234E-11 |
| *Zfp462* | -4.22106 | 3.881E-11 |
| *Akt3* | -4.21782 | 9.256E-15 |
| *Tmem131* | -4.21427 | 7.313E-12 |
| *Rpia* | -4.21207 | 4.752E-05 |
| *Pfkfb2* | -4.21184 | 1.639E-08 |
| *Psme4* | -4.21172 | 9.402E-13 |
| *Cacnb2* | -4.21108 | 3.099E-12 |
| *Arih1* | -4.2109 | 1.359E-13 |
| *Heatr5b* | -4.2094 | 2.437E-08 |
| *Kdm7a* | -4.20711 | 1.865E-07 |
| *Pan3* | -4.20482 | 7.589E-12 |
| *Syne2* | -4.19831 | 3.826E-11 |
| *Bche* | -4.19771 | 1.736E-11 |
| *Patl1* | -4.19755 | 4.118E-09 |
| *Msi2* | -4.19455 | 2.402E-13 |
| *Arel1* | -4.19454 | 7.804E-10 |
| *Slc8a1* | -4.19443 | 7.241E-14 |
| *Ttc21a* | -4.19334 | 1.884E-12 |
| *Lhcgr* | -4.193 | 3.470E-05 |
| *Zgrf1* | -4.19233 | 6.871E-04 |
| *Mier3* | -4.19118 | 1.646E-10 |
| *Cpsf7* | -4.19075 | 3.155E-12 |
| *Nfatc4* | -4.19042 | 1.146E-07 |
| *Ccnk* | -4.18766 | 5.574E-06 |
| *Bag6* | -4.18587 | 3.725E-09 |
| *D430042O09Rik* | -4.18578 | 7.789E-10 |
| *Dnajb5* | -4.18511 | 1.374E-06 |
| *Cdc42bpb* | -4.1849 | 3.663E-12 |
| *Gm6793* | -4.18266 | 1.629E-06 |
| *Hspg2* | -4.18248 | 7.237E-10 |
| *Tspyl2* | -4.17978 | 8.290E-09 |
| *Soga1* | -4.17901 | 2.703E-07 |
| *Ncor2* | -4.17843 | 3.102E-07 |
| *Coro6* | -4.17716 | 1.685E-04 |
| *Cped1* | -4.17704 | 4.062E-10 |
| *Pi15* | -4.17684 | 1.380E-08 |
| *Trp53bp1* | -4.17535 | 1.472E-11 |
| *Hs6st1* | -4.17482 | 2.591E-12 |
| *Zfp169* | -4.1743 | 3.537E-04 |
| *Psd3* | -4.1676 | 2.613E-13 |
| *Pde7a* | -4.16684 | 1.453E-10 |
| *Als2cl* | -4.16553 | 1.414E-10 |
| *Cacna2d1* | -4.16526 | 4.267E-15 |
| *Ccdc116* | -4.16437 | 5.100E-04 |
| *Dock7* | -4.15942 | 4.256E-12 |
| *Pcmtd1* | -4.15234 | 1.567E-09 |
| *Adamts9* | -4.15192 | 2.808E-07 |
| *Uhmk1* | -4.15167 | 7.354E-11 |
| *Zcchc14* | -4.15097 | 7.621E-08 |
| *Rnf24* | -4.15065 | 1.465E-07 |
| *Dtx3* | -4.14732 | 1.486E-10 |
| *Foxj1* | -4.14619 | 4.878E-11 |
| *Tmem245* | -4.14412 | 3.725E-09 |
| *Cpd* | -4.14332 | 9.939E-08 |
| *Kif27* | -4.14213 | 5.816E-08 |
| *Fnip1* | -4.14167 | 4.825E-11 |
| *Smoc1* | -4.14109 | 3.861E-05 |
| *Vit* | -4.14029 | 3.621E-09 |
| *Cacna1h* | -4.13956 | 3.237E-12 |
| *Tbc1d16* | -4.13944 | 2.919E-08 |
| *Neurl4* | -4.13862 | 3.155E-08 |
| *Aox1* | -4.13829 | 2.022E-05 |
| *Bmpr1b* | -4.13817 | 3.324E-11 |
| *Lrch2* | -4.13587 | 1.058E-05 |
| *Sesn3* | -4.13555 | 1.551E-04 |
| *Abcc9* | -4.13496 | 1.358E-11 |
| *S100pbp* | -4.13459 | 1.010E-10 |
| *Grk3* | -4.13177 | 4.178E-11 |
| *Cmtm4* | -4.13105 | 8.154E-10 |
| *Tmed8* | -4.12872 | 6.020E-08 |
| *AW549877* | -4.12784 | 1.177E-10 |
| *Sp4* | -4.1277 | 1.085E-06 |
| *Strn* | -4.12685 | 7.598E-10 |
| *Pdzd7* | -4.12488 | 3.518E-05 |
| *Usp34* | -4.12445 | 2.733E-10 |
| *Tspan2* | -4.12434 | 1.185E-10 |
| *Cdcp1* | -4.12344 | 1.392E-13 |
| *Akap1* | -4.12259 | 6.003E-10 |
| *Smc5* | -4.12257 | 2.081E-08 |
| *Tra2a* | -4.12152 | 8.145E-09 |
| *Ptch2* | -4.11969 | 8.890E-05 |
| *Prrg1* | -4.11944 | 1.794E-08 |
| *Abhd2* | -4.11727 | 7.604E-11 |
| *Ccdc9b* | -4.11668 | 2.144E-07 |
| *Map3k1* | -4.11609 | 2.091E-08 |
| *Fhod1* | -4.11541 | 1.761E-05 |
| *Ecpas* | -4.11509 | 4.816E-11 |
| *Zer1* | -4.11481 | 9.682E-08 |
| *Samd9l* | -4.11336 | 1.621E-10 |
| *Ago1* | -4.10852 | 3.794E-11 |
| *Cep350* | -4.10712 | 3.831E-13 |
| *Cfap46* | -4.10467 | 1.667E-11 |
| *Esyt2* | -4.10305 | 1.971E-10 |
| *Coro2a* | -4.09945 | 3.250E-10 |
| *Pou6f1* | -4.09788 | 1.648E-10 |
| *Dop1a* | -4.09688 | 3.730E-09 |
| *Zmym2* | -4.09641 | 6.101E-13 |
| *Kif13b* | -4.09625 | 1.321E-16 |
| *Tbc1d24* | -4.09408 | 9.754E-05 |
| *Dlg1* | -4.09342 | 2.140E-11 |
| *Hnrnph1* | -4.09311 | 2.516E-09 |
| *Col4a6* | -4.09293 | 1.222E-11 |
| *Slc12a6* | -4.09234 | 7.807E-11 |
| *Kdm3a* | -4.09185 | 3.330E-09 |
| *Map3k7cl* | -4.0911 | 5.273E-04 |
| *Atg2b* | -4.08913 | 3.679E-06 |
| *Smg7* | -4.08908 | 5.713E-08 |
| *Cblb* | -4.08693 | 3.779E-07 |
| *Zfp827* | -4.08497 | 1.091E-05 |
| *Acsl4* | -4.08487 | 2.558E-08 |
| *Gcnt1* | -4.08378 | 4.387E-10 |
| *Plxnb1* | -4.0837 | 8.841E-11 |
| *Irs1* | -4.0832 | 2.510E-04 |
| *Rnf38* | -4.08095 | 4.260E-12 |
| *Gk5* | -4.07849 | 5.666E-05 |
| *Atp1a2* | -4.07825 | 6.476E-08 |
| *Mbd6* | -4.07664 | 3.688E-08 |
| *Zfp426* | -4.07544 | 2.641E-07 |
| *Acvr2a* | -4.07542 | 2.695E-09 |
| *B3galt5* | -4.07435 | 1.613E-10 |
| *Nuak1* | -4.07211 | 2.709E-06 |
| *Sgpp2* | -4.07132 | 2.419E-04 |
| *Gpr155* | -4.06923 | 1.773E-05 |
| *Atn1* | -4.06863 | 3.444E-11 |
| *Dsp* | -4.06776 | 2.541E-11 |
| *Pde4dip* | -4.06685 | 1.229E-11 |
| *Lepr* | -4.06466 | 4.536E-09 |
| *Ttc41* | -4.06439 | 3.591E-05 |
| *Dhx57* | -4.06399 | 6.710E-09 |
| *Camsap2* | -4.06237 | 1.723E-08 |
| *Ptbp2* | -4.06198 | 3.453E-11 |
| *Zfp619* | -4.06167 | 1.267E-04 |
| *Dnm1* | -4.06107 | 5.676E-05 |
| *Ahcyl1* | -4.05854 | 1.343E-08 |
| *Cdk5r1* | -4.05732 | 1.981E-05 |
| *Ptgfrn* | -4.05597 | 7.797E-11 |
| *Nbr1* | -4.05552 | 2.980E-07 |
| *Btaf1* | -4.05533 | 7.841E-11 |
| *Hnrnpul1* | -4.05464 | 2.767E-11 |
| *Kif3c* | -4.05272 | 7.726E-06 |
| *Jag1* | -4.05205 | 4.331E-11 |
| *Ccdc162* | -4.04936 | 3.882E-12 |
| *Atp8b1* | -4.04835 | 5.096E-11 |
| *Clk2* | -4.04737 | 2.294E-10 |
| *Pds5b* | -4.0465 | 2.698E-12 |
| *Hist2h2be* | -4.04466 | 4.328E-07 |
| *Epb41l1* | -4.04402 | 6.521E-06 |
| *Zc3h3* | -4.04396 | 7.250E-05 |
| *Pbx1* | -4.04319 | 4.902E-10 |
| *Setbp1* | -4.0427 | 3.308E-09 |
| *Gmeb2* | -4.04108 | 2.813E-08 |
| *Paxbp1* | -4.03261 | 3.674E-05 |
| *Ksr1* | -4.03163 | 4.649E-06 |
| *Zfp646* | -4.03085 | 6.410E-08 |
| *Tead3* | -4.03043 | 1.793E-07 |
| *Zfp654* | -4.03003 | 3.255E-08 |
| *Zfp979* | -4.02987 | 2.517E-04 |
| *Nsd2* | -4.02785 | 7.663E-06 |
| *Nacc1* | -4.02773 | 5.793E-11 |
| *Srsf1* | -4.0265 | 2.377E-09 |
| *Ankfn1* | -4.02415 | 2.079E-05 |
| *Dyrk2* | -4.02339 | 5.933E-10 |
| *Rhobtb2* | -4.02147 | 7.335E-09 |
| *Fgf11* | -4.02033 | 1.765E-05 |
| *Usp7* | -4.01931 | 1.940E-09 |
| *Brpf3* | -4.01847 | 1.898E-06 |
| *Zfp729a* | -4.01823 | 8.157E-09 |
| *Htt* | -4.01604 | 1.508E-10 |
| *Kcna2* | -4.01563 | 5.634E-11 |
| *Plagl1* | -4.01559 | 2.147E-09 |
| *Cenpj* | -4.01308 | 3.865E-04 |
| *Prpf8* | -4.01271 | 6.288E-09 |
| *Ino80* | -4.01155 | 4.592E-09 |
| *Map3k19* | -4.011 | 3.359E-07 |
| *Wdr59* | -4.0105 | 8.072E-11 |
| *Vapb* | -4.00964 | 1.318E-08 |
| *Tab3* | -4.0072 | 8.450E-12 |
| *Zfp217* | -4.00552 | 7.208E-08 |
| *Ddx6* | -4.00453 | 2.735E-10 |
| *Ttll3* | -4.00212 | 5.693E-12 |
| *Efnb2* | -3.99788 | 1.238E-09 |
| *Gm44502* | -3.99771 | 8.566E-04 |
| *Tab2* | -3.99636 | 7.217E-11 |
| *Arfgef2* | -3.99596 | 9.158E-09 |
| *Tbc1d13* | -3.994 | 1.178E-09 |
| *Tmtc3* | -3.99368 | 1.711E-11 |
| *A2ml1* | -3.99248 | 2.296E-05 |
| *Bahd1* | -3.99116 | 4.283E-06 |
| *Gria1* | -3.98844 | 1.029E-05 |
| *Slc36a1* | -3.98821 | 3.438E-09 |
| *Ptpn14* | -3.98732 | 2.732E-14 |
| *Krt13* | -3.98653 | 9.125E-04 |
| *Zc3h4* | -3.98624 | 5.354E-10 |
| *Fmnl2* | -3.98455 | 5.857E-10 |
| *Pank3* | -3.98248 | 1.541E-09 |
| *Dpp8* | -3.98013 | 2.140E-11 |
| *1700029I15Rik* | -3.97902 | 7.434E-04 |
| *Rbm15b* | -3.97851 | 2.849E-08 |
| *Atp8b2* | -3.97588 | 9.309E-08 |
| *Dclk2* | -3.9727 | 5.292E-06 |
| *Rb1* | -3.96735 | 4.512E-10 |
| *Prob1* | -3.96722 | 6.390E-08 |
| *Crybg3* | -3.9661 | 1.060E-13 |
| *Mab21l3* | -3.96567 | 6.205E-05 |
| *Scai* | -3.96542 | 3.460E-12 |
| *Slc25a36* | -3.96414 | 2.824E-08 |
| *Ccdc39* | -3.96248 | 4.312E-08 |
| *Mical2* | -3.96187 | 3.743E-15 |
| *Dido1* | -3.95903 | 2.249E-11 |
| *Fgd5* | -3.95864 | 1.660E-04 |
| *Tada2b* | -3.95807 | 1.006E-05 |
| *Dot1l* | -3.95791 | 6.373E-11 |
| *Itgb4* | -3.95321 | 4.843E-08 |
| *Tshz3* | -3.95086 | 9.633E-12 |
| *Plekhg6* | -3.94643 | 1.427E-04 |
| *Heatr1* | -3.94513 | 4.681E-08 |
| *Qtrt2* | -3.94497 | 1.914E-05 |
| *Kalrn* | -3.94349 | 1.646E-10 |
| *Uprt* | -3.94237 | 9.296E-05 |
| *Tfrc* | -3.94195 | 7.005E-06 |
| *Pram1* | -3.94164 | 5.191E-04 |
| *Magi3* | -3.94041 | 8.844E-12 |
| *Kat2b* | -3.94027 | 2.483E-11 |
| *Far1* | -3.93984 | 5.002E-09 |
| *Zcchc2* | -3.93834 | 4.728E-08 |
| *Atp11a* | -3.93775 | 2.957E-06 |
| *Usp49* | -3.93547 | 4.363E-05 |
| *Edrf1* | -3.93298 | 8.322E-06 |
| *Pdgfra* | -3.93294 | 2.020E-14 |
| *Cdc73* | -3.93237 | 3.127E-10 |
| *Tnrc18* | -3.93224 | 2.479E-12 |
| *Zhx2* | -3.92994 | 4.760E-05 |
| *Chd4* | -3.92983 | 5.840E-09 |
| *Zfp608* | -3.92884 | 4.731E-10 |
| *Ccp110* | -3.92836 | 7.598E-10 |
| *Ptger1* | -3.92538 | 2.971E-05 |
| *Dnajc16* | -3.92491 | 4.088E-08 |
| *Eps15l1* | -3.92348 | 3.835E-09 |
| *Psd* | -3.92174 | 1.242E-06 |
| *Atxn1l* | -3.91947 | 8.098E-05 |
| *Myo9b* | -3.91739 | 4.821E-08 |
| *Ugt1a2* | -3.91481 | 1.366E-05 |
| *Pabpc4* | -3.91361 | 6.217E-09 |
| *Dazap1* | -3.91305 | 2.677E-06 |
| *Morc2a* | -3.9124 | 6.128E-10 |
| *Xpo7* | -3.91145 | 8.660E-06 |
| *Baz2a* | -3.91001 | 2.723E-14 |
| *Mpst* | -3.90946 | 3.541E-06 |
| *Ppef2* | -3.90931 | 8.893E-05 |
| *Zfp458* | -3.90632 | 4.259E-05 |
| *Fnip2* | -3.90618 | 2.233E-07 |
| *Atp11b* | -3.90541 | 5.515E-11 |
| *Chic1* | -3.90467 | 5.436E-07 |
| *Npr3* | -3.90458 | 3.607E-06 |
| *Col4a3* | -3.90392 | 8.708E-07 |
| *Zbtb41* | -3.90358 | 4.585E-10 |
| *Mob1b* | -3.90208 | 7.374E-12 |
| *Polr2a* | -3.89985 | 1.679E-11 |
| *Urb2* | -3.89977 | 7.893E-06 |
| *Fastkd1* | -3.89969 | 1.709E-04 |
| *Mgarp* | -3.89747 | 8.041E-04 |
| *Rab11fip4* | -3.89655 | 1.301E-10 |
| *Armcx4* | -3.89579 | 2.311E-06 |
| *Sirt1* | -3.89519 | 5.449E-10 |
| *Fam199x* | -3.89372 | 2.986E-06 |
| *Gramd1b* | -3.89188 | 1.094E-08 |
| *Tlr4* | -3.89152 | 1.379E-08 |
| *Plch1* | -3.89143 | 4.119E-09 |
| *Mapk8ip3* | -3.89067 | 4.281E-07 |
| *Trpm6* | -3.89052 | 5.868E-06 |
| *Psd4* | -3.89021 | 1.221E-04 |
| *Vps13b* | -3.88823 | 5.261E-11 |
| *Trrap* | -3.88819 | 3.127E-12 |
| *E2f3* | -3.88741 | 8.879E-05 |
| *Ebf4* | -3.88734 | 2.295E-05 |
| *Peak1* | -3.88433 | 7.608E-14 |
| *Dzank1* | -3.88371 | 4.256E-12 |
| *Bclaf1* | -3.88314 | 1.495E-08 |
| *Acrbp* | -3.88028 | 7.357E-05 |
| *Hnrnpl* | -3.88026 | 1.022E-07 |
| *Col20a1* | -3.88017 | 3.044E-05 |
| *Setdb1* | -3.87744 | 1.079E-10 |
| *Mprip* | -3.87597 | 1.459E-08 |
| *Prpf39* | -3.87569 | 3.925E-10 |
| *Tlk2* | -3.87552 | 6.985E-09 |
| *Zkscan7* | -3.87373 | 9.006E-09 |
| *Abcb7* | -3.87295 | 9.764E-08 |
| *Ttll4* | -3.87282 | 9.470E-08 |
| *Rbm45* | -3.87182 | 1.061E-06 |
| *Tns3* | -3.87171 | 1.126E-09 |
| *Has3* | -3.87162 | 2.488E-05 |
| *Nhs* | -3.87148 | 6.515E-05 |
| *Scn7a* | -3.87054 | 1.114E-07 |
| *Herc1* | -3.87053 | 8.953E-11 |
| *Cep135* | -3.87003 | 5.979E-06 |
| *Uba6* | -3.86947 | 6.866E-07 |
| *Ankrd13b* | -3.86804 | 7.176E-05 |
| *Crocc* | -3.8674 | 2.531E-09 |
| *Utp14b* | -3.86708 | 3.189E-05 |
| *Sptbn2* | -3.86706 | 1.620E-07 |
| *Sptb* | -3.86584 | 8.701E-07 |
| *Zfp953* | -3.86581 | 1.664E-05 |
| *Kat6b* | -3.86442 | 3.402E-12 |
| *Ocrl* | -3.86133 | 1.002E-09 |
| *Abca2* | -3.86036 | 5.501E-09 |
| *Scyl2* | -3.85571 | 9.893E-08 |
| *4932438A13Rik* | -3.85344 | 5.903E-09 |
| *Kcnq4* | -3.85327 | 2.728E-05 |
| *Slc22a3* | -3.8525 | 2.900E-04 |
| *Atp2b2* | -3.85199 | 6.044E-08 |
| *Shroom2* | -3.85167 | 1.727E-06 |
| *Acvr1b* | -3.85138 | 2.737E-06 |
| *Ank2* | -3.85063 | 1.494E-06 |
| *Dnajc13* | -3.85001 | 2.671E-10 |
| *Nedd4* | -3.84976 | 1.250E-08 |
| *Cic* | -3.8497 | 1.108E-09 |
| *Arhgef6* | -3.84841 | 2.870E-04 |
| *Kif1c* | -3.8478 | 7.780E-13 |
| *Sacs* | -3.84775 | 6.578E-10 |
| *Ankrd50* | -3.84711 | 9.418E-09 |
| *Igf2r* | -3.84567 | 3.695E-09 |
| *Csrnp3* | -3.84486 | 1.088E-05 |
| *Inhbb* | -3.84431 | 3.083E-04 |
| *Kansl1* | -3.84372 | 5.361E-09 |
| *Dcbld2* | -3.84296 | 5.710E-10 |
| *Pcnx3* | -3.84295 | 7.223E-10 |
| *Klhl29* | -3.84197 | 2.681E-06 |
| *Hyou1* | -3.84135 | 3.349E-08 |
| *Srgap3* | -3.84121 | 1.785E-07 |
| *Tbcel* | -3.84109 | 4.008E-05 |
| *Clstn2* | -3.83955 | 1.314E-04 |
| *Pds5a* | -3.83924 | 2.648E-10 |
| *mt-Nd5* | -3.83748 | 1.422E-05 |
| *Nol10* | -3.83724 | 3.952E-05 |
| *Ptpn23* | -3.8358 | 4.617E-09 |
| *Eppk1* | -3.83543 | 2.352E-07 |
| *Qser1* | -3.8344 | 1.907E-06 |
| *Zfp598* | -3.83426 | 8.998E-08 |
| *Fignl2* | -3.8329 | 6.317E-05 |
| *Oas2* | -3.83267 | 2.360E-07 |
| *Clic5* | -3.82962 | 1.474E-08 |
| *Hmgxb4* | -3.82833 | 5.655E-05 |
| *Zfp638* | -3.82449 | 1.323E-10 |
| *Prkab2* | -3.82412 | 8.743E-08 |
| *Hsf2* | -3.82231 | 3.298E-08 |
| *Anp32a* | -3.82006 | 7.248E-10 |
| *Haus6* | -3.81961 | 3.100E-05 |
| *Lgals4* | -3.81853 | 6.759E-04 |
| *Adamts19* | -3.81829 | 2.076E-04 |
| *Sec16b* | -3.81597 | 6.286E-05 |
| *Dmxl1* | -3.81527 | 4.870E-10 |
| *Xiap* | -3.81475 | 4.332E-09 |
| *Mamld1* | -3.81463 | 6.376E-07 |
| *Pgr* | -3.81389 | 6.975E-09 |
| *Ano5* | -3.81256 | 1.426E-04 |
| *Wdr82* | -3.81217 | 2.871E-09 |
| *Dennd4c* | -3.81019 | 3.773E-11 |
| *Zfp711* | -3.81007 | 5.270E-04 |
| *Itprid2* | -3.80988 | 2.197E-08 |
| *Kcnt2* | -3.80879 | 3.687E-04 |
| *Taok1* | -3.8087 | 4.522E-09 |
| *Sestd1* | -3.80855 | 1.390E-09 |
| *Anapc1* | -3.80828 | 1.749E-07 |
| *Uhrf1bp1* | -3.80733 | 6.705E-07 |
| *Plp1* | -3.80507 | 8.723E-04 |
| *Map4k4* | -3.80396 | 1.946E-12 |
| *Tmtc2* | -3.80276 | 2.095E-08 |
| *Arhgef5* | -3.80122 | 3.250E-10 |
| *Gtf3c1* | -3.80102 | 8.132E-09 |
| *Zfp516* | -3.79984 | 2.219E-15 |
| *Nin* | -3.79847 | 2.418E-09 |
| *Agap2* | -3.79837 | 2.207E-09 |
| *9930021J03Rik* | -3.79635 | 5.075E-08 |
| *Zfp109* | -3.79558 | 2.584E-04 |
| *Loxl2* | -3.79553 | 2.681E-06 |
| *Ccdc50* | -3.79533 | 4.394E-09 |
| *Thrb* | -3.79485 | 1.046E-10 |
| *Cldn1* | -3.79438 | 1.617E-06 |
| *Ptgs2* | -3.79434 | 5.012E-04 |
| *Kdm5c* | -3.79333 | 1.735E-14 |
| *Mki67* | -3.79292 | 1.400E-04 |
| *Fam193b* | -3.79285 | 2.808E-09 |
| *Ddx3x* | -3.79281 | 7.152E-08 |
| *Ric1* | -3.79174 | 1.537E-10 |
| *Slc38a6* | -3.79093 | 8.172E-07 |
| *Atad5* | -3.79027 | 5.345E-05 |
| *Cpeb2* | -3.78858 | 2.018E-08 |
| *Mbtd1* | -3.78812 | 8.903E-10 |
| *Smarca1* | -3.78775 | 1.617E-06 |
| *Kpnb1* | -3.78689 | 3.306E-08 |
| *Prdm2* | -3.78686 | 4.556E-11 |
| *Dock5* | -3.78646 | 8.204E-09 |
| *Atp13a3* | -3.78616 | 2.865E-12 |
| *Adgrf5* | -3.78377 | 3.781E-07 |
| *Wdr76* | -3.78334 | 2.267E-05 |
| *Tlr3* | -3.78202 | 2.558E-08 |
| *Tmem252* | -3.78184 | 7.529E-08 |
| *Fam214a* | -3.77758 | 4.899E-08 |
| *Taf1* | -3.77636 | 6.196E-10 |
| *Dip2c* | -3.77619 | 2.872E-07 |
| *Appl2* | -3.77411 | 4.911E-06 |
| *Wdfy2* | -3.77397 | 1.448E-07 |
| *Myef2* | -3.77397 | 2.081E-06 |
| *Rnf144b* | -3.77051 | 4.568E-09 |
| *Rbbp6* | -3.76926 | 1.703E-07 |
| *Sowahc* | -3.76708 | 2.667E-05 |
| *BC067074* | -3.76675 | 1.632E-05 |
| *AC115631.1* | -3.76581 | 9.826E-06 |
| *Rnf217* | -3.76235 | 8.399E-10 |
| *Ltn1* | -3.76175 | 1.425E-07 |
| *Arhgef17* | -3.761 | 7.544E-09 |
| *Pi4kb* | -3.76071 | 1.789E-09 |
| *Akap10* | -3.75818 | 5.512E-06 |
| *Ago2* | -3.7579 | 9.901E-09 |
| *Pxn* | -3.75779 | 3.106E-09 |
| *Zmpste24* | -3.75757 | 2.391E-07 |
| *Zbtb37* | -3.75659 | 9.565E-16 |
| *Whrn* | -3.75658 | 1.206E-07 |
| *Zfp759* | -3.75104 | 9.050E-05 |
| *Snn* | -3.75036 | 1.368E-08 |
| *Zwilch* | -3.749 | 4.669E-04 |
| *Camk2n1* | -3.7481 | 6.436E-07 |
| *Dlg5* | -3.74678 | 6.020E-08 |
| *Utp20* | -3.74574 | 1.923E-09 |
| *Diexf* | -3.74531 | 8.186E-04 |
| *Hook1* | -3.74509 | 6.537E-06 |
| *Dip2a* | -3.74477 | 1.151E-06 |
| *Flnc* | -3.74452 | 3.218E-09 |
| *Zfp704* | -3.74395 | 6.374E-09 |
| *Pkd2l2* | -3.74304 | 5.952E-04 |
| *Sf1* | -3.7417 | 5.554E-04 |
| *Simc1* | -3.74166 | 9.602E-05 |
| *Hmga2* | -3.74125 | 2.630E-04 |
| *Gtpbp2* | -3.73853 | 8.671E-08 |
| *Usp35* | -3.73829 | 5.344E-05 |
| *Caskin2* | -3.73795 | 3.117E-08 |
| *F2rl1* | -3.73766 | 9.553E-05 |
| *Clock* | -3.73659 | 1.028E-10 |
| *Trak1* | -3.73559 | 3.509E-09 |
| *Pdpr* | -3.73264 | 9.417E-08 |
| *Nkrf* | -3.7326 | 4.269E-04 |
| *Zc3h7b* | -3.73238 | 1.005E-10 |
| *Ttll2* | -3.7323 | 3.502E-04 |
| *Zbtb7b* | -3.73206 | 1.456E-06 |
| *Tert* | -3.731 | 5.326E-04 |
| *Ssc5d* | -3.73047 | 3.839E-05 |
| *Eogt* | -3.73026 | 2.449E-07 |
| *Fbxl20* | -3.72877 | 4.349E-09 |
| *Itsn1* | -3.72758 | 1.188E-06 |
| *Prpf19* | -3.72397 | 1.425E-07 |
| *Prr12* | -3.72071 | 6.361E-08 |
| *Gm13212* | -3.72048 | 1.248E-06 |
| *Tenm3* | -3.71932 | 5.609E-07 |
| *Zbtb26* | -3.7181 | 8.838E-07 |
| *Erich3* | -3.71808 | 5.933E-10 |
| *Bcl2l2* | -3.71789 | 7.605E-06 |
| *BC005537* | -3.71711 | 9.893E-08 |
| *Mtcp1* | -3.71657 | 9.284E-09 |
| *Plxna2* | -3.71657 | 5.320E-07 |
| *Stx1b* | -3.71541 | 7.919E-05 |
| *Jag2* | -3.71324 | 9.782E-05 |
| *Pik3r3* | -3.71306 | 5.951E-06 |
| *Ice2* | -3.71202 | 3.341E-08 |
| *Gtf2a1* | -3.711 | 2.481E-08 |
| *Rmnd5a* | -3.7107 | 9.993E-08 |
| *Aebp2* | -3.70943 | 1.129E-06 |
| *Nup153* | -3.70804 | 4.392E-09 |
| *Arhgef4* | -3.70677 | 6.345E-08 |
| *Dmtf1* | -3.70671 | 3.000E-10 |
| *Ntn4* | -3.70617 | 2.907E-05 |
| *Dnah9* | -3.70598 | 5.125E-06 |
| *Fam208b* | -3.70593 | 1.299E-13 |
| *Arnt2* | -3.70451 | 4.884E-09 |
| *Tagln* | -3.70079 | 8.000E-07 |
| *Max* | -3.70066 | 4.407E-09 |
| *Arid2* | -3.69954 | 1.544E-09 |
| *Abl1* | -3.69951 | 5.796E-09 |
| *Capn1* | -3.6988 | 6.441E-07 |
| *Pnisr* | -3.69735 | 4.492E-07 |
| *Ulk3* | -3.697 | 3.133E-04 |
| *Lemd3* | -3.6958 | 1.718E-05 |
| *Dock11* | -3.69361 | 8.270E-05 |
| *Pof1b* | -3.69287 | 1.035E-04 |
| *Hps5* | -3.69254 | 5.324E-06 |
| *Itgb6* | -3.69089 | 1.491E-04 |
| *Pdia2* | -3.69003 | 8.192E-04 |
| *Crybg1* | -3.68988 | 1.795E-05 |
| *Usp31* | -3.68972 | 3.262E-05 |
| *Itga9* | -3.68962 | 2.539E-05 |
| *Trim44* | -3.68913 | 1.039E-08 |
| *Retreg3* | -3.6888 | 1.236E-07 |
| *Caprin2* | -3.68515 | 4.802E-10 |
| *Prx* | -3.68489 | 2.906E-04 |
| *Gm12258* | -3.68294 | 5.465E-07 |
| *Med12l* | -3.68294 | 7.602E-05 |
| *Abcc4* | -3.68226 | 9.296E-07 |
| *Rec8* | -3.68204 | 3.631E-05 |
| *Snx25* | -3.68194 | 1.888E-04 |
| *Frmpd2* | -3.6818 | 1.480E-11 |
| *Mosmo* | -3.68153 | 1.862E-08 |
| *Rfx5* | -3.67927 | 2.803E-06 |
| *Dchs1* | -3.67868 | 3.000E-05 |
| *Aftph* | -3.67807 | 2.919E-08 |
| *Rad54l2* | -3.67741 | 1.630E-10 |
| *Cul4b* | -3.67484 | 2.127E-09 |
| *Plag1* | -3.67334 | 1.013E-05 |
| *Stk38l* | -3.67298 | 6.350E-07 |
| *Esrp1* | -3.67254 | 3.028E-07 |
| *Ppp6r1* | -3.67221 | 1.254E-08 |
| *Ptprs* | -3.6719 | 4.133E-07 |
| *Ltbp1* | -3.67161 | 2.910E-09 |
| *Pcf11* | -3.67049 | 1.600E-10 |
| *Zfp874b* | -3.66987 | 5.919E-06 |
| *Ddx21* | -3.66945 | 6.821E-08 |
| *Npat* | -3.6688 | 7.385E-07 |
| *Nfatc2ip* | -3.66827 | 2.580E-04 |
| *Abca9* | -3.66788 | 1.018E-07 |
| *Cep152* | -3.66657 | 3.606E-04 |
| *Foxp4* | -3.66499 | 4.624E-06 |
| *Hdac7* | -3.66455 | 4.614E-07 |
| *Gpr157* | -3.6627 | 2.954E-04 |
| *Fzd6* | -3.66162 | 2.814E-09 |
| *Pafah1b1* | -3.66082 | 2.187E-05 |
| *Nvl* | -3.65932 | 1.479E-06 |
| *Trappc10* | -3.65884 | 9.962E-08 |
| *Zmym4* | -3.65663 | 5.287E-12 |
| *Pde7b* | -3.6562 | 2.032E-07 |
| *Tfcp2l1* | -3.65495 | 1.872E-11 |
| *Blm* | -3.65254 | 4.728E-04 |
| *Ppp1r9b* | -3.65236 | 4.944E-09 |
| *Abca8a* | -3.65176 | 7.426E-05 |
| *Nmur2* | -3.65049 | 6.037E-04 |
| *Lrfn4* | -3.65035 | 1.210E-05 |
| *Slit2* | -3.65019 | 2.261E-11 |
| *Cntnap3* | -3.64983 | 2.319E-04 |
| *Dock6* | -3.64754 | 4.556E-11 |
| *Prdm11* | -3.64599 | 1.366E-09 |
| *Sort1* | -3.64588 | 7.335E-09 |
| *Eif4b* | -3.64548 | 6.155E-08 |
| *Ivns1abp* | -3.64393 | 1.189E-07 |
| *Nufip2* | -3.64348 | 3.208E-08 |
| *Hoxa9* | -3.64317 | 3.676E-08 |
| *Kdm6a* | -3.64297 | 6.077E-10 |
| *Map4k3* | -3.64275 | 2.147E-09 |
| *Hoxa5* | -3.64113 | 9.177E-06 |
| *Col16a1* | -3.63986 | 1.233E-08 |
| *Carm1* | -3.63933 | 5.524E-07 |
| *Ncoa6* | -3.63819 | 5.096E-11 |
| *Malt1* | -3.63811 | 3.518E-04 |
| *Ednra* | -3.63664 | 7.092E-07 |
| *Nptx1* | -3.63624 | 2.481E-04 |
| *Elmod2* | -3.63577 | 4.663E-07 |
| *Rttn* | -3.63545 | 8.241E-05 |
| *Nepro* | -3.63387 | 1.764E-04 |
| *Mex3c* | -3.6314 | 4.663E-09 |
| *Ep300* | -3.63137 | 7.630E-11 |
| *Gm9958* | -3.63092 | 6.045E-04 |
| *N4bp1* | -3.62963 | 5.930E-08 |
| *Slc15a1* | -3.62804 | 1.260E-05 |
| *Capn15* | -3.62713 | 3.562E-05 |
| *Tesk1* | -3.62614 | 9.080E-05 |
| *Zfyve9* | -3.62549 | 7.639E-07 |
| *Ppp4r2* | -3.62466 | 1.386E-08 |
| *Bcl6b* | -3.62357 | 8.718E-04 |
| *Kri1* | -3.62336 | 1.716E-06 |
| *Rassf2* | -3.6222 | 2.779E-09 |
| *Lig4* | -3.62195 | 6.317E-04 |
| *Ccnyl1* | -3.62151 | 2.651E-04 |
| *Fhod3* | -3.62065 | 3.769E-04 |
| *Stag2* | -3.61833 | 1.214E-10 |
| *Col4a5* | -3.61802 | 4.362E-08 |
| *Fbxo11* | -3.61737 | 9.097E-09 |
| *Lpar2* | -3.61682 | 2.968E-04 |
| *Rap2c* | -3.6167 | 1.970E-06 |
| *Cdkl5* | -3.61631 | 5.018E-05 |
| *Lrrk2* | -3.6154 | 2.587E-06 |
| *Sympk* | -3.61521 | 1.585E-06 |
| *Bicral* | -3.61492 | 2.001E-09 |
| *Gm20605* | -3.61331 | 2.148E-04 |
| *Ddx11* | -3.61264 | 4.658E-05 |
| *Cep170* | -3.61239 | 7.293E-05 |
| *Atf7* | -3.61202 | 8.715E-12 |
| *Gpr137c* | -3.61197 | 7.040E-08 |
| *Col5a2* | -3.61188 | 8.999E-08 |
| *Phka1* | -3.61164 | 8.231E-07 |
| *Col12a1* | -3.61111 | 1.283E-17 |
| *Nbeal2* | -3.61014 | 1.702E-05 |
| *Map3k13* | -3.60993 | 2.335E-04 |
| *Rbl2* | -3.60939 | 3.865E-08 |
| *BC048403* | -3.60874 | 5.031E-04 |
| *Rtel1* | -3.60855 | 2.142E-05 |
| *Ice1* | -3.60827 | 2.620E-08 |
| *Jph2* | -3.60803 | 1.517E-04 |
| *Kcnb1* | -3.60638 | 5.899E-04 |
| *Hipk1* | -3.60535 | 4.841E-08 |
| *Zfp407* | -3.60347 | 1.242E-05 |
| *Nek9* | -3.6024 | 1.566E-08 |
| *Atp11c* | -3.60148 | 4.856E-08 |
| *Cc2d2a* | -3.60083 | 2.546E-08 |
| *Rasef* | -3.60071 | 2.585E-07 |
| *Atp10b* | -3.60007 | 6.741E-08 |
| *Rad21* | -3.59853 | 1.309E-08 |
| *Acss3* | -3.5985 | 5.361E-09 |
| *Adam19* | -3.5973 | 7.396E-07 |
| *Dzip3* | -3.59585 | 4.044E-06 |
| *Pik3c2b* | -3.59569 | 1.891E-07 |
| *Ppip5k2* | -3.59522 | 1.027E-07 |
| *Lyst* | -3.59475 | 1.809E-10 |
| *Midn* | -3.59369 | 3.695E-05 |
| *Fryl* | -3.59193 | 8.993E-09 |
| *Vcan* | -3.58982 | 7.119E-09 |
| *Dpy19l4* | -3.58843 | 2.620E-07 |
| *Grk5* | -3.5853 | 8.589E-04 |
| *Mypop* | -3.58428 | 8.503E-04 |
| *Adgrl3* | -3.58371 | 3.202E-07 |
| *Mnt* | -3.5836 | 1.258E-06 |
| *Tmem56* | -3.5834 | 7.787E-07 |
| *Slc25a23* | -3.5833 | 2.817E-10 |
| *Rsc1a1* | -3.58284 | 3.458E-05 |
| *Suco* | -3.58185 | 1.311E-06 |
| *Glis3* | -3.58056 | 1.114E-07 |
| *Atl3* | -3.57895 | 3.198E-10 |
| *Lrp1* | -3.57862 | 1.444E-04 |
| *Fgfr2* | -3.57387 | 9.221E-08 |
| *Cep295* | -3.57385 | 2.022E-05 |
| *Igdcc4* | -3.57312 | 2.151E-06 |
| *Arhgef12* | -3.57206 | 1.450E-08 |
| *Hoxd3* | -3.57205 | 5.343E-05 |
| *Daam1* | -3.56965 | 7.173E-08 |
| *Arid1a* | -3.5679 | 2.681E-08 |
| *Exoc5* | -3.56733 | 3.655E-04 |
| *Igf1r* | -3.56686 | 1.216E-09 |
| *Cdon* | -3.56551 | 2.764E-08 |
| *Cops2* | -3.56494 | 2.850E-06 |
| *Dnase1* | -3.56414 | 4.775E-04 |
| *Rhoq* | -3.5634 | 2.736E-08 |
| *Tnni3* | -3.56266 | 3.482E-04 |
| *Uso1* | -3.56061 | 2.342E-06 |
| *Sik3* | -3.56044 | 6.389E-09 |
| *Gm20661* | -3.55919 | 6.829E-05 |
| *Map3k5* | -3.5589 | 7.209E-07 |
| *Ogt* | -3.55734 | 1.596E-07 |
| *Dnal1* | -3.55344 | 3.615E-11 |
| *Homer1* | -3.55163 | 6.218E-05 |
| *Pcdhb16* | -3.55156 | 3.112E-04 |
| *Jade2* | -3.55015 | 1.597E-06 |
| *Sall1* | -3.55011 | 1.057E-07 |
| *Six4* | -3.55009 | 4.742E-05 |
| *Zfp317* | -3.54897 | 5.933E-08 |
| *Slc25a30* | -3.54767 | 5.921E-05 |
| *Heatr5a* | -3.54662 | 2.380E-09 |
| *Kbtbd7* | -3.54619 | 6.177E-05 |
| *Peg3* | -3.54561 | 6.726E-08 |
| *Trappc8* | -3.54484 | 1.150E-08 |
| *Ptar1* | -3.54273 | 1.878E-08 |
| *Plch2* | -3.5425 | 3.860E-05 |
| *Shroom4* | -3.54211 | 2.564E-05 |
| *Kpna6* | -3.5421 | 2.077E-08 |
| *Jmy* | -3.54094 | 8.759E-09 |
| *Stard13* | -3.54064 | 3.367E-06 |
| *Prkd3* | -3.53767 | 1.937E-09 |
| *Grik5* | -3.53635 | 1.199E-05 |
| *Rif1* | -3.53496 | 3.046E-09 |
| *Eml6* | -3.53456 | 5.841E-06 |
| *mt-Nd6* | -3.53172 | 9.746E-05 |
| *Lmntd2* | -3.52776 | 3.414E-04 |
| *Usp38* | -3.5256 | 1.117E-06 |
| *Ap1g2* | -3.52548 | 3.697E-05 |
| *Zfp36l2* | -3.5253 | 5.970E-08 |
| *Chst11* | -3.52485 | 1.599E-06 |
| *Sall2* | -3.52402 | 2.847E-06 |
| *Wdr13* | -3.52348 | 8.132E-09 |
| *Dcaf1* | -3.52336 | 1.174E-06 |
| *Gm14226* | -3.52319 | 2.720E-05 |
| *Wdr26* | -3.52314 | 6.670E-07 |
| *Mark1* | -3.52313 | 4.338E-04 |
| *Zmym1* | -3.52195 | 4.783E-05 |
| *Zfp65* | -3.52126 | 3.663E-05 |
| *Ube2q1* | -3.52118 | 4.116E-08 |
| *Usp19* | -3.52093 | 2.076E-07 |
| *Kank2* | -3.51806 | 1.012E-07 |
| *Kidins220* | -3.51726 | 6.581E-07 |
| *Myh10* | -3.51701 | 1.851E-11 |
| *Gpr161* | -3.5149 | 1.766E-04 |
| *Zfp871* | -3.51422 | 1.954E-08 |
| *Sept5* | -3.51342 | 1.091E-06 |
| *Dhx36* | -3.51177 | 1.251E-07 |
| *Dysf* | -3.51154 | 2.542E-04 |
| *Lclat1* | -3.51022 | 2.075E-08 |
| *Senp5* | -3.50945 | 4.568E-07 |
| *Smg5* | -3.50931 | 8.078E-07 |
| *Ap2b1* | -3.50851 | 2.191E-09 |
| *Synj2* | -3.50691 | 6.808E-05 |
| *Ranbp6* | -3.50622 | 1.018E-06 |
| *Nbea* | -3.50516 | 1.447E-10 |
| *Gabpa* | -3.50477 | 6.015E-09 |
| *Alpk1* | -3.50456 | 2.863E-06 |
| *Itga8* | -3.50445 | 3.190E-04 |
| *Bms1* | -3.50326 | 4.561E-08 |
| *Ctif* | -3.5031 | 3.599E-04 |
| *Micall2* | -3.50181 | 8.737E-04 |
| *Yeats2* | -3.50053 | 6.429E-06 |
| *Tnpo2* | -3.50014 | 2.966E-07 |
| *Helz2* | -3.49857 | 1.691E-05 |
| *Elf4* | -3.49815 | 3.314E-06 |
| *Tbkbp1* | -3.49707 | 7.384E-04 |
| *Hipk3* | -3.49576 | 1.139E-06 |
| *Zfp91* | -3.49514 | 8.541E-08 |
| *Arhgap17* | -3.49426 | 1.107E-07 |
| *Lrp12* | -3.49408 | 1.130E-04 |
| *Ddx23* | -3.49402 | 2.557E-14 |
| *Ppp3cb* | -3.49286 | 5.680E-07 |
| *Doc2b* | -3.49229 | 2.383E-04 |
| *Megf8* | -3.49203 | 2.146E-07 |
| *Krba1* | -3.49014 | 8.284E-09 |
| *Rbm6* | -3.48966 | 1.851E-06 |
| *Dync1h1* | -3.48941 | 8.172E-10 |
| *Klhl20* | -3.48879 | 1.539E-04 |
| *Gli2* | -3.4887 | 6.748E-05 |
| *Rgp1* | -3.48814 | 7.424E-05 |
| *Parp4* | -3.48802 | 2.324E-10 |
| *Ambra1* | -3.48651 | 6.420E-08 |
| *Xpr1* | -3.48611 | 5.904E-09 |
| *Zfp81* | -3.48144 | 8.136E-07 |
| *Sox13* | -3.48015 | 5.822E-06 |
| *2610008E11Rik* | -3.47963 | 6.196E-09 |
| *Lama2* | -3.47828 | 4.919E-12 |
| *Mthfr* | -3.47826 | 1.590E-08 |
| *Slc39a10* | -3.47683 | 2.056E-07 |
| *Kdm2a* | -3.47661 | 1.016E-11 |
| *Aff4* | -3.47616 | 1.638E-07 |
| *Akap12* | -3.47573 | 7.041E-08 |
| *Abca4* | -3.47489 | 4.466E-04 |
| *Zbtb40* | -3.47461 | 9.879E-04 |
| *Hgf* | -3.47197 | 1.251E-04 |
| *Otud4* | -3.47118 | 2.152E-08 |
| *Trove2* | -3.47052 | 5.524E-07 |
| *Dennd5a* | -3.47017 | 5.208E-09 |
| *Foxj3* | -3.46973 | 4.338E-07 |
| *Susd1* | -3.46929 | 3.599E-04 |
| *Atrnl1* | -3.46707 | 7.428E-08 |
| *Hook3* | -3.46626 | 2.394E-08 |
| *Mon2* | -3.46452 | 5.599E-06 |
| *Slc34a2* | -3.46376 | 1.417E-06 |
| *Plekhm1* | -3.46209 | 2.460E-05 |
| *Specc1l* | -3.46123 | 7.193E-09 |
| *Mark4* | -3.461 | 2.581E-05 |
| *Ids* | -3.45843 | 8.344E-09 |
| *Capn3* | -3.45741 | 2.653E-05 |
| *Rap1gap2* | -3.45593 | 4.285E-06 |
| *Wdr49* | -3.45302 | 2.261E-04 |
| *Lonrf1* | -3.45092 | 9.189E-04 |
| *Spg11* | -3.45082 | 2.147E-09 |
| *Adamts10* | -3.45082 | 2.867E-07 |
| *Dgkz* | -3.45066 | 2.717E-06 |
| *Nup98* | -3.4505 | 1.375E-08 |
| *Chtf8* | -3.45029 | 2.524E-08 |
| *Igsf9b* | -3.44992 | 9.602E-05 |
| *Cobll1* | -3.44952 | 7.316E-06 |
| *Mrvi1* | -3.44777 | 2.043E-08 |
| *Tns1* | -3.44696 | 1.508E-05 |
| *Plekhd1* | -3.44667 | 2.868E-04 |
| *Ace* | -3.44638 | 6.138E-05 |
| *Ube4a* | -3.44622 | 3.175E-09 |
| *Zfp384* | -3.44613 | 1.578E-06 |
| *Zbtb6* | -3.44574 | 9.355E-06 |
| *Lig3* | -3.44556 | 6.898E-08 |
| *Plekha5* | -3.44517 | 9.012E-06 |
| *Mmgt1* | -3.44499 | 1.002E-06 |
| *Atf7ip* | -3.44464 | 7.236E-08 |
| *Sptbn1* | -3.44348 | 5.933E-07 |
| *Arhgap21* | -3.44282 | 9.097E-07 |
| *Plekhm2* | -3.44247 | 1.008E-04 |
| *Clec9a* | -3.442 | 4.474E-04 |
| *Cftr* | -3.44174 | 1.974E-06 |
| *Antxr1* | -3.44166 | 4.084E-08 |
| *Atxn2l* | -3.44091 | 7.883E-07 |
| *C2cd5* | -3.44087 | 1.371E-06 |
| *Polm* | -3.44069 | 6.772E-05 |
| *Zmiz2* | -3.4403 | 2.221E-07 |
| *Man1b1* | -3.43929 | 4.102E-07 |
| *Tns2* | -3.43781 | 1.122E-05 |
| *Slc9a1* | -3.43763 | 2.474E-05 |
| *Ift80* | -3.43754 | 5.405E-09 |
| *Maml1* | -3.43629 | 5.347E-05 |
| *Eif4a2* | -3.43619 | 6.347E-07 |
| *Samd4* | -3.43588 | 4.315E-06 |
| *5031439G07Rik* | -3.43481 | 5.100E-06 |
| *Ap1s2* | -3.43417 | 8.841E-06 |
| *Col4a2* | -3.43401 | 5.632E-08 |
| *Fxr2* | -3.43397 | 1.313E-06 |
| *Heph* | -3.43374 | 7.326E-06 |
| *Ppp2r5e* | -3.4325 | 9.798E-07 |
| *Ylpm1* | -3.43126 | 6.383E-07 |
| *Atf6* | -3.43101 | 4.861E-08 |
| *Gga3* | -3.43058 | 1.177E-05 |
| *Srf* | -3.43018 | 2.598E-05 |
| *Trak2* | -3.42937 | 8.391E-10 |
| *Oxtr* | -3.42749 | 5.839E-04 |
| *Man2a2* | -3.42454 | 4.668E-06 |
| *Fbxl18* | -3.42289 | 1.251E-04 |
| *Zfp292* | -3.4216 | 6.581E-08 |
| *Ppp6r3* | -3.42118 | 6.971E-09 |
| *Itpkb* | -3.42085 | 8.283E-08 |
| *Chd6* | -3.42014 | 7.882E-12 |
| *Kif26b* | -3.4196 | 4.395E-05 |
| *Ect2l* | -3.41869 | 1.870E-04 |
| *Kat6a* | -3.41742 | 3.470E-08 |
| *Rnf150* | -3.4174 | 5.868E-06 |
| *Ptprj* | -3.41644 | 5.028E-06 |
| *Slc4a7* | -3.41627 | 3.343E-08 |
| *Vwa7* | -3.41556 | 6.636E-04 |
| *Ubqln4* | -3.41481 | 5.098E-06 |
| *Grb10* | -3.41472 | 2.160E-07 |
| *Ston1* | -3.41418 | 3.857E-07 |
| *Traf6* | -3.41365 | 2.222E-06 |
| *Ilf3* | -3.41319 | 9.020E-05 |
| *Ddx19b* | -3.4115 | 1.487E-06 |
| *Gprasp1* | -3.41106 | 4.119E-09 |
| *Wipf2* | -3.41093 | 3.879E-06 |
| *Cse1l* | -3.40932 | 3.400E-06 |
| *Cep126* | -3.40748 | 8.255E-07 |
| *Jak2* | -3.40659 | 5.576E-08 |
| *Rreb1* | -3.40627 | 2.884E-04 |
| *Map3k7* | -3.40546 | 2.982E-07 |
| *Rassf3* | -3.40459 | 2.076E-07 |
| *Flnb* | -3.40433 | 4.389E-06 |
| *Rgl2* | -3.40173 | 1.250E-04 |
| *Mcm3ap* | -3.40095 | 1.800E-06 |
| *Cobl* | -3.40066 | 6.978E-07 |
| *Nlk* | -3.40029 | 4.902E-04 |
| *Nfatc3* | -3.39909 | 7.169E-08 |
| *Pex1* | -3.39814 | 2.133E-04 |
| *Thbs2* | -3.39765 | 3.607E-06 |
| *Satb1* | -3.39705 | 1.732E-05 |
| *Phf21a* | -3.39656 | 2.012E-06 |
| *Prrg3* | -3.39654 | 5.009E-06 |
| *Gab2* | -3.39653 | 1.148E-05 |
| *Asna1* | -3.39628 | 2.114E-07 |
| *Hnrnpd* | -3.39543 | 7.624E-06 |
| *Sfswap* | -3.39528 | 4.354E-07 |
| *Ltbp3* | -3.3935 | 6.622E-07 |
| *Usp33* | -3.39218 | 3.666E-07 |
| *Mgea5* | -3.39099 | 3.094E-06 |
| *Iqgap2* | -3.3907 | 3.131E-06 |
| *Srl* | -3.38942 | 2.256E-04 |
| *Ryr2* | -3.38794 | 1.489E-06 |
| *Gabrp* | -3.38772 | 8.205E-06 |
| *Ccdc82* | -3.38759 | 2.808E-07 |
| *Prpf38b* | -3.3873 | 1.208E-06 |
| *Zfp703* | -3.38716 | 3.392E-05 |
| *Bahcc1* | -3.38702 | 1.030E-04 |
| *Tcaf2* | -3.38695 | 1.429E-04 |
| *R3hdm2* | -3.38651 | 9.289E-07 |
| *Baz1b* | -3.38592 | 2.459E-06 |
| *Pnn* | -3.38571 | 2.970E-07 |
| *Usp12* | -3.38467 | 3.104E-06 |
| *Ccdc14* | -3.38367 | 2.891E-04 |
| *Bbs1* | -3.38285 | 7.040E-08 |
| *Nphp3* | -3.38209 | 1.250E-05 |
| *Fbrsl1* | -3.38083 | 1.735E-06 |
| *Zfp335* | -3.38021 | 4.080E-04 |
| *Ralgapa1* | -3.38012 | 1.535E-06 |
| *Foxj2* | -3.37796 | 2.668E-06 |
| *Ythdf3* | -3.37788 | 5.687E-08 |
| *Kmt5b* | -3.37522 | 1.099E-07 |
| *Ppp1r37* | -3.37433 | 1.674E-04 |
| *Dzip1* | -3.37413 | 4.812E-06 |
| *Tll1* | -3.3733 | 7.013E-05 |
| *Ubr2* | -3.36983 | 1.999E-07 |
| *Ppp6r2* | -3.36824 | 1.844E-06 |
| *Ppm1k* | -3.36822 | 2.891E-05 |
| *Plagl2* | -3.36755 | 1.802E-05 |
| *Slc12a7* | -3.3673 | 2.883E-04 |
| *Parp8* | -3.36714 | 2.970E-06 |
| *Sobp* | -3.36657 | 5.082E-04 |
| *Oxsr1* | -3.36653 | 1.302E-06 |
| *Aasdh* | -3.3654 | 1.216E-06 |
| *Inppl1* | -3.36509 | 1.421E-05 |
| *Ccdc157* | -3.36497 | 3.052E-07 |
| *Selenot* | -3.36444 | 2.406E-06 |
| *Papolg* | -3.36232 | 4.499E-05 |
| *Zfp9* | -3.35998 | 1.041E-04 |
| *Per1* | -3.35938 | 3.630E-08 |
| *Slc7a8* | -3.35861 | 1.890E-04 |
| *Ankfy1* | -3.3584 | 1.367E-06 |
| *Kit* | -3.35759 | 4.372E-06 |
| *Primpol* | -3.35691 | 1.958E-05 |
| *Nr1d2* | -3.35667 | 2.694E-05 |
| *Ccdc112* | -3.35611 | 4.795E-04 |
| *Nemp2* | -3.355 | 4.530E-04 |
| *Il1r1* | -3.35468 | 6.248E-07 |
| *Rgma* | -3.35377 | 1.528E-06 |
| *Patj* | -3.35159 | 7.281E-07 |
| *Gpbp1* | -3.3487 | 6.550E-06 |
| *Tubgcp3* | -3.34775 | 1.548E-08 |
| *Wsb1* | -3.34721 | 4.116E-05 |
| *Fbxo10* | -3.34718 | 4.424E-08 |
| *Caps2* | -3.34656 | 1.512E-04 |
| *Bcl6* | -3.34457 | 8.039E-06 |
| *Ptprd* | -3.3435 | 3.810E-06 |
| *Srrt* | -3.34218 | 3.181E-05 |
| *Atf5* | -3.34192 | 7.373E-05 |
| *Igsf10* | -3.34178 | 3.009E-06 |
| *Sugp2* | -3.34111 | 1.709E-07 |
| *Srrm1* | -3.34048 | 2.570E-04 |
| *Cul3* | -3.34015 | 3.634E-07 |
| *Med23* | -3.33905 | 2.122E-08 |
| *Rlim* | -3.33742 | 7.759E-09 |
| *Arap3* | -3.33533 | 3.029E-04 |
| *Rnf20* | -3.33528 | 1.081E-05 |
| *Nfxl1* | -3.33497 | 2.736E-04 |
| *Dapk1* | -3.33414 | 3.085E-06 |
| *Lmo7* | -3.33385 | 5.335E-08 |
| *Morc3* | -3.33372 | 2.839E-04 |
| *Uvssa* | -3.3332 | 1.214E-05 |
| *Phf3* | -3.33223 | 2.598E-12 |
| *Spred1* | -3.33192 | 1.368E-06 |
| *Trps1* | -3.33136 | 2.413E-08 |
| *Ttll6* | -3.3312 | 1.879E-06 |
| *Yes1* | -3.33113 | 1.596E-07 |
| *Scaf8* | -3.33062 | 1.825E-08 |
| *Ttc7b* | -3.33004 | 1.853E-04 |
| *Samd4b* | -3.32996 | 1.748E-04 |
| *Slc25a40* | -3.32906 | 1.205E-06 |
| *Zfp397* | -3.32854 | 5.576E-09 |
| *Sept2* | -3.32811 | 1.777E-06 |
| *Zfp383* | -3.32737 | 6.512E-05 |
| *Neo1* | -3.32623 | 4.251E-07 |
| *Adnp2* | -3.32456 | 2.923E-05 |
| *Tmx3* | -3.32423 | 1.974E-06 |
| *Tbc1d32* | -3.3236 | 6.347E-07 |
| *Bmi1* | -3.32334 | 2.638E-06 |
| *Gm45902* | -3.32316 | 5.420E-04 |
| *Msl1* | -3.32306 | 7.379E-07 |
| *Mastl* | -3.32292 | 2.441E-06 |
| *Nckap1* | -3.32261 | 2.087E-06 |
| *Emsy* | -3.32242 | 2.484E-06 |
| *Wwc1* | -3.32228 | 2.356E-07 |
| *Rprd1a* | -3.32164 | 4.714E-06 |
| *Ikzf2* | -3.32155 | 7.614E-09 |
| *Akap11* | -3.31948 | 1.056E-07 |
| *Kif3b* | -3.31911 | 4.486E-07 |
| *Atp6v1g2* | -3.31836 | 3.011E-04 |
| *Erf* | -3.31743 | 7.031E-05 |
| *Ptpn3* | -3.31659 | 8.577E-10 |
| *Casd1* | -3.31622 | 2.593E-07 |
| *Dhrs9* | -3.31613 | 2.804E-06 |
| *Thy1* | -3.31456 | 4.897E-06 |
| *Ptprg* | -3.31398 | 8.672E-09 |
| *Plekhg3* | -3.31396 | 1.423E-07 |
| *Cdan1* | -3.31376 | 2.319E-04 |
| *Rapgef1* | -3.31298 | 4.080E-06 |
| *Numa1* | -3.31247 | 1.227E-06 |
| *Lrp4* | -3.31216 | 4.458E-05 |
| *Sh2d4b* | -3.31139 | 1.734E-06 |
| *Rab11fip3* | -3.31134 | 5.028E-06 |
| *Depdc5* | -3.31133 | 5.239E-05 |
| *Gtf3c2* | -3.31107 | 3.194E-05 |
| *Ciz1* | -3.31089 | 4.324E-04 |
| *Ttbk2* | -3.31059 | 4.795E-04 |
| *Top2b* | -3.31035 | 3.905E-06 |
| *Nid1* | -3.30973 | 8.513E-08 |
| *Lrrc4* | -3.30581 | 9.827E-04 |
| *Pcgf2* | -3.30411 | 1.354E-05 |
| *Tet2* | -3.30291 | 6.782E-09 |
| *Tbx3* | -3.30055 | 2.382E-04 |
| *Kdm3b* | -3.29985 | 3.076E-09 |
| *Scaf4* | -3.29954 | 1.556E-04 |
| *Magi1* | -3.29926 | 1.912E-06 |
| *Calcoco1* | -3.29911 | 2.483E-05 |
| *Klhl24* | -3.29806 | 5.559E-07 |
| *Myo6* | -3.29806 | 5.445E-06 |
| *Gpld1* | -3.298 | 1.663E-04 |
| *Nptxr* | -3.2978 | 3.206E-04 |
| *Ubn2* | -3.29687 | 7.546E-10 |
| *Zfp687* | -3.29611 | 1.430E-04 |
| *Cep95* | -3.29541 | 1.053E-04 |
| *9430015G10Rik* | -3.29442 | 1.130E-04 |
| *Stk4* | -3.29399 | 2.432E-07 |
| *Gas2l3* | -3.29278 | 7.711E-06 |
| *Mga* | -3.28938 | 5.089E-13 |
| *Eif5a2* | -3.2884 | 5.794E-04 |
| *Bmf* | -3.28805 | 3.611E-06 |
| *Tgfbr3* | -3.28797 | 8.574E-07 |
| *Wdr19* | -3.28615 | 5.361E-09 |
| *Nol8* | -3.286 | 4.730E-05 |
| *Tppp* | -3.28495 | 5.145E-05 |
| *Zranb1* | -3.28445 | 9.991E-07 |
| *Creb1* | -3.28359 | 4.992E-08 |
| *Snrnp200* | -3.28355 | 2.461E-06 |
| *Tnks2* | -3.28235 | 4.944E-06 |
| *Pxdn* | -3.28223 | 1.560E-05 |
| *Dcaf15* | -3.28193 | 7.419E-05 |
| *Cdc14b* | -3.28095 | 1.842E-07 |
| *Cyld* | -3.28063 | 8.050E-05 |
| *Arhgef38* | -3.28052 | 5.118E-10 |
| *Hdlbp* | -3.27847 | 6.889E-07 |
| *Ptbp3* | -3.27796 | 3.912E-08 |
| *Ptpn4* | -3.27719 | 1.784E-07 |
| *Pdzrn3* | -3.27469 | 1.052E-05 |
| *Ncapd2* | -3.27458 | 4.003E-04 |
| *Lrch3* | -3.2733 | 7.999E-06 |
| *Ash1l* | -3.27306 | 4.930E-08 |
| *Dlg3* | -3.2722 | 7.363E-06 |
| *Sprtn* | -3.2718 | 6.882E-05 |
| *Intu* | -3.27151 | 9.296E-07 |
| *Ppp1r12a* | -3.27016 | 4.114E-05 |
| *Sbf2* | -3.27009 | 1.209E-08 |
| *Golph3l* | -3.26884 | 5.075E-04 |
| *Zyg11b* | -3.26796 | 1.162E-09 |
| *Tenm4* | -3.26454 | 4.024E-08 |
| *Virma* | -3.26413 | 8.696E-05 |
| *Rhob* | -3.26391 | 1.135E-06 |
| *Hjurp* | -3.26322 | 8.787E-05 |
| *Ptpn13* | -3.26256 | 2.939E-07 |
| *Rufy2* | -3.25958 | 1.253E-05 |
| *Farp1* | -3.25846 | 3.863E-06 |
| *Pkp4* | -3.25784 | 7.764E-07 |
| *Akr1c14* | -3.25296 | 3.554E-06 |
| *Tbc1d2b* | -3.25174 | 8.208E-07 |
| *Sorcs2* | -3.24979 | 1.131E-05 |
| *Tent4a* | -3.24955 | 5.075E-04 |
| *Usp28* | -3.24892 | 6.090E-05 |
| *Pitpnm2* | -3.24864 | 3.873E-08 |
| *Cep192* | -3.24821 | 9.391E-08 |
| *Rab35* | -3.2478 | 1.886E-05 |
| *Eif4ebp2* | -3.24495 | 8.881E-07 |
| *Ptpru* | -3.24479 | 1.272E-04 |
| *Uevld* | -3.24407 | 1.208E-05 |
| *Prkdc* | -3.24403 | 2.095E-08 |
| *Zhx1* | -3.24316 | 2.316E-07 |
| *C77080* | -3.24309 | 1.358E-06 |
| *Spast* | -3.2429 | 1.574E-05 |
| *Cfap74* | -3.24037 | 2.671E-10 |
| *Ddi2* | -3.24007 | 8.535E-07 |
| *March6* | -3.23804 | 3.666E-07 |
| *Tanc1* | -3.23789 | 1.776E-08 |
| *Lamb1* | -3.23749 | 1.533E-07 |
| *Mamdc2* | -3.23718 | 4.347E-04 |
| *Ccdc186* | -3.23443 | 9.626E-05 |
| *Pdzd8* | -3.23383 | 1.716E-06 |
| *Vasp* | -3.23329 | 2.140E-05 |
| *Safb2* | -3.23238 | 2.066E-05 |
| *Gnaq* | -3.22963 | 1.107E-07 |
| *Eif4g2* | -3.22723 | 5.338E-05 |
| *Map3k21* | -3.2268 | 1.167E-04 |
| *Tmem8b* | -3.22626 | 3.468E-04 |
| *Trpm7* | -3.22567 | 1.653E-07 |
| *Mtmr10* | -3.22565 | 1.678E-06 |
| *Rlf* | -3.22535 | 2.263E-08 |
| *Inpp5f* | -3.22355 | 1.343E-07 |
| *Plekha7* | -3.22299 | 1.792E-07 |
| *Fbxo45* | -3.22293 | 2.766E-04 |
| *Zfp40* | -3.22273 | 1.424E-06 |
| *Rgl3* | -3.22261 | 2.769E-04 |
| *Supt16* | -3.22247 | 6.725E-06 |
| *Ss18l1* | -3.221 | 4.816E-04 |
| *Ago3* | -3.22071 | 9.887E-09 |
| *Cenpc1* | -3.22001 | 1.327E-06 |
| *Wapl* | -3.2189 | 8.659E-08 |
| *Nktr* | -3.21856 | 6.882E-09 |
| *Elmod1* | -3.21839 | 5.485E-06 |
| *Qk* | -3.21822 | 2.083E-05 |
| *Hs1bp3* | -3.21714 | 8.745E-05 |
| *Icmt* | -3.21702 | 3.802E-06 |
| *Tdrd3* | -3.21692 | 2.454E-05 |
| *Agl* | -3.21684 | 3.806E-05 |
| *Syde2* | -3.21505 | 8.873E-04 |
| *Nrp2* | -3.2144 | 3.009E-06 |
| *Ncoa3* | -3.21342 | 4.107E-04 |
| *Elac1* | -3.21329 | 6.605E-04 |
| *Supt6* | -3.20987 | 1.064E-06 |
| *Tmem94* | -3.20887 | 1.061E-06 |
| *Slc35a3* | -3.20853 | 1.736E-05 |
| *Gm867* | -3.20839 | 8.479E-08 |
| *G2e3* | -3.20817 | 4.745E-06 |
| *Sparcl1* | -3.20786 | 1.257E-04 |
| *Dmxl2* | -3.20726 | 3.986E-05 |
| *Hspa4* | -3.20721 | 8.243E-06 |
| *Dyrk1a* | -3.20709 | 4.260E-05 |
| *Mpdz* | -3.20574 | 4.865E-06 |
| *Macc1* | -3.2052 | 5.291E-04 |
| *Clcn5* | -3.20349 | 5.847E-04 |
| *Rab21* | -3.20206 | 8.577E-06 |
| *Golga3* | -3.20158 | 5.174E-09 |
| *Galc* | -3.20058 | 8.878E-06 |
| *Paip1* | -3.2001 | 1.024E-05 |
| *Atp9b* | -3.19829 | 3.830E-04 |
| *Adgrb2* | -3.19759 | 7.292E-04 |
| *Ap1g1* | -3.19677 | 1.781E-09 |
| *Cdc27* | -3.19669 | 1.991E-06 |
| *Pnpt1* | -3.19595 | 5.730E-06 |
| *Tbc1d12* | -3.19594 | 4.683E-04 |
| *Zfyve16* | -3.19563 | 1.681E-04 |
| *Cdk12* | -3.19266 | 1.494E-05 |
| *Ipo5* | -3.19155 | 6.438E-06 |
| *Hif3a* | -3.19133 | 5.892E-05 |
| *St5* | -3.19024 | 2.902E-05 |
| *Kansl1l* | -3.18991 | 7.945E-10 |
| *Usp48* | -3.18955 | 9.407E-06 |
| *Fcho2* | -3.18946 | 3.614E-06 |
| *Mphosph9* | -3.1866 | 8.974E-05 |
| *Soat1* | -3.18631 | 4.336E-07 |
| *Mpeg1* | -3.18628 | 5.255E-04 |
| *Zswim9* | -3.18616 | 5.640E-04 |
| *Myo9a* | -3.18568 | 2.340E-09 |
| *Zfp592* | -3.18399 | 2.900E-06 |
| *Gatad2b* | -3.1837 | 3.409E-05 |
| *Rnf43* | -3.1835 | 5.255E-04 |
| *Fam84b* | -3.18322 | 4.479E-05 |
| *Kctd12b* | -3.18307 | 8.420E-06 |
| *Plekhh2* | -3.18251 | 4.811E-09 |
| *Lmnb1* | -3.18208 | 7.598E-04 |
| *Nudt12* | -3.18173 | 5.072E-04 |
| *Hltf* | -3.18131 | 4.673E-07 |
| *Ppp4r3a* | -3.18074 | 3.487E-06 |
| *Mme* | -3.17941 | 9.624E-07 |
| *Usp46* | -3.1792 | 2.138E-05 |
| *Col4a1* | -3.1767 | 1.123E-06 |
| *Dnah1* | -3.17604 | 1.286E-07 |
| *Tut4* | -3.17455 | 1.395E-05 |
| *Zc3h12c* | -3.17391 | 1.688E-04 |
| *Srgap2* | -3.17349 | 2.122E-08 |
| *Pla2r1* | -3.17302 | 5.276E-08 |
| *Evc* | -3.17297 | 4.255E-05 |
| *Obsl1* | -3.17294 | 4.295E-04 |
| *Ccdc6* | -3.1726 | 2.274E-06 |
| *Celsr2* | -3.17199 | 1.279E-07 |
| *Pign* | -3.17183 | 6.677E-12 |
| *Zmiz1* | -3.17164 | 1.821E-07 |
| *Maz* | -3.1713 | 7.921E-06 |
| *Rock2* | -3.17113 | 1.763E-06 |
| *Zfp652* | -3.17085 | 1.540E-07 |
| *Eif2ak3* | -3.16986 | 1.679E-05 |
| *Raver2* | -3.16931 | 7.495E-04 |
| *Smg6* | -3.16726 | 3.145E-06 |
| *Abcc1* | -3.16678 | 7.040E-08 |
| *Col4a4* | -3.16646 | 1.027E-07 |
| *Nhsl1* | -3.16622 | 4.884E-07 |
| *Pdgfrb* | -3.16607 | 2.329E-04 |
| *Rasa2* | -3.16547 | 2.219E-05 |
| *Fem1b* | -3.16425 | 4.065E-06 |
| *Tnfsf10* | -3.16424 | 3.317E-05 |
| *Zc3h18* | -3.16394 | 2.323E-06 |
| *Eya3* | -3.16383 | 2.060E-05 |
| *Dclre1c* | -3.16338 | 1.300E-04 |
| *Mfsd4b4* | -3.1632 | 5.265E-04 |
| *Gnptab* | -3.1628 | 4.382E-06 |
| *Irgq* | -3.16167 | 3.073E-04 |
| *Epb41l2* | -3.1613 | 3.702E-05 |
| *Efcab6* | -3.16109 | 3.772E-05 |
| *Adgrl1* | -3.16078 | 7.616E-05 |
| *Zscan29* | -3.16023 | 1.184E-04 |
| *Iqce* | -3.15921 | 1.222E-05 |
| *Eif4g3* | -3.15881 | 2.694E-07 |
| *Atp10a* | -3.15858 | 5.457E-06 |
| *Hnrnpdl* | -3.15818 | 2.156E-04 |
| *Hlf* | -3.15772 | 1.330E-05 |
| *Aoc3* | -3.15634 | 3.421E-05 |
| *Dmpk* | -3.15513 | 1.594E-04 |
| *Mgat4a* | -3.1538 | 3.427E-05 |
| *Pcnt* | -3.15366 | 2.016E-05 |
| *Mybbp1a* | -3.15363 | 6.133E-06 |
| *Prickle2* | -3.15125 | 3.143E-08 |
| *Rb1cc1* | -3.15004 | 2.226E-04 |
| *Dis3* | -3.14992 | 1.109E-04 |
| *Ttc37* | -3.14821 | 9.799E-07 |
| *Grhl2* | -3.14724 | 3.787E-04 |
| *Zfp329* | -3.14482 | 7.073E-06 |
| *Eif2ak2* | -3.14465 | 3.587E-06 |
| *Cenpb* | -3.14461 | 1.496E-05 |
| *Camsap1* | -3.14443 | 9.370E-05 |
| *Atp2a2* | -3.14391 | 4.088E-06 |
| *Ammecr1* | -3.1434 | 3.877E-04 |
| *Thoc2* | -3.14294 | 9.421E-06 |
| *Enpp3* | -3.14237 | 9.087E-06 |
| *Usp37* | -3.14108 | 1.749E-06 |
| *Caprin1* | -3.13986 | 2.683E-05 |
| *Foxn2* | -3.13956 | 1.353E-04 |
| *Setx* | -3.13955 | 1.885E-13 |
| *Tmem106b* | -3.13941 | 2.349E-06 |
| *Per2* | -3.13821 | 2.060E-06 |
| *Zfp532* | -3.13796 | 1.235E-06 |
| *Tmem259* | -3.13755 | 1.423E-04 |
| *R3hdm1* | -3.13711 | 3.840E-07 |
| *Nipal3* | -3.13648 | 4.099E-06 |
| *Ddx19a* | -3.13622 | 2.565E-05 |
| *D330045A20Rik* | -3.13517 | 5.544E-04 |
| *Klhl14* | -3.13473 | 4.914E-04 |
| *Sgsm2* | -3.13459 | 7.731E-05 |
| *Ppp2cb* | -3.13426 | 1.065E-05 |
| *Casz1* | -3.13354 | 1.606E-07 |
| *Abca1* | -3.1322 | 6.201E-05 |
| *Zbtb39* | -3.13192 | 5.011E-04 |
| *Anln* | -3.13098 | 2.367E-04 |
| *Sec23a* | -3.1297 | 2.675E-06 |
| *Klhl18* | -3.12879 | 5.057E-04 |
| *Epg5* | -3.12802 | 2.645E-04 |
| *Tob1* | -3.12713 | 1.132E-05 |
| *Rgs11* | -3.12655 | 1.293E-05 |
| *Ranbp9* | -3.12591 | 2.804E-05 |
| *Chmp4c* | -3.12576 | 2.000E-04 |
| *Zfp142* | -3.12378 | 4.518E-05 |
| *Xpo5* | -3.12197 | 1.637E-06 |
| *Dnajc27* | -3.12032 | 3.145E-04 |
| *Stx4a* | -3.12014 | 1.162E-04 |
| *Fam171b* | -3.1199 | 3.576E-04 |
| *Pabpn1* | -3.11977 | 4.360E-06 |
| *Mbd2* | -3.11847 | 1.480E-05 |
| *Arhgap5* | -3.1179 | 7.316E-06 |
| *Trim12c* | -3.1175 | 4.438E-04 |
| *Fam20b* | -3.11713 | 1.339E-06 |
| *Jarid2* | -3.11659 | 7.054E-06 |
| *Aspn* | -3.11614 | 2.025E-04 |
| *Rc3h2* | -3.11547 | 4.336E-07 |
| *Madd* | -3.11474 | 7.216E-04 |
| *Gm49336* | -3.11451 | 2.520E-06 |
| *Hira* | -3.11396 | 1.771E-04 |
| *Ak9* | -3.1131 | 2.954E-06 |
| *Ep400* | -3.11245 | 9.875E-05 |
| *Carmil1* | -3.11022 | 7.551E-05 |
| *Wdr7* | -3.1088 | 2.637E-05 |
| *Tsc2* | -3.10835 | 6.883E-05 |
| *Rbpj* | -3.10821 | 3.047E-06 |
| *Csnk2a2* | -3.10751 | 3.333E-04 |
| *Lgi2* | -3.10737 | 6.037E-04 |
| *Spns2* | -3.10635 | 1.641E-04 |
| *Cuedc1* | -3.10456 | 1.841E-05 |
| *Ehmt2* | -3.10452 | 8.874E-05 |
| *Tshz1* | -3.10383 | 1.812E-05 |
| *Creb3l2* | -3.10277 | 7.942E-05 |
| *Atxn1* | -3.101 | 8.388E-08 |
| *Foxp2* | -3.10053 | 6.712E-05 |
| *Pkd2* | -3.10029 | 2.951E-06 |
| *Gosr1* | -3.0989 | 4.684E-06 |
| *Luc7l3* | -3.09798 | 4.544E-07 |
| *Trappc11* | -3.09763 | 1.580E-06 |
| *L3mbtl3* | -3.09685 | 5.951E-06 |
| *Vwa8* | -3.09628 | 1.197E-06 |
| *Ap4m1* | -3.09544 | 5.575E-05 |
| *Nrd1* | -3.09254 | 7.986E-05 |
| *Ifnlr1* | -3.09252 | 7.735E-04 |
| *Socs5* | -3.0922 | 1.537E-04 |
| *Zkscan1* | -3.09186 | 2.097E-06 |
| *Cltc* | -3.08925 | 4.946E-05 |
| *Dnah14* | -3.08898 | 1.854E-08 |
| *Tanc2* | -3.08733 | 1.107E-06 |
| *Zfp354c* | -3.08437 | 2.920E-06 |
| *Usp6nl* | -3.08338 | 8.942E-07 |
| *Serpinh1* | -3.08336 | 2.720E-04 |
| *Mgat5* | -3.08285 | 8.064E-08 |
| *Chsy1* | -3.08279 | 5.850E-04 |
| *Asb7* | -3.08241 | 3.527E-04 |
| *BC025920* | -3.08224 | 5.439E-05 |
| *Arsk* | -3.08177 | 1.077E-05 |
| *Secisbp2l* | -3.07723 | 1.033E-05 |
| *Cdkn1b* | -3.07556 | 1.138E-07 |
| *Carf* | -3.07545 | 9.058E-04 |
| *Hivep1* | -3.07526 | 6.429E-06 |
| *Slc38a2* | -3.07441 | 1.610E-05 |
| *Chka* | -3.07389 | 8.324E-04 |
| *Wdr95* | -3.07382 | 2.872E-07 |
| *Tubgcp5* | -3.07375 | 3.825E-04 |
| *Tmtc1* | -3.07324 | 3.378E-05 |
| *Ccng1* | -3.07322 | 4.619E-05 |
| *Ankrd17* | -3.07285 | 1.816E-06 |
| *Smad5* | -3.07196 | 2.638E-06 |
| *Nbas* | -3.06889 | 1.395E-05 |
| *Agfg1* | -3.0685 | 1.827E-07 |
| *Mtmr1* | -3.06835 | 2.712E-08 |
| *Zfp715* | -3.0682 | 2.355E-06 |
| *Socs6* | -3.06664 | 2.179E-05 |
| *Tom1l2* | -3.06564 | 6.151E-06 |
| *Zkscan3* | -3.06543 | 3.126E-07 |
| *Rere* | -3.06459 | 6.712E-06 |
| *Arnt* | -3.06256 | 9.002E-06 |
| *Dvl3* | -3.06186 | 5.602E-06 |
| *Gbp9* | -3.06148 | 3.967E-06 |
| *Fem1c* | -3.05978 | 2.003E-05 |
| *Pik3cb* | -3.05966 | 4.438E-04 |
| *Txlnb* | -3.05936 | 4.555E-04 |
| *Itga6* | -3.05763 | 1.720E-05 |
| *Fam126a* | -3.05572 | 4.648E-06 |
| *Alms1* | -3.05566 | 5.310E-05 |
| *Copg2* | -3.05515 | 5.195E-12 |
| *Tead2* | -3.05393 | 6.951E-06 |
| *Atp6v0a1* | -3.05357 | 1.480E-05 |
| *Polr1a* | -3.05298 | 1.319E-04 |
| *Msrb1* | -3.05211 | 7.403E-04 |
| *Hdac11* | -3.05108 | 8.029E-05 |
| *Naa15* | -3.04982 | 2.833E-05 |
| *Zeb1* | -3.04959 | 8.587E-08 |
| *Tmem87b* | -3.04956 | 1.585E-04 |
| *Xrn1* | -3.04879 | 2.022E-08 |
| *Wdr11* | -3.04811 | 1.714E-06 |
| *Lamc1* | -3.04777 | 9.560E-10 |
| *Polk* | -3.04685 | 9.437E-04 |
| *Slc22a23* | -3.04639 | 3.519E-04 |
| *Tbl1xr1* | -3.04589 | 8.692E-04 |
| *Mospd2* | -3.04521 | 9.181E-05 |
| *Ttc21b* | -3.04425 | 3.324E-04 |
| *Prepl* | -3.04335 | 2.615E-06 |
| *Sec16a* | -3.04327 | 5.562E-07 |
| *Ankrd16* | -3.0382 | 1.875E-05 |
| *Gmfb* | -3.03771 | 1.409E-05 |
| *Fgd4* | -3.03708 | 3.330E-08 |
| *Foxk2* | -3.03582 | 3.715E-05 |
| *Zmynd11* | -3.03504 | 6.107E-07 |
| *Rimklb* | -3.03451 | 1.102E-04 |
| *Tgfbr1* | -3.03445 | 1.133E-07 |
| *Ino80d* | -3.03338 | 6.508E-11 |
| *Eif4g1* | -3.03257 | 2.614E-05 |
| *Kif1b* | -3.03255 | 1.185E-07 |
| *Zfp597* | -3.03075 | 7.130E-04 |
| *Mbd1* | -3.02983 | 1.689E-08 |
| *Ercc6l2* | -3.02698 | 1.899E-08 |
| *Fam193a* | -3.02586 | 7.105E-05 |
| *Pnpla6* | -3.02573 | 3.305E-04 |
| *Zfp942* | -3.02497 | 3.021E-05 |
| *Hsd17b11* | -3.02479 | 2.073E-05 |
| *Wiz* | -3.02448 | 9.599E-06 |
| *Dock1* | -3.02394 | 2.452E-08 |
| *Ypel2* | -3.02228 | 3.850E-05 |
| *Myo18a* | -3.0207 | 2.286E-06 |
| *Dvl1* | -3.01976 | 2.998E-05 |
| *Actr2* | -3.01924 | 5.320E-05 |
| *Rsph10b* | -3.01915 | 1.470E-04 |
| *Cfap57* | -3.01747 | 7.495E-05 |
| *Opa1* | -3.01728 | 2.843E-09 |
| *Arhgap35* | -3.017 | 7.711E-06 |
| *Spock2* | -3.01667 | 1.565E-05 |
| *Med13l* | -3.01647 | 1.558E-06 |
| *Ptpn12* | -3.01448 | 8.015E-05 |
| *Msh6* | -3.0127 | 1.217E-05 |
| *Cip2a* | -3.01118 | 3.447E-04 |
| *Atrn* | -3.00955 | 7.409E-07 |
| *Zdhhc21* | -3.00901 | 4.489E-10 |
| *Purb* | -3.00751 | 4.677E-05 |
| *Mms19* | -3.00734 | 1.400E-04 |
| *Ehf* | -3.00717 | 1.078E-05 |
| *Cep128* | -3.00563 | 1.351E-04 |
| *Eif2s3x* | -3.00537 | 2.172E-06 |
| *Il1rl2* | -3.00314 | 1.237E-05 |
| *Ap3b2* | -3.00026 | 1.979E-04 |
| *Itgb3* | -2.99992 | 8.503E-05 |
| *Prkci* | -2.99934 | 3.147E-05 |
| *Kif19a* | -2.99826 | 2.315E-04 |
| *Hnrnpa0* | -2.99741 | 2.916E-04 |
| *Ecm2* | -2.99715 | 1.403E-04 |
| *Zfp790* | -2.99714 | 3.054E-04 |
| *Vwa3a* | -2.99603 | 7.703E-06 |
| *Zfp568* | -2.99601 | 1.734E-04 |
| *Igfbp5* | -2.996 | 6.926E-04 |
| *Arhgap32* | -2.99569 | 4.836E-07 |
| *Tnnt2* | -2.99487 | 4.074E-05 |
| *Odf2* | -2.99419 | 2.037E-06 |
| *Zc3hav1l* | -2.99357 | 8.120E-04 |
| *Rbfox2* | -2.99331 | 1.485E-09 |
| *Polr3f* | -2.99226 | 7.207E-05 |
| *Lrig2* | -2.99113 | 3.500E-06 |
| *Mrc1* | -2.98963 | 4.271E-04 |
| *Fam98a* | -2.98904 | 1.722E-04 |
| *Dync2h1* | -2.98887 | 1.536E-05 |
| *Bcr* | -2.9878 | 1.258E-07 |
| *Dusp16* | -2.98675 | 6.764E-05 |
| *Ssh1* | -2.98657 | 4.310E-05 |
| *Kdm6b* | -2.98415 | 3.611E-06 |
| *Acap2* | -2.98307 | 1.377E-04 |
| *Rrm2b* | -2.98219 | 4.346E-05 |
| *Per3* | -2.98158 | 4.407E-04 |
| *2310022B05Rik* | -2.97987 | 8.696E-05 |
| *Mrs2* | -2.978 | 1.507E-05 |
| *Yap1* | -2.97601 | 4.013E-06 |
| *Cfap69* | -2.97506 | 4.011E-07 |
| *Fam160b1* | -2.97481 | 1.320E-05 |
| *Dstyk* | -2.97419 | 1.571E-04 |
| *Tnpo1* | -2.97207 | 6.987E-04 |
| *Txndc16* | -2.97206 | 1.134E-04 |
| *C130074G19Rik* | -2.9707 | 6.327E-06 |
| *Rps24* | -2.96911 | 3.378E-04 |
| *Fam91a1* | -2.96878 | 8.709E-06 |
| *Gm609* | -2.96797 | 2.581E-05 |
| *Golga2* | -2.96724 | 9.623E-06 |
| *Rab5c* | -2.96615 | 1.061E-05 |
| *Pogz* | -2.96613 | 5.894E-07 |
| *Tnxb* | -2.96541 | 7.808E-06 |
| *Zfp729b* | -2.96427 | 7.460E-05 |
| *Vat1* | -2.96401 | 4.824E-05 |
| *2610021A01Rik* | -2.96377 | 3.292E-06 |
| *Zbtb1* | -2.96284 | 1.028E-05 |
| *Bmpr1a* | -2.96269 | 5.728E-06 |
| *Cbx3* | -2.96152 | 1.213E-04 |
| *Gcn1l1* | -2.96005 | 3.964E-07 |
| *Svil* | -2.95808 | 2.351E-09 |
| *Iqgap1* | -2.95773 | 1.522E-05 |
| *Slc4a4* | -2.95754 | 7.101E-06 |
| *Smad3* | -2.95747 | 1.132E-05 |
| *Adnp* | -2.95711 | 3.166E-05 |
| *Myo3b* | -2.95689 | 1.030E-08 |
| *Clk1* | -2.95673 | 2.025E-05 |
| *Zfyve26* | -2.95673 | 1.517E-04 |
| *Alg10b* | -2.95667 | 1.100E-05 |
| *Ints3* | -2.95646 | 3.310E-05 |
| *Cacul1* | -2.95591 | 1.655E-05 |
| *Zbtb44* | -2.95536 | 4.332E-04 |
| *Zfp160* | -2.95368 | 2.286E-09 |
| *Fyco1* | -2.95333 | 1.032E-06 |
| *Yme1l1* | -2.95299 | 1.893E-05 |
| *Mansc1* | -2.9526 | 8.984E-04 |
| *Fgd6* | -2.95256 | 1.121E-06 |
| *Foxk1* | -2.95247 | 1.739E-09 |
| *Tle2* | -2.95245 | 3.663E-05 |
| *Arid1b* | -2.95117 | 4.153E-08 |
| *Larp4b* | -2.95069 | 1.008E-05 |
| *Dcaf5* | -2.95028 | 2.154E-06 |
| *Stag1* | -2.94893 | 1.075E-10 |
| *Ddx5* | -2.94864 | 2.595E-04 |
| *Itih5* | -2.9475 | 3.738E-05 |
| *Clasp2* | -2.94704 | 1.236E-06 |
| *Enpep* | -2.94652 | 1.423E-04 |
| *Srek1* | -2.94588 | 3.645E-04 |
| *Sbf1* | -2.94561 | 9.547E-05 |
| *C2cd3* | -2.94511 | 1.111E-07 |
| *1810013L24Rik* | -2.94479 | 2.056E-06 |
| *Fam53c* | -2.94472 | 3.177E-05 |
| *Safb* | -2.94438 | 5.708E-06 |
| *Zfp101* | -2.94428 | 9.495E-06 |
| *Adam22* | -2.94361 | 1.878E-08 |
| *2610507B11Rik* | -2.94239 | 1.378E-05 |
| *Cep162* | -2.94208 | 1.546E-05 |
| *Sp3* | -2.94063 | 3.268E-06 |
| *Pten* | -2.9399 | 9.249E-04 |
| *Nr2c2* | -2.93959 | 8.292E-07 |
| *Zcchc24* | -2.93911 | 6.429E-06 |
| *B3galnt2* | -2.93771 | 5.958E-04 |
| *Wdr37* | -2.9377 | 4.287E-05 |
| *Tpt1* | -2.93757 | 1.174E-04 |
| *Gpalpp1* | -2.93734 | 9.079E-06 |
| *Fmnl3* | -2.93622 | 7.129E-04 |
| *Trpm4* | -2.93607 | 2.271E-04 |
| *Bach1* | -2.93572 | 9.026E-05 |
| *Acaca* | -2.93305 | 4.725E-07 |
| *Prex2* | -2.93286 | 2.275E-04 |
| *Nav2* | -2.93179 | 2.065E-08 |
| *Eif2a* | -2.93036 | 9.970E-06 |
| *Rapgef2* | -2.92922 | 8.952E-05 |
| *Vcpip1* | -2.92845 | 2.117E-05 |
| *Sash1* | -2.92676 | 6.881E-06 |
| *Trp53inp2* | -2.92491 | 6.888E-05 |
| *Rmi1* | -2.9249 | 2.742E-04 |
| *Manea* | -2.92434 | 8.132E-05 |
| *Cnot6l* | -2.92427 | 1.459E-07 |
| *Fam198b* | -2.92416 | 4.150E-04 |
| *Gtf3c3* | -2.92372 | 2.833E-05 |
| *Cep68* | -2.92313 | 1.171E-05 |
| *Tram2* | -2.92313 | 1.827E-04 |
| *Ubr1* | -2.92268 | 1.714E-06 |
| *Axin2* | -2.92222 | 1.512E-06 |
| *Atic* | -2.92034 | 6.391E-06 |
| *Ppfibp1* | -2.92025 | 4.187E-05 |
| *Tulp3* | -2.92017 | 2.233E-05 |
| *Csde1* | -2.9191 | 1.059E-04 |
| *Wnk1* | -2.91856 | 8.301E-08 |
| *Ttc39b* | -2.91835 | 2.101E-11 |
| *Asxl1* | -2.91626 | 4.001E-06 |
| *Cd2ap* | -2.9162 | 2.332E-05 |
| *Zfp445* | -2.9142 | 2.703E-09 |
| *Ndst1* | -2.91394 | 7.651E-07 |
| *Phldb2* | -2.91344 | 2.923E-04 |
| *Prkca* | -2.91234 | 4.533E-04 |
| *Wdr63* | -2.91199 | 2.151E-04 |
| *Sin3a* | -2.90891 | 4.065E-05 |
| *Eps15* | -2.90833 | 5.587E-06 |
| *Taf2* | -2.90809 | 9.192E-04 |
| *Col6a3* | -2.90706 | 9.905E-04 |
| *Sntb1* | -2.90665 | 5.971E-07 |
| *Galnt1* | -2.90626 | 9.782E-05 |
| *Ttc16* | -2.90603 | 5.046E-05 |
| *Spag16* | -2.90582 | 2.707E-06 |
| *Zzef1* | -2.90535 | 8.363E-08 |
| *Phc1* | -2.90409 | 7.072E-04 |
| *Mark2* | -2.90196 | 7.988E-06 |
| *Ubr4* | -2.90172 | 4.078E-07 |
| *Kdm4a* | -2.90094 | 8.364E-04 |
| *Ckap5* | -2.90067 | 1.385E-05 |
| *Mapkapk2* | -2.9 | 8.482E-06 |
| *Fbxo38* | -2.89968 | 4.697E-05 |
| *Myb* | -2.89679 | 2.228E-05 |
| *Castor2* | -2.89659 | 2.040E-04 |
| *Cluh* | -2.89548 | 4.828E-05 |
| *Cep85l* | -2.89495 | 5.391E-04 |
| *Map3k12* | -2.89428 | 8.848E-07 |
| *Gpam* | -2.89396 | 2.973E-05 |
| *Klhl42* | -2.89369 | 1.852E-04 |
| *Agbl5* | -2.89316 | 1.160E-04 |
| *Mcc* | -2.89237 | 1.104E-06 |
| *Itgav* | -2.8912 | 3.624E-09 |
| *Zmym5* | -2.89103 | 1.652E-05 |
| *Bptf* | -2.89023 | 2.287E-07 |
| *Rps12* | -2.88973 | 5.982E-05 |
| *Ints6* | -2.8896 | 1.429E-06 |
| *Zfhx3* | -2.88924 | 2.737E-05 |
| *Col4a3bp* | -2.8885 | 1.014E-04 |
| *Arl14ep* | -2.88829 | 2.539E-05 |
| *BC034090* | -2.88724 | 9.805E-04 |
| *Ppl* | -2.88586 | 1.455E-06 |
| *Kif13a* | -2.88488 | 7.014E-07 |
| *Ak7* | -2.88433 | 9.626E-05 |
| *Herc4* | -2.88428 | 1.247E-04 |
| *Xndc1* | -2.88322 | 1.981E-04 |
| *Phlpp2* | -2.8827 | 3.868E-09 |
| *Ipo9* | -2.882 | 5.482E-04 |
| *Ptgfr* | -2.88189 | 1.010E-05 |
| *Nsd3* | -2.88149 | 3.101E-06 |
| *Rprd2* | -2.88066 | 3.909E-05 |
| *Sh3pxd2b* | -2.87983 | 4.562E-04 |
| *Cipc* | -2.87874 | 9.456E-04 |
| *Med14* | -2.87857 | 1.095E-08 |
| *Slc4a2* | -2.87807 | 1.905E-04 |
| *Vldlr* | -2.87769 | 4.402E-07 |
| *Col27a1* | -2.87758 | 6.550E-06 |
| *Ski* | -2.87743 | 1.470E-05 |
| *Zbed4* | -2.87731 | 1.941E-04 |
| *Mecom* | -2.8759 | 9.581E-06 |
| *Prkg1* | -2.87503 | 8.716E-06 |
| *Ppp2r2c* | -2.87472 | 4.588E-04 |
| *Cog5* | -2.87471 | 1.171E-05 |
| *Tgfbrap1* | -2.87414 | 2.091E-04 |
| *Zfp770* | -2.87306 | 1.978E-04 |
| *Ankib1* | -2.87223 | 4.091E-05 |
| *Gapvd1* | -2.87154 | 8.848E-04 |
| *Kif2a* | -2.86891 | 6.437E-04 |
| *Lcorl* | -2.86873 | 5.562E-07 |
| *Map2k7* | -2.86863 | 3.060E-04 |
| *Piezo1* | -2.86863 | 1.668E-06 |
| *Gclc* | -2.86721 | 2.784E-04 |
| *Glce* | -2.86556 | 1.381E-06 |
| *Celf1* | -2.86518 | 4.385E-05 |
| *Phip* | -2.86411 | 1.538E-06 |
| *Nus1* | -2.86359 | 9.534E-05 |
| *Plcb4* | -2.86301 | 3.980E-05 |
| *Zfp334* | -2.8615 | 5.248E-04 |
| *Gtpbp10* | -2.86008 | 7.655E-04 |
| *Adgrg2* | -2.85967 | 1.007E-05 |
| *Setd5* | -2.8596 | 1.902E-06 |
| *Ulk2* | -2.85947 | 2.323E-05 |
| *Hdac5* | -2.85869 | 3.514E-05 |
| *Phf8* | -2.85827 | 1.358E-05 |
| *Bcl7a* | -2.85816 | 7.592E-05 |
| *Gm38394* | -2.85677 | 2.351E-04 |
| *Aff1* | -2.85595 | 3.053E-09 |
| *Arrdc3* | -2.85536 | 9.491E-04 |
| *Atr* | -2.85343 | 2.712E-08 |
| *Exoc6* | -2.85277 | 4.570E-05 |
| *Prr14l* | -2.85164 | 4.592E-05 |
| *Evpl* | -2.85062 | 6.608E-04 |
| *Mgat3* | -2.85031 | 2.274E-04 |
| *Kat2a* | -2.84697 | 2.836E-04 |
| *Cnot6* | -2.8456 | 2.284E-06 |
| *Znfx1* | -2.84258 | 2.465E-05 |
| *Itpr3* | -2.842 | 2.972E-05 |
| *Mettl4* | -2.84164 | 4.605E-05 |
| *Notch1* | -2.84134 | 1.251E-04 |
| *Dgkd* | -2.84133 | 2.450E-05 |
| *Csnk2a1* | -2.84038 | 1.298E-06 |
| *Usp40* | -2.83967 | 2.576E-07 |
| *Myo1b* | -2.83952 | 2.474E-04 |
| *4931406P16Rik* | -2.83838 | 7.755E-04 |
| *Dsel* | -2.83831 | 4.523E-08 |
| *Arhgap29* | -2.83805 | 5.955E-06 |
| *Ints8* | -2.83622 | 3.939E-04 |
| *Ube3c* | -2.83532 | 2.951E-05 |
| *Arhgef7* | -2.83505 | 3.096E-05 |
| *Src* | -2.83428 | 2.508E-04 |
| *Eln* | -2.83394 | 3.809E-04 |
| *Wrn* | -2.83369 | 1.750E-04 |
| *Mycbpap* | -2.8334 | 1.287E-04 |
| *Fbn1* | -2.833 | 2.391E-07 |
| *Casc3* | -2.83229 | 1.895E-05 |
| *Ipo8* | -2.83146 | 1.765E-04 |
| *Sh3bgrl2* | -2.83014 | 1.135E-04 |
| *Tsga10* | -2.82878 | 1.798E-04 |
| *Ube2o* | -2.82829 | 6.731E-04 |
| *Focad* | -2.82818 | 3.798E-04 |
| *Hnrnpa1* | -2.82813 | 1.954E-06 |
| *Fam171a2* | -2.82801 | 9.188E-04 |
| *Ugcg* | -2.8277 | 6.623E-05 |
| *Bdp1* | -2.82769 | 1.596E-06 |
| *Ppp1cb* | -2.82671 | 4.199E-04 |
| *Btbd7* | -2.82234 | 5.526E-07 |
| *Stox2* | -2.81964 | 3.752E-06 |
| *Mafg* | -2.81919 | 6.473E-04 |
| *Vangl2* | -2.81867 | 7.085E-04 |
| *Rfwd3* | -2.81863 | 5.449E-05 |
| *Isg20l2* | -2.81809 | 8.131E-04 |
| *Tspyl4* | -2.81758 | 1.138E-04 |
| *Sv2b* | -2.81663 | 5.114E-05 |
| *Mcl1* | -2.81647 | 1.373E-04 |
| *Sbno2* | -2.815 | 9.378E-04 |
| *Iqsec1* | -2.81495 | 5.564E-06 |
| *Cstf2* | -2.81316 | 5.198E-05 |
| *Brwd1* | -2.807 | 5.408E-08 |
| *Washc5* | -2.80615 | 1.266E-04 |
| *Mtmr4* | -2.80451 | 7.754E-04 |
| *Thbs3* | -2.80441 | 9.050E-04 |
| *Gria3* | -2.80368 | 2.735E-04 |
| *Itga1* | -2.80264 | 4.196E-05 |
| *Fam126b* | -2.80138 | 8.128E-06 |
| *Fosl2* | -2.80104 | 1.558E-06 |
| *Vamp2* | -2.79964 | 9.505E-04 |
| *Atad2b* | -2.79849 | 6.817E-07 |
| *Tmem164* | -2.79602 | 2.190E-06 |
| *Diaph1* | -2.79356 | 1.378E-04 |
| *Enpp1* | -2.7934 | 3.418E-04 |
| *Pik3r4* | -2.79317 | 9.143E-05 |
| *Man1a* | -2.79244 | 2.187E-05 |
| *Snrnp70* | -2.79183 | 3.427E-05 |
| *Sorbs2* | -2.78976 | 7.447E-06 |
| *Zfp874a* | -2.7896 | 8.890E-05 |
| *Fut2* | -2.78957 | 5.474E-04 |
| *Chd1* | -2.78932 | 3.263E-07 |
| *Pabpc1* | -2.78807 | 3.357E-04 |
| *Utrn* | -2.78785 | 1.661E-07 |
| *Gcc1* | -2.78704 | 3.790E-04 |
| *Relch* | -2.78582 | 1.812E-08 |
| *Kdm4c* | -2.7856 | 1.233E-05 |
| *Ermp1* | -2.78554 | 1.813E-04 |
| *Ppp1r9a* | -2.78513 | 2.582E-04 |
| *Zfp629* | -2.78472 | 5.123E-06 |
| *Cdc37l1* | -2.78268 | 5.800E-05 |
| *Shtn1* | -2.78251 | 4.395E-05 |
| *Car5b* | -2.7825 | 6.430E-04 |
| *Thada* | -2.78125 | 1.276E-04 |
| *Brd1* | -2.78019 | 4.150E-05 |
| *Fchsd2* | -2.77963 | 2.591E-04 |
| *Zcchc7* | -2.77864 | 1.539E-08 |
| *Adamts1* | -2.77653 | 6.679E-04 |
| *Ppp1r15b* | -2.77611 | 9.862E-06 |
| *Usp8* | -2.77569 | 6.846E-05 |
| *Klhl28* | -2.77545 | 7.537E-04 |
| *Thrap3* | -2.77455 | 8.303E-04 |
| *Mlph* | -2.77385 | 8.173E-04 |
| *Sltm* | -2.77373 | 2.705E-05 |
| *Chd2* | -2.77243 | 4.656E-07 |
| *Mknk2* | -2.77238 | 5.945E-05 |
| *Ikbkg* | -2.77134 | 1.991E-05 |
| *Fras1* | -2.77042 | 5.360E-06 |
| *Zfp748* | -2.76762 | 4.902E-06 |
| *Rabgap1l* | -2.76636 | 3.968E-07 |
| *Adamts5* | -2.76619 | 8.718E-04 |
| *Rnf111* | -2.76499 | 1.158E-04 |
| *Mief1* | -2.76384 | 4.597E-04 |
| *Agtpbp1* | -2.76072 | 4.422E-07 |
| *Washc4* | -2.75994 | 1.398E-04 |
| *Bmp2k* | -2.75782 | 1.073E-04 |
| *Hif1a* | -2.75759 | 9.094E-05 |
| *Zdhhc17* | -2.75636 | 9.910E-07 |
| *Susd6* | -2.7548 | 2.105E-05 |
| *Lrrc49* | -2.75454 | 3.518E-04 |
| *Myocd* | -2.75275 | 6.090E-05 |
| *Zfp148* | -2.75224 | 2.172E-06 |
| *Hectd1* | -2.75191 | 5.150E-05 |
| *Zfp984* | -2.749 | 5.747E-04 |
| *Spen* | -2.74853 | 2.373E-05 |
| *Sv2a* | -2.74719 | 2.105E-04 |
| *Ank* | -2.74685 | 7.927E-04 |
| *Zranb3* | -2.74503 | 1.422E-04 |
| *Zfp322a* | -2.74421 | 4.164E-05 |
| *Slc9a8* | -2.74279 | 4.878E-04 |
| *Sfi1* | -2.73799 | 1.504E-05 |
| *Adipor2* | -2.73788 | 2.389E-04 |
| *Pgm2l1* | -2.73736 | 4.704E-04 |
| *Dhx15* | -2.73577 | 4.295E-04 |
| *Lca5* | -2.7335 | 6.946E-06 |
| *Slc35e2* | -2.7332 | 1.947E-04 |
| *Slc41a1* | -2.73243 | 4.010E-05 |
| *Snx14* | -2.73223 | 6.933E-04 |
| *Upf2* | -2.73193 | 5.347E-05 |
| *Zfp976* | -2.73175 | 8.986E-05 |
| *Tctn1* | -2.73127 | 4.960E-04 |
| *Clk4* | -2.72921 | 1.470E-04 |
| *Atad1* | -2.7286 | 1.622E-04 |
| *Synm* | -2.72804 | 2.025E-04 |
| *Npepps* | -2.72763 | 2.219E-06 |
| *Dsg2* | -2.72703 | 4.316E-05 |
| *Map3k20* | -2.72613 | 3.484E-05 |
| *Ascc3* | -2.72395 | 2.697E-06 |
| *Cttnbp2nl* | -2.72218 | 5.974E-05 |
| *Rhou* | -2.72215 | 2.068E-05 |
| *Smarcal1* | -2.72192 | 5.672E-04 |
| *Thra* | -2.71994 | 2.140E-04 |
| *Sema3a* | -2.71918 | 4.773E-04 |
| *Tardbp* | -2.71882 | 9.827E-04 |
| *Ncoa2* | -2.71723 | 2.450E-05 |
| *Amd1* | -2.7171 | 1.290E-04 |
| *Npnt* | -2.71612 | 1.428E-04 |
| *Fancm* | -2.71394 | 2.586E-04 |
| *Hexim1* | -2.71356 | 7.771E-05 |
| *Gtf3c4* | -2.70827 | 1.267E-04 |
| *Dpyd* | -2.70783 | 1.069E-04 |
| *Rpl7a* | -2.70663 | 5.383E-04 |
| *Dcp1a* | -2.70652 | 2.213E-05 |
| *Tsc22d2* | -2.70651 | 4.835E-04 |
| *Klhdc7a* | -2.70417 | 7.444E-04 |
| *Synrg* | -2.70374 | 1.078E-05 |
| *Mturn* | -2.70356 | 6.414E-05 |
| *Smpd4* | -2.70352 | 3.528E-04 |
| *Hivep2* | -2.70154 | 2.720E-05 |
| *Acot11* | -2.70035 | 7.573E-05 |
| *Zfp229* | -2.69962 | 5.522E-05 |
| *Zzz3* | -2.6938 | 8.010E-06 |
| *Smchd1* | -2.69332 | 2.900E-04 |
| *Ldb1* | -2.69257 | 7.216E-04 |
| *Pdpk1* | -2.69096 | 5.643E-04 |
| *Atm* | -2.69056 | 3.023E-06 |
| *Arhgap28* | -2.6901 | 4.729E-04 |
| *Agps* | -2.68874 | 7.166E-08 |
| *Entpd3* | -2.68687 | 9.039E-04 |
| *Ccdc17* | -2.68666 | 7.495E-04 |
| *Stk24* | -2.68635 | 1.761E-05 |
| *Mef2d* | -2.68428 | 2.883E-04 |
| *Sap130* | -2.68399 | 1.912E-04 |
| *Cstf2t* | -2.68395 | 1.520E-04 |
| *Foxo3* | -2.68372 | 1.428E-07 |
| *Mpp5* | -2.67995 | 7.315E-05 |
| *Eef2* | -2.67745 | 9.317E-04 |
| *Snx33* | -2.67715 | 4.903E-04 |
| *AI661453* | -2.67427 | 1.575E-04 |
| *Ccdc66* | -2.67423 | 4.980E-04 |
| *Sipa1l3* | -2.67319 | 3.547E-09 |
| *Flii* | -2.67001 | 7.429E-04 |
| *Nedd4l* | -2.66923 | 5.494E-04 |
| *Crebl2* | -2.66857 | 3.188E-05 |
| *Fubp1* | -2.66855 | 2.817E-06 |
| *Slc28a3* | -2.66746 | 1.205E-05 |
| *Pkd1* | -2.66594 | 2.739E-05 |
| *Fasn* | -2.66513 | 3.685E-04 |
| *Adam10* | -2.66281 | 1.739E-04 |
| *Slf2* | -2.66199 | 2.968E-04 |
| *Rnf40* | -2.66112 | 7.959E-04 |
| *Tia1* | -2.66093 | 7.141E-04 |
| *Fblim1* | -2.65996 | 6.178E-04 |
| *Cab39* | -2.65869 | 6.328E-04 |
| *Abce1* | -2.65832 | 2.263E-04 |
| *Irf6* | -2.65637 | 8.755E-04 |
| *Rbmx* | -2.656 | 5.755E-05 |
| *Birc2* | -2.65318 | 5.129E-05 |
| *Ncoa1* | -2.65204 | 2.212E-06 |
| *Ehmt1* | -2.64829 | 1.859E-04 |
| *Cep250* | -2.64717 | 9.571E-08 |
| *Larp4* | -2.64542 | 3.781E-04 |
| *Ube2h* | -2.64514 | 2.554E-04 |
| *Slc25a28* | -2.64392 | 2.051E-04 |
| *Bmp1* | -2.64237 | 8.407E-06 |
| *Armc2* | -2.64218 | 3.302E-04 |
| *Rsbn1l* | -2.64189 | 5.575E-06 |
| *Bsdc1* | -2.64153 | 2.881E-04 |
| *Mapk1ip1l* | -2.6403 | 6.069E-04 |
| *Vps37a* | -2.63955 | 2.827E-04 |
| *Pgrmc2* | -2.63883 | 5.273E-04 |
| *Phactr4* | -2.63804 | 2.037E-04 |
| *Zfp866* | -2.6377 | 3.073E-04 |
| *Prkacb* | -2.6375 | 1.520E-04 |
| *Plxnb2* | -2.63451 | 7.616E-05 |
| *Herc2* | -2.63265 | 1.185E-10 |
| *Smurf2* | -2.63091 | 4.744E-05 |
| *Sipa1l1* | -2.62991 | 4.079E-09 |
| *Crebrf* | -2.62908 | 4.497E-06 |
| *Nmt2* | -2.62821 | 2.907E-05 |
| *Dync1li2* | -2.62718 | 5.608E-04 |
| *Xpnpep3* | -2.62684 | 1.512E-04 |
| *Bivm* | -2.62639 | 4.383E-05 |
| *Map3k2* | -2.62441 | 3.087E-09 |
| *Phldb1* | -2.62167 | 9.288E-05 |
| *Ogfod1* | -2.61879 | 4.160E-04 |
| *Gzf1* | -2.61675 | 4.648E-04 |
| *Utp14a* | -2.6164 | 7.875E-04 |
| *AI597479* | -2.61254 | 9.982E-04 |
| *Adam9* | -2.61149 | 1.871E-04 |
| *Pcgf3* | -2.6098 | 3.589E-08 |
| *Gigyf2* | -2.60754 | 1.070E-06 |
| *Armcx2* | -2.60703 | 9.737E-04 |
| *Drosha* | -2.60621 | 3.946E-04 |
| *Vav3* | -2.60593 | 4.081E-04 |
| *Slc6a17* | -2.60528 | 1.970E-04 |
| *Cand1* | -2.605 | 5.359E-04 |
| *Man2a1* | -2.60494 | 1.082E-04 |
| *Spef2* | -2.60308 | 4.936E-10 |
| *Ints2* | -2.60231 | 6.978E-07 |
| *Ppfia1* | -2.60173 | 1.517E-04 |
| *Appl1* | -2.59822 | 2.478E-04 |
| *Rabgap1* | -2.59702 | 5.158E-04 |
| *Mast2* | -2.59587 | 2.272E-04 |
| *Nf2* | -2.59397 | 2.369E-04 |
| *Klhl7* | -2.59321 | 2.682E-04 |
| *Sos2* | -2.59219 | 2.182E-05 |
| *Vps13d* | -2.59122 | 3.610E-10 |
| *Tlk1* | -2.58996 | 1.758E-04 |
| *Otud5* | -2.58932 | 8.984E-04 |
| *Btbd8* | -2.58713 | 8.979E-04 |
| *Myh14* | -2.58574 | 5.489E-05 |
| *Ppp2r3a* | -2.58573 | 6.142E-04 |
| *Nipbl* | -2.58538 | 4.376E-04 |
| *Ccdc88c* | -2.58394 | 1.665E-07 |
| *Dtx4* | -2.57961 | 3.965E-04 |
| *Asxl2* | -2.57883 | 2.198E-04 |
| *Rin2* | -2.57878 | 1.741E-04 |
| *Msrb3* | -2.57832 | 2.615E-04 |
| *Ano6* | -2.57822 | 2.905E-04 |
| *Hip1* | -2.57627 | 1.104E-06 |
| *Filip1l* | -2.57449 | 7.472E-04 |
| *4930402H24Rik* | -2.57448 | 7.374E-05 |
| *Ube4b* | -2.57442 | 2.271E-05 |
| *Zfand3* | -2.57421 | 1.857E-04 |
| *Stxbp6* | -2.57417 | 2.889E-04 |
| *Sema6a* | -2.57393 | 1.214E-04 |
| *Zfp518a* | -2.5729 | 9.879E-10 |
| *Zbtb43* | -2.57185 | 7.992E-04 |
| *Pja2* | -2.57027 | 7.464E-04 |
| *Dhcr24* | -2.56717 | 5.537E-04 |
| *Hmgcr* | -2.56706 | 5.191E-04 |
| *Syncrip* | -2.56555 | 4.978E-05 |
| *Gpcpd1* | -2.5649 | 6.206E-04 |
| *Veph1* | -2.56451 | 1.251E-04 |
| *Col7a1* | -2.56433 | 6.176E-05 |
| *Emx2* | -2.56411 | 3.645E-04 |
| *Tcf12* | -2.55939 | 1.291E-05 |
| *March7* | -2.55784 | 8.909E-04 |
| *Rarg* | -2.55759 | 9.047E-04 |
| *Kpna3* | -2.55564 | 5.417E-04 |
| *Hace1* | -2.55245 | 1.391E-04 |
| *Tbc1d5* | -2.55225 | 4.562E-04 |
| *Tmx4* | -2.54076 | 1.866E-04 |
| *Klhl9* | -2.53973 | 9.010E-04 |
| *Vps13a* | -2.53532 | 2.494E-06 |
| *Tjp2* | -2.53464 | 1.426E-04 |
| *Sufu* | -2.53325 | 7.710E-04 |
| *Lrrcc1* | -2.52913 | 1.223E-04 |
| *Slc38a1* | -2.52895 | 8.803E-06 |
| *Nomo1* | -2.52718 | 7.294E-04 |
| *Khdrbs3* | -2.52571 | 2.253E-04 |
| *Rai14* | -2.52438 | 8.841E-08 |
| *Phf20l1* | -2.52144 | 5.716E-05 |
| *Lpp* | -2.51848 | 1.797E-05 |
| *Spopl* | -2.51731 | 1.507E-05 |
| *Atl2* | -2.51658 | 9.904E-04 |
| *Dock4* | -2.51404 | 3.403E-05 |
| *Rpgrip1l* | -2.51256 | 4.892E-07 |
| *Rsf1* | -2.51084 | 1.044E-05 |
| *Khdc4* | -2.51073 | 2.108E-05 |
| *Frmd4b* | -2.51021 | 2.035E-04 |
| *Tet3* | -2.50992 | 1.487E-09 |
| *Sgk3* | -2.50981 | 6.843E-04 |
| *Sema3c* | -2.50916 | 7.661E-05 |
| *Fndc3b* | -2.50822 | 4.046E-04 |
| *Msantd4* | -2.50698 | 4.934E-04 |
| *Pcm1* | -2.50621 | 9.105E-08 |
| *Ankzf1* | -2.50445 | 4.541E-04 |
| *Avl9* | -2.50441 | 9.490E-10 |
| *Cntrl* | -2.50305 | 5.132E-06 |
| *Fpgt* | -2.50179 | 8.259E-04 |
| *Slc25a32* | -2.4988 | 9.280E-04 |
| *Fam168b* | -2.49724 | 5.589E-04 |
| *Shprh* | -2.495 | 1.625E-05 |
| *Sec63* | -2.48823 | 9.378E-04 |
| *Ryk* | -2.48707 | 6.748E-04 |
| *Etv3* | -2.48701 | 4.789E-04 |
| *Lrig3* | -2.48652 | 2.560E-04 |
| *Zmym3* | -2.48288 | 2.338E-06 |
| *Jam2* | -2.4827 | 8.838E-04 |
| *D3Ertd254e* | -2.48102 | 4.541E-04 |
| *Lztfl1* | -2.47956 | 1.061E-05 |
| *Itsn2* | -2.47869 | 2.445E-04 |
| *Topbp1* | -2.47864 | 8.343E-04 |
| *Pan2* | -2.47708 | 3.542E-04 |
| *Plxna1* | -2.47547 | 3.060E-04 |
| *Stx17* | -2.47547 | 8.712E-05 |
| *Abca3* | -2.47409 | 4.997E-04 |
| *Bicd2* | -2.46936 | 1.978E-04 |
| *Prpf40b* | -2.46928 | 6.586E-04 |
| *Epha4* | -2.46601 | 4.960E-04 |
| *Rybp* | -2.46533 | 4.232E-07 |
| *Kansl3* | -2.46418 | 4.573E-04 |
| *Ap3m1* | -2.46384 | 1.865E-04 |
| *Golga4* | -2.46254 | 5.081E-04 |
| *Khnyn* | -2.46155 | 4.032E-04 |
| *Shroom3* | -2.46109 | 4.677E-04 |
| *Washc2* | -2.4602 | 2.738E-04 |
| *Dpy19l3* | -2.45782 | 5.091E-04 |
| *Zfp157* | -2.45689 | 9.319E-05 |
| *Spata13* | -2.45528 | 6.289E-07 |
| *Capn7* | -2.45439 | 3.107E-04 |
| *Slc4a3* | -2.45353 | 1.671E-04 |
| *Nhsl2* | -2.45291 | 4.200E-07 |
| *Abr* | -2.45079 | 1.864E-04 |
| *Itch* | -2.44823 | 7.840E-05 |
| *Mycbp2* | -2.44755 | 3.497E-04 |
| *Tacc1* | -2.44735 | 6.237E-06 |
| *Wdfy3* | -2.44592 | 1.130E-04 |
| *Klhl23* | -2.44414 | 3.764E-05 |
| *Ccdc93* | -2.44344 | 1.430E-06 |
| *Cpsf6* | -2.44241 | 4.034E-04 |
| *Dhx40* | -2.44188 | 6.389E-04 |
| *Lama4* | -2.43922 | 5.318E-06 |
| *Tmem127* | -2.43889 | 7.506E-04 |
| *Nol4l* | -2.43488 | 8.556E-04 |
| *Abcc5* | -2.43405 | 3.011E-07 |
| *Numbl* | -2.4338 | 1.298E-04 |
| *Ahctf1* | -2.43211 | 5.045E-08 |
| *Iws1* | -2.42312 | 8.661E-04 |
| *Mbd5* | -2.4226 | 1.556E-04 |
| *Cbx7* | -2.42032 | 9.430E-05 |
| *Notch2* | -2.41763 | 1.950E-04 |
| *Naalad2* | -2.41737 | 7.974E-04 |
| *Aox3* | -2.41576 | 1.918E-04 |
| *Ibtk* | -2.41365 | 9.158E-04 |
| *Dele1* | -2.41336 | 2.337E-04 |
| *Map4* | -2.40838 | 3.607E-04 |
| *Mysm1* | -2.40813 | 6.573E-11 |
| *Sept8* | -2.4026 | 1.347E-04 |
| *Rictor* | -2.40226 | 1.179E-04 |
| *Uggt2* | -2.40084 | 4.921E-04 |
| *Zfp961* | -2.39918 | 3.805E-04 |
| *Nucks1* | -2.39715 | 7.216E-04 |
| *Mapk8ip1* | -2.39037 | 7.677E-04 |
| *Arhgef18* | -2.38803 | 6.170E-04 |
| *Maco1* | -2.38697 | 1.009E-04 |
| *Dennd4a* | -2.3866 | 1.953E-05 |
| *Cdk17* | -2.38496 | 4.665E-04 |
| *Tbc1d14* | -2.38439 | 8.863E-04 |
| *Birc6* | -2.38138 | 9.864E-10 |
| *Zhx3* | -2.38064 | 3.107E-04 |
| *Kirrel* | -2.37715 | 2.097E-04 |
| *Rab3gap1* | -2.37522 | 7.414E-04 |
| *Gprc5b* | -2.37453 | 1.792E-04 |
| *Ehbp1l1* | -2.37367 | 2.750E-04 |
| *Gxylt1* | -2.37092 | 1.120E-04 |
| *Spty2d1* | -2.37065 | 3.637E-04 |
| *Cul7* | -2.37008 | 5.135E-08 |
| *Nxf1* | -2.36941 | 9.341E-05 |
| *Asap2* | -2.36919 | 6.470E-04 |
| *Fxr1* | -2.36066 | 8.941E-04 |
| *Elp1* | -2.35609 | 3.415E-04 |
| *Odf2l* | -2.35558 | 9.985E-04 |
| *Dhx8* | -2.35476 | 8.649E-04 |
| *Tbc1d23* | -2.35378 | 6.835E-04 |
| *Cyp4f17* | -2.35342 | 6.922E-04 |
| *4833420G17Rik* | -2.35314 | 9.776E-05 |
| *Insr* | -2.3523 | 8.176E-06 |
| *Ddx60* | -2.35207 | 2.568E-04 |
| *Kdm5a* | -2.34848 | 9.473E-04 |
| *Micall1* | -2.34501 | 2.223E-10 |
| *Alkbh8* | -2.3414 | 5.119E-05 |
| *Limch1* | -2.33389 | 3.754E-05 |
| *Numb* | -2.32353 | 2.976E-04 |
| *Lin7c* | -2.32321 | 8.515E-06 |
| *Cul9* | -2.321 | 2.820E-04 |
| *Otud7b* | -2.31924 | 9.002E-05 |
| *Ppp2r5c* | -2.31459 | 9.583E-04 |
| *Vezt* | -2.31404 | 2.293E-04 |
| *Gfpt1* | -2.31174 | 2.577E-08 |
| *Tbc1d1* | -2.30978 | 1.926E-04 |
| *Senp1* | -2.30965 | 6.384E-04 |
| *Arhgap42* | -2.30721 | 1.035E-06 |
| *Vps50* | -2.30711 | 8.415E-05 |
| *Osbpl3* | -2.30662 | 2.153E-04 |
| *Parg* | -2.30175 | 8.538E-05 |
| *Nek1* | -2.2942 | 2.406E-05 |
| *Fsd1l* | -2.2814 | 1.309E-04 |
| *Man1a2* | -2.27201 | 9.372E-04 |
| *Mecp2* | -2.26052 | 6.726E-08 |
| *Prpf4b* | -2.25365 | 7.486E-05 |
| *Tnrc6b* | -2.25125 | 2.359E-04 |
| *Tmprss2* | -2.2363 | 8.239E-04 |
| *Pag1* | -2.23422 | 1.946E-04 |
| *Maml2* | -2.2339 | 2.097E-04 |
| *Atp8a1* | -2.23287 | 5.953E-09 |
| *Ttc28* | -2.23264 | 2.430E-04 |
| *C330007P06Rik* | -2.21546 | 1.019E-04 |
| *Sf3b3* | -2.20475 | 9.349E-04 |
| *Atxn3* | -2.19812 | 2.410E-05 |
| *Trim39* | -2.19293 | 3.159E-04 |
| *Pak3* | -2.18458 | 3.210E-04 |
| *Casc1* | -2.18414 | 1.251E-04 |
| *Abca6* | -2.18181 | 2.478E-04 |
| *Rbm5* | -2.17337 | 3.213E-04 |
| *Adcy7* | -2.1725 | 7.277E-04 |
| *Cnot7* | -2.16672 | 6.816E-04 |
| *Zfp507* | -2.16288 | 3.515E-05 |
| *Dnah2* | -2.15769 | 2.717E-07 |
| *Krit1* | -2.15396 | 6.848E-04 |
| *D5Ertd579e* | -2.15054 | 3.222E-06 |
| *Meis1* | -2.14773 | 6.314E-04 |
| *Rfx3* | -2.14081 | 6.624E-06 |
| *Ggta1* | -2.13688 | 1.006E-04 |
| *Pikfyve* | -2.1316 | 6.193E-07 |
| *Zfp398* | -2.13024 | 3.018E-04 |
| *Cdk5rap2* | -2.11895 | 3.680E-04 |
| *Mitf* | -2.10607 | 1.946E-04 |
| *Frs2* | -2.10181 | 8.304E-04 |
| *Tcaf1* | -2.09727 | 7.236E-08 |
| *Ablim1* | -2.09433 | 8.957E-04 |
| *Usp53* | -2.08975 | 9.479E-04 |
| *Osbpl8* | -2.08878 | 8.491E-06 |
| *Cspp1* | -2.08007 | 4.178E-04 |
| *Ptprt* | -2.07054 | 8.696E-05 |
| *Atrx* | -2.06226 | 2.463E-06 |
| *Cfap54* | -2.04646 | 3.858E-06 |
| *Dlc1* | -2.04551 | 5.082E-04 |
| *Ank3* | -2.04459 | 2.721E-04 |
| *Casp8ap2* | -2.03303 | 1.276E-04 |
| *Arhgap31* | -2.03042 | 5.331E-04 |
| *Sec14l1* | -2.02681 | 9.954E-04 |
| *Ston2* | -2.01822 | 1.892E-07 |
| *Ints1* | -2.01186 | 9.570E-04 |
| *Zfp974* | -2.01007 | 2.853E-05 |
| *Ddx58* | -2.00735 | 8.173E-04 |
| *Tomm7* | 2.002282 | 6.741E-08 |
| *Gm14322* | 2.002289 | 8.080E-06 |
| *Stmn1* | 2.002549 | 2.072E-07 |
| *Esam* | 2.00332 | 3.510E-10 |
| *Aamdc* | 2.005223 | 1.327E-09 |
| *Tnfaip8l2* | 2.008292 | 4.419E-05 |
| *Pxmp2* | 2.009188 | 1.965E-06 |
| *Tapbp* | 2.010559 | 6.796E-08 |
| *H2-Eb1* | 2.010651 | 1.257E-04 |
| *Emc9* | 2.015382 | 7.040E-08 |
| *Ap2s1* | 2.0173 | 1.350E-07 |
| *Ctss* | 2.019621 | 1.366E-04 |
| *Polr2c* | 2.020721 | 7.332E-10 |
| *Sri* | 2.021684 | 7.504E-11 |
| *Selenof* | 2.022131 | 6.936E-08 |
| *Polr3h* | 2.023232 | 7.703E-08 |
| *Lrrc25* | 2.024978 | 2.673E-04 |
| *Trp53rkb* | 2.027017 | 1.403E-05 |
| *Ndufc2* | 2.027677 | 4.743E-09 |
| *Tradd* | 2.028092 | 6.543E-11 |
| *Arl5c* | 2.028448 | 6.364E-05 |
| *Gdf15* | 2.029705 | 3.911E-05 |
| *Tmem98* | 2.030031 | 4.139E-09 |
| *Psmb2* | 2.032623 | 4.493E-09 |
| *Fuom* | 2.034155 | 4.254E-10 |
| *Tsc22d3* | 2.035101 | 1.482E-07 |
| *Tmem140* | 2.037613 | 2.206E-06 |
| *Morn2* | 2.038773 | 3.094E-08 |
| *Mgmt* | 2.041489 | 1.405E-10 |
| *S100a3* | 2.045412 | 1.319E-04 |
| *Fcgr4* | 2.045898 | 5.754E-04 |
| *Chadl* | 2.047035 | 6.031E-04 |
| *Ndufa6* | 2.048622 | 5.584E-09 |
| *Jund* | 2.050509 | 1.443E-09 |
| *9430038I01Rik* | 2.050979 | 2.545E-05 |
| *Gm14327* | 2.051075 | 2.387E-05 |
| *Mrps18c* | 2.051603 | 4.078E-07 |
| *Macrod1* | 2.052329 | 2.189E-10 |
| *Acaa1b* | 2.05433 | 5.986E-05 |
| *Metrnl* | 2.054826 | 1.338E-09 |
| *Atp5j2* | 2.05727 | 4.376E-06 |
| *Ly96* | 2.057634 | 7.653E-09 |
| *Rps27* | 2.058025 | 6.405E-07 |
| *Spink4* | 2.060834 | 3.248E-04 |
| *Lpar5* | 2.062344 | 8.056E-04 |
| *Rps19bp1* | 2.062744 | 8.183E-10 |
| *Aqp5* | 2.063666 | 1.642E-05 |
| *Get4* | 2.064315 | 1.340E-10 |
| *Wfdc2* | 2.068698 | 6.169E-07 |
| *Tmem121* | 2.06944 | 1.189E-04 |
| *Asf1b* | 2.069658 | 1.361E-05 |
| *Poln* | 2.071673 | 4.518E-09 |
| *Phpt1* | 2.071887 | 2.774E-10 |
| *Cdkn1c* | 2.072889 | 6.602E-06 |
| *Tmem176a* | 2.073341 | 1.002E-08 |
| *1110008P14Rik* | 2.074014 | 4.706E-07 |
| *Mettl5* | 2.075942 | 3.102E-07 |
| *mt-Atp8* | 2.07714 | 1.420E-07 |
| *Lsr* | 2.077417 | 5.158E-09 |
| *Sdhb* | 2.078302 | 1.780E-09 |
| *Sct* | 2.078521 | 1.049E-04 |
| *Prss53* | 2.079765 | 2.558E-05 |
| *Psmd11* | 2.084073 | 2.305E-06 |
| *Mrps28* | 2.084343 | 3.321E-12 |
| *Srp19* | 2.084541 | 1.147E-06 |
| *Rpl13* | 2.085839 | 1.533E-08 |
| *Chmp6* | 2.086766 | 5.107E-12 |
| *Psmb10* | 2.088299 | 3.224E-10 |
| *Stub1* | 2.093583 | 7.727E-10 |
| *Rab20* | 2.093822 | 4.442E-09 |
| *Icam2* | 2.094844 | 8.672E-09 |
| *S100a16* | 2.095159 | 4.932E-10 |
| *Fdx1l* | 2.095575 | 3.730E-09 |
| *AI413582* | 2.096406 | 4.881E-07 |
| *Tmem50a* | 2.098985 | 8.232E-09 |
| *Mrpl51* | 2.099559 | 1.383E-10 |
| *Ppp1r35* | 2.100155 | 4.084E-08 |
| *Cidea* | 2.100231 | 1.261E-10 |
| *C1rl* | 2.101074 | 1.600E-07 |
| *Otulin* | 2.102725 | 2.612E-11 |
| *Pop7* | 2.104783 | 1.057E-08 |
| *Prr15l* | 2.10604 | 2.853E-10 |
| *Nbl1* | 2.10665 | 8.529E-09 |
| *Tusc2* | 2.10789 | 1.550E-09 |
| *2310011J03Rik* | 2.10855 | 1.737E-10 |
| *Fbxo2* | 2.10951 | 2.971E-05 |
| *Fam213b* | 2.113378 | 3.513E-10 |
| *Aprt* | 2.113593 | 2.850E-10 |
| *Prelid1* | 2.113923 | 3.953E-07 |
| *Chrac1* | 2.114899 | 2.812E-09 |
| *Hs3st6* | 2.115932 | 6.397E-07 |
| *Crlf1* | 2.119154 | 1.470E-04 |
| *Clic3* | 2.119309 | 1.888E-08 |
| *Psmb3* | 2.124834 | 1.176E-09 |
| *Morn5* | 2.12513 | 4.588E-09 |
| *Bola3* | 2.13034 | 2.403E-13 |
| *Yif1a* | 2.132293 | 1.131E-11 |
| *Atp5l* | 2.132731 | 2.149E-08 |
| *Tspan3* | 2.133693 | 1.304E-09 |
| *Zmat5* | 2.133872 | 7.313E-12 |
| *Krtcap3* | 2.133913 | 7.682E-11 |
| *Rdh14* | 2.135353 | 1.297E-11 |
| *Ndufaf6* | 2.137136 | 3.168E-08 |
| *Naxe* | 2.137476 | 5.593E-09 |
| *Mrpl46* | 2.138941 | 5.089E-12 |
| *Fkbp2* | 2.14119 | 3.775E-09 |
| *Cst3* | 2.142889 | 3.130E-08 |
| *Mapk4* | 2.143084 | 4.301E-08 |
| *Pycr2* | 2.14328 | 3.444E-09 |
| *Pebp1* | 2.146455 | 3.621E-09 |
| *Zfyve21* | 2.146583 | 3.728E-11 |
| *Nr0b1* | 2.146728 | 1.853E-04 |
| *Selenos* | 2.14685 | 2.104E-10 |
| *Dusp28* | 2.150692 | 1.611E-10 |
| *Ropn1l* | 2.156458 | 5.416E-09 |
| *Selplg* | 2.157025 | 1.170E-05 |
| *Mrpl17* | 2.157985 | 3.953E-09 |
| *Tmem151a* | 2.158679 | 1.162E-11 |
| *Mfsd5* | 2.161303 | 3.285E-12 |
| *Mrpl36* | 2.163234 | 1.923E-09 |
| *Uqcrc1* | 2.163687 | 6.639E-10 |
| *1700007K13Rik* | 2.165409 | 9.364E-08 |
| *Id3* | 2.167394 | 3.542E-10 |
| *Gde1* | 2.169324 | 1.429E-09 |
| *Rd3* | 2.169644 | 1.682E-07 |
| *Minos1* | 2.179321 | 5.690E-07 |
| *Sdhaf1* | 2.179524 | 6.041E-11 |
| *Bpifb1* | 2.17975 | 1.867E-05 |
| *Dpm3* | 2.189969 | 3.379E-10 |
| *Akip1* | 2.190657 | 2.433E-10 |
| *Ogfod3* | 2.192172 | 1.008E-11 |
| *Inka1* | 2.19402 | 2.130E-05 |
| *C1qa* | 2.194385 | 3.434E-06 |
| *Gfer* | 2.195602 | 2.403E-08 |
| *Adora2b* | 2.195967 | 1.150E-05 |
| *Cldn15* | 2.196514 | 6.640E-10 |
| *Iscu* | 2.197799 | 7.929E-10 |
| *Ifi205* | 2.200616 | 8.789E-07 |
| *Gemin7* | 2.20319 | 1.004E-12 |
| *Tmed3* | 2.20404 | 1.375E-08 |
| *Gm6710* | 2.206159 | 3.059E-04 |
| *Alas2* | 2.20874 | 4.200E-07 |
| *Mea1* | 2.212523 | 6.890E-10 |
| *Ypel3* | 2.213377 | 3.401E-07 |
| *Ppp1ca* | 2.213599 | 1.481E-10 |
| *Nsmce1* | 2.213761 | 1.105E-10 |
| *Psmb9* | 2.218688 | 8.931E-10 |
| *Ethe1* | 2.225971 | 1.972E-12 |
| *Dexi* | 2.232667 | 2.652E-13 |
| *Mrps16* | 2.232705 | 2.541E-11 |
| *Trf* | 2.234783 | 2.677E-08 |
| *Lamtor2* | 2.235378 | 4.942E-11 |
| *Ncf4* | 2.235764 | 5.336E-05 |
| *Tomm20l* | 2.237545 | 6.978E-04 |
| *Emc6* | 2.239419 | 5.158E-11 |
| *Card19* | 2.241615 | 1.421E-11 |
| *Mt2* | 2.242054 | 4.113E-09 |
| *Snrpb* | 2.24833 | 1.584E-10 |
| *Adssl1* | 2.248834 | 1.754E-04 |
| *Krt8* | 2.249451 | 8.661E-08 |
| *Hypk* | 2.250139 | 1.312E-08 |
| *4930404N11Rik* | 2.254407 | 1.272E-07 |
| *Smpd5* | 2.257105 | 6.531E-05 |
| *1700001C02Rik* | 2.257859 | 1.590E-07 |
| *Mydgf* | 2.258056 | 1.405E-10 |
| *Bin2* | 2.258996 | 2.542E-04 |
| *Upk3bl* | 2.259567 | 1.323E-04 |
| *D8Ertd738e* | 2.259968 | 1.171E-10 |
| *Gzmm* | 2.262226 | 2.274E-05 |
| *Pex7* | 2.262357 | 4.902E-06 |
| *Pla2g12a* | 2.264152 | 4.582E-09 |
| *Batf* | 2.26597 | 2.759E-04 |
| *Gm45716* | 2.268021 | 1.666E-05 |
| *Tuba1a* | 2.268596 | 6.578E-10 |
| *Lamtor5* | 2.27292 | 7.936E-10 |
| *Trib3* | 2.276691 | 1.336E-05 |
| *Rnh1* | 2.276787 | 4.894E-09 |
| *Spr* | 2.276862 | 2.439E-11 |
| *Emc4* | 2.277316 | 8.064E-11 |
| *Pnp2* | 2.277466 | 3.212E-09 |
| *Nudt14* | 2.284525 | 1.738E-10 |
| *Riiad1* | 2.285905 | 1.510E-07 |
| *Pycard* | 2.286213 | 1.241E-07 |
| *Rspo1* | 2.286576 | 5.095E-10 |
| *Sh3bgrl3* | 2.286621 | 1.011E-06 |
| *Rsph9* | 2.288097 | 6.833E-11 |
| *Mrpl41* | 2.288142 | 6.733E-11 |
| *Tsen34* | 2.289364 | 2.529E-10 |
| *2610028H24Rik* | 2.289956 | 5.431E-11 |
| *Cib1* | 2.290468 | 1.703E-09 |
| *Pam16* | 2.290524 | 3.859E-13 |
| *Tmem208* | 2.291448 | 1.286E-08 |
| *Ndufaf3* | 2.291611 | 2.085E-11 |
| *Tuba1b* | 2.29218 | 7.147E-10 |
| *1190005I06Rik* | 2.294888 | 1.047E-05 |
| *Tspan1* | 2.297937 | 1.326E-11 |
| *Kdf1* | 2.301935 | 6.322E-10 |
| *Pacsin1* | 2.301992 | 3.677E-04 |
| *Mrpl34* | 2.303234 | 5.066E-11 |
| *Rpp21* | 2.303557 | 1.648E-13 |
| *Stkld1* | 2.303669 | 6.956E-08 |
| *Fis1* | 2.304048 | 7.576E-11 |
| *Comtd1* | 2.306155 | 2.340E-07 |
| *Bloc1s4* | 2.307732 | 1.279E-11 |
| *Gng5* | 2.308009 | 9.145E-10 |
| *Nenf* | 2.308405 | 2.101E-10 |
| *Fbp1* | 2.309366 | 4.554E-05 |
| *Fbxl15* | 2.311963 | 1.161E-06 |
| *Snrpd2* | 2.318475 | 1.658E-10 |
| *G430095P16Rik* | 2.319983 | 1.412E-04 |
| *Serpinb1a* | 2.320007 | 1.962E-08 |
| *Dok2* | 2.325762 | 1.782E-04 |
| *Uqcc3* | 2.326836 | 2.226E-11 |
| *Timm22* | 2.32961 | 1.647E-14 |
| *Ppdpf* | 2.331379 | 9.160E-11 |
| *Aimp2* | 2.331792 | 3.083E-12 |
| *Dynll1* | 2.332493 | 4.342E-10 |
| *Lat* | 2.332503 | 7.159E-06 |
| *Atg101* | 2.338207 | 1.712E-11 |
| *Guk1* | 2.338363 | 9.128E-12 |
| *Nutf2-ps1* | 2.338655 | 6.671E-05 |
| *Anapc13* | 2.343238 | 1.789E-10 |
| *2210016F16Rik* | 2.343458 | 3.349E-11 |
| *Tmem41a* | 2.35114 | 4.066E-13 |
| *Trex1* | 2.351213 | 2.930E-07 |
| *Dbndd2* | 2.353115 | 7.575E-07 |
| *Cox6c* | 2.353283 | 8.478E-07 |
| *Fam174a* | 2.353558 | 8.503E-11 |
| *Uqcc2* | 2.355211 | 4.142E-10 |
| *Oaz2* | 2.355267 | 1.933E-11 |
| *Rpl36a-ps1* | 2.355654 | 4.220E-06 |
| *Siva1* | 2.357055 | 3.236E-13 |
| *Pdlim4* | 2.3571 | 5.158E-11 |
| *Tbcb* | 2.360354 | 2.608E-11 |
| *Cadm1* | 2.36205 | 2.051E-10 |
| *Dctn3* | 2.362467 | 1.104E-11 |
| *Cebpd* | 2.365027 | 6.312E-09 |
| *Tmem147* | 2.365125 | 4.135E-12 |
| *2310034G01Rik* | 2.367262 | 4.636E-13 |
| *Mcrip1* | 2.370768 | 1.740E-10 |
| *Il2rg* | 2.371076 | 5.179E-05 |
| *Tmsb4x* | 2.372651 | 6.798E-07 |
| *Gcat* | 2.375399 | 9.004E-11 |
| *Ssna1* | 2.375751 | 8.815E-11 |
| *Tmem107* | 2.376681 | 8.002E-12 |
| *Gm26558* | 2.379389 | 4.868E-05 |
| *Cdpf1* | 2.382906 | 3.083E-11 |
| *Prg4* | 2.383218 | 2.998E-07 |
| *Msln* | 2.391497 | 2.440E-10 |
| *Eif6* | 2.394443 | 4.010E-12 |
| *Timm17b* | 2.396051 | 7.133E-11 |
| *Nme4* | 2.400387 | 2.200E-08 |
| *Itgb7* | 2.400485 | 6.670E-07 |
| *Dnajc15* | 2.400821 | 1.831E-09 |
| *Ube2l6* | 2.401787 | 5.225E-07 |
| *Tst* | 2.402084 | 2.243E-11 |
| *Cfb* | 2.402118 | 1.014E-09 |
| *Gmppb* | 2.404647 | 3.007E-07 |
| *Mpc2* | 2.405592 | 7.579E-10 |
| *Bok* | 2.408186 | 3.419E-12 |
| *Cox4i2* | 2.410628 | 2.157E-05 |
| *Eif5a* | 2.414614 | 7.003E-11 |
| *Cycs* | 2.416802 | 1.320E-08 |
| *Ngb* | 2.417708 | 1.797E-08 |
| *Oip5* | 2.417812 | 2.022E-05 |
| *Aldoc* | 2.418583 | 1.002E-07 |
| *Atp5e* | 2.420054 | 1.259E-09 |
| *Nkx6-2* | 2.420823 | 7.331E-05 |
| *Shisa8* | 2.424203 | 1.187E-08 |
| *Snrpg* | 2.426485 | 8.583E-11 |
| *Cox5b* | 2.432418 | 9.931E-11 |
| *Cd81* | 2.434549 | 6.144E-11 |
| *Treml1* | 2.435455 | 4.672E-06 |
| *Itga2b* | 2.435837 | 7.306E-09 |
| *Spint2* | 2.436052 | 8.007E-11 |
| *Lgals7* | 2.437806 | 1.107E-09 |
| *Sostdc1* | 2.443399 | 2.572E-05 |
| *Klk1* | 2.443978 | 4.668E-06 |
| *3110070M22Rik* | 2.448555 | 1.485E-05 |
| *Creg1* | 2.451284 | 1.994E-11 |
| *Ten1* | 2.451526 | 1.415E-11 |
| *Hcfc1r1* | 2.451555 | 1.398E-08 |
| *Tmsb15b1* | 2.453691 | 2.669E-11 |
| *H2afv* | 2.456656 | 4.260E-12 |
| *Gpx1* | 2.458999 | 1.249E-05 |
| *Clic1* | 2.460413 | 6.128E-11 |
| *Cyb561d2* | 2.460806 | 3.736E-08 |
| *Cd52* | 2.461263 | 7.848E-05 |
| *Guca1a* | 2.462887 | 2.162E-08 |
| *Tmsb10* | 2.467286 | 6.229E-11 |
| *Nme3* | 2.467647 | 1.606E-12 |
| *Nfkbia* | 2.46892 | 2.223E-10 |
| *Smim12* | 2.471195 | 9.050E-14 |
| *Clpp* | 2.471231 | 1.378E-09 |
| *Cmtm7* | 2.474723 | 2.298E-12 |
| *Tbcc* | 2.475509 | 4.348E-14 |
| *Mpv17l2* | 2.47583 | 3.710E-13 |
| *Myl12a* | 2.476328 | 7.598E-10 |
| *Mpc1* | 2.481509 | 7.947E-12 |
| *Atp5g3* | 2.487002 | 4.551E-11 |
| *Polr2k* | 2.487734 | 9.561E-08 |
| *Ackr1* | 2.487914 | 2.357E-05 |
| *1500011B03Rik* | 2.488935 | 1.117E-09 |
| *Ephb1* | 2.491658 | 1.479E-05 |
| *S100a4* | 2.492139 | 3.291E-09 |
| *Cenpx* | 2.492303 | 3.854E-11 |
| *Fkbp11* | 2.497543 | 1.215E-09 |
| *Ndufb4* | 2.497656 | 3.304E-10 |
| *Ctsw* | 2.50327 | 7.353E-04 |
| *Cenpw* | 2.505046 | 1.095E-09 |
| *Tspan4* | 2.505115 | 2.929E-09 |
| *Mrps26* | 2.506392 | 3.285E-12 |
| *Tmem213* | 2.508676 | 2.263E-04 |
| *Tmem42* | 2.520927 | 2.635E-12 |
| *Spc24* | 2.521199 | 3.023E-06 |
| *Fxyd5* | 2.523981 | 2.140E-06 |
| *Dda1* | 2.524458 | 2.487E-14 |
| *Cyc1* | 2.524785 | 2.384E-10 |
| *Pgls* | 2.524914 | 4.622E-08 |
| *Tma7* | 2.525023 | 5.136E-11 |
| *Mrps34* | 2.527584 | 1.444E-12 |
| *Cyp2c23* | 2.528782 | 1.212E-06 |
| *2510002D24Rik* | 2.529989 | 2.942E-13 |
| *Nme1* | 2.536126 | 7.029E-12 |
| *Bst1* | 2.546656 | 3.683E-06 |
| *Dbi* | 2.550422 | 7.891E-11 |
| *Lefty1* | 2.550987 | 6.155E-08 |
| *Laptm5* | 2.551678 | 2.739E-07 |
| *Edf1* | 2.553372 | 1.333E-11 |
| *H2-DMa* | 2.554298 | 1.886E-07 |
| *Tpgs1* | 2.558493 | 4.091E-14 |
| *Mt3* | 2.560454 | 1.498E-10 |
| *Mrpl27* | 2.561108 | 5.900E-12 |
| *Cebpzos* | 2.563957 | 1.421E-11 |
| *Chchd6* | 2.565626 | 2.654E-12 |
| *Mrpl23* | 2.567097 | 3.092E-12 |
| *Cox7a1* | 2.568016 | 6.304E-05 |
| *Krtcap2* | 2.570195 | 7.649E-11 |
| *Itgb2* | 2.571348 | 2.500E-07 |
| *H2-M3* | 2.57486 | 2.792E-09 |
| *Arhgdig* | 2.577338 | 4.659E-12 |
| *Plin5* | 2.581268 | 8.143E-13 |
| *Gstm1* | 2.582935 | 4.002E-11 |
| *Pf4* | 2.584647 | 5.375E-10 |
| *Gjb5* | 2.585435 | 6.896E-08 |
| *Tctex1d4* | 2.586947 | 1.034E-12 |
| *Nanos3* | 2.588234 | 1.150E-04 |
| *Fam229a* | 2.589439 | 2.197E-05 |
| *Ndufb10* | 2.593324 | 3.558E-12 |
| *Commd9* | 2.607092 | 5.838E-13 |
| *Znhit2* | 2.609484 | 3.561E-15 |
| *Cops9* | 2.609713 | 2.314E-10 |
| *2300009A05Rik* | 2.614004 | 1.290E-10 |
| *Cfc1* | 2.615553 | 7.399E-06 |
| *Bola2* | 2.615785 | 1.777E-13 |
| *Pfn1* | 2.615799 | 2.703E-11 |
| *Itpka* | 2.616529 | 1.552E-08 |
| *Wfdc15b* | 2.617768 | 2.967E-12 |
| *Atp6v1f* | 2.619571 | 3.349E-12 |
| *Ghrl* | 2.621832 | 2.836E-05 |
| *Gm11273* | 2.62303 | 8.935E-05 |
| *Pet100* | 2.625614 | 3.718E-11 |
| *Acp5* | 2.62823 | 9.676E-14 |
| *1110065P20Rik* | 2.629201 | 2.342E-14 |
| *Dnph1* | 2.630109 | 6.333E-07 |
| *Actb* | 2.633362 | 1.667E-10 |
| *Psma7* | 2.635388 | 1.196E-11 |
| *Coq7* | 2.645576 | 1.897E-13 |
| *Gm49322* | 2.647967 | 1.426E-13 |
| *Ubl5* | 2.649598 | 1.050E-07 |
| *Ptger3* | 2.652996 | 1.114E-06 |
| *Ramp1* | 2.657578 | 2.219E-12 |
| *Ndufa3* | 2.663101 | 1.452E-12 |
| *Atp5j* | 2.663641 | 1.999E-12 |
| *C1qc* | 2.665754 | 7.072E-07 |
| *Gpr20* | 2.66819 | 3.514E-05 |
| *Gngt2* | 2.669839 | 2.684E-10 |
| *Plaur* | 2.670178 | 2.077E-11 |
| *Dlk1* | 2.676604 | 7.102E-04 |
| *Nudt17* | 2.677101 | 3.811E-09 |
| *Ly6c2* | 2.678229 | 7.787E-05 |
| *Lsm4* | 2.679219 | 9.775E-14 |
| *Crygd* | 2.679339 | 2.790E-04 |
| *Cmc2* | 2.681012 | 7.882E-12 |
| *Calm1* | 2.682668 | 1.111E-12 |
| *Prtn3* | 2.686679 | 1.712E-05 |
| *Trappc2l* | 2.686937 | 5.976E-14 |
| *Klk1b27* | 2.688837 | 2.247E-04 |
| *Rnaset2a* | 2.689192 | 1.417E-10 |
| *Krt10* | 2.692078 | 2.497E-10 |
| *Eef1akmt4* | 2.69252 | 2.585E-08 |
| *Saysd1* | 2.697573 | 1.227E-15 |
| *1500015O10Rik* | 2.709262 | 1.868E-11 |
| *mt-Co3* | 2.713337 | 1.552E-09 |
| *Atp6v0c* | 2.713603 | 4.704E-10 |
| *Sf3b5* | 2.721896 | 6.391E-13 |
| *B2m* | 2.724477 | 1.356E-05 |
| *Tmem205* | 2.724554 | 3.282E-10 |
| *B9d2* | 2.732646 | 4.083E-14 |
| *Psmg4* | 2.733878 | 1.189E-15 |
| *Bmyc* | 2.736945 | 7.276E-15 |
| *Gpx4* | 2.741256 | 3.700E-12 |
| *Ier3ip1* | 2.741518 | 6.160E-14 |
| *Sssca1* | 2.742926 | 3.191E-09 |
| *Wdr27* | 2.746037 | 9.087E-06 |
| *Spaca9* | 2.746465 | 9.969E-14 |
| *H2-DMb1* | 2.75188 | 2.941E-08 |
| *Nrn1l* | 2.760266 | 1.645E-05 |
| *Asb5* | 2.765481 | 4.975E-05 |
| *Rbp4* | 2.76625 | 2.664E-14 |
| *Chac1* | 2.772778 | 3.111E-07 |
| *Tmed1* | 2.780008 | 9.422E-16 |
| *Rgcc* | 2.780598 | 1.420E-09 |
| *Scgb1c1* | 2.781562 | 1.728E-08 |
| *Gadd45gip1* | 2.784765 | 5.363E-13 |
| *Ltb* | 2.785738 | 1.685E-04 |
| *Ndufa12* | 2.789352 | 1.481E-11 |
| *Smim6* | 2.790329 | 6.655E-14 |
| *Rex1bd* | 2.794783 | 2.427E-14 |
| *2410015M20Rik* | 2.800699 | 8.819E-13 |
| *Hist1h2bc* | 2.80493 | 7.317E-11 |
| *Ly6d* | 2.809684 | 7.898E-08 |
| *Hdhd3* | 2.810392 | 1.001E-10 |
| *Park7* | 2.810509 | 6.563E-13 |
| *Tpsb2* | 2.813175 | 1.818E-07 |
| *Gm5148* | 2.819274 | 2.733E-04 |
| *Ssr4* | 2.825059 | 5.830E-11 |
| *Pigyl* | 2.826705 | 1.465E-13 |
| *Etfb* | 2.827168 | 7.087E-13 |
| *Mrpl14* | 2.827989 | 5.554E-16 |
| *Elob* | 2.832687 | 2.432E-12 |
| *Rnf186* | 2.837864 | 4.113E-09 |
| *Upk1b* | 2.838797 | 4.338E-12 |
| *Cxcr6* | 2.839817 | 1.641E-04 |
| *Mrpl12* | 2.840595 | 4.035E-13 |
| *Smim20* | 2.842482 | 9.570E-14 |
| *H2-DMb2* | 2.843717 | 2.144E-05 |
| *Tuba1c* | 2.844309 | 1.057E-11 |
| *Lgals2* | 2.846052 | 9.867E-13 |
| *Coprs* | 2.851613 | 2.239E-12 |
| *Ccl9* | 2.855095 | 4.697E-05 |
| *Pilra* | 2.855749 | 9.326E-06 |
| *Scand1* | 2.866375 | 2.427E-14 |
| *Mrpl28* | 2.867538 | 1.570E-12 |
| *Smim4* | 2.872879 | 2.323E-11 |
| *Lgals3* | 2.876865 | 4.015E-14 |
| *Rpp25* | 2.879381 | 9.354E-16 |
| *Polr2f* | 2.881982 | 4.464E-14 |
| *Pnoc* | 2.882355 | 2.507E-04 |
| *Ttc36* | 2.883202 | 1.823E-04 |
| *Svs5* | 2.883928 | 2.814E-04 |
| *Fkbp1b* | 2.886656 | 3.097E-06 |
| *Dad1* | 2.890884 | 2.211E-11 |
| *Abhd17a* | 2.892921 | 2.758E-13 |
| *Rps27l* | 2.895515 | 2.249E-11 |
| *Ninj1* | 2.895649 | 1.122E-13 |
| *Eci1* | 2.898832 | 4.517E-13 |
| *H2-Q2* | 2.900446 | 1.261E-06 |
| *Sac3d1* | 2.901115 | 2.948E-14 |
| *Smim5* | 2.901816 | 6.582E-14 |
| *Prdx5* | 2.902205 | 1.933E-11 |
| *Smim1* | 2.902247 | 7.738E-09 |
| *Tmem212* | 2.905711 | 2.611E-12 |
| *Grcc10* | 2.907333 | 9.578E-13 |
| *Tm2d3* | 2.910631 | 1.509E-12 |
| *Coa3* | 2.913148 | 5.643E-13 |
| *Ecm1* | 2.916654 | 3.856E-12 |
| *Mcub* | 2.917896 | 1.698E-16 |
| *Arhgdib* | 2.922906 | 3.674E-09 |
| *Nat8f1* | 2.929231 | 1.947E-07 |
| *Bloc1s1* | 2.946754 | 1.513E-13 |
| *Cngb1* | 2.950449 | 1.158E-07 |
| *Pgp* | 2.958429 | 4.356E-10 |
| *Snrnp25* | 2.959631 | 5.773E-16 |
| *Pilrb1* | 2.976773 | 8.248E-06 |
| *Chil1* | 2.979688 | 1.752E-06 |
| *Ssbp4* | 2.981904 | 8.702E-16 |
| *Emp3* | 2.985788 | 1.192E-14 |
| *Crygn* | 2.991139 | 1.163E-07 |
| *Ccl21a* | 3.004486 | 4.156E-12 |
| *Dctpp1* | 3.006839 | 4.593E-16 |
| *Fam221b* | 3.008243 | 2.663E-12 |
| *D630036H23Rik* | 3.008608 | 2.545E-07 |
| *Cope* | 3.016598 | 2.791E-12 |
| *Creld2* | 3.018242 | 1.518E-09 |
| *Vamp8* | 3.024272 | 1.967E-11 |
| *Usmg5* | 3.032918 | 2.830E-10 |
| *Gm10320* | 3.033794 | 1.034E-05 |
| *Ndufs5* | 3.035089 | 6.193E-14 |
| *Pomc* | 3.03718 | 4.378E-06 |
| *Psmb6* | 3.037384 | 6.497E-14 |
| *Exosc4* | 3.037941 | 7.745E-15 |
| *Ndufa1* | 3.03946 | 2.705E-13 |
| *Tmem88* | 3.044126 | 1.200E-11 |
| *Cd79a* | 3.047637 | 5.896E-07 |
| *Pop5* | 3.048836 | 3.180E-17 |
| *Dgcr6* | 3.049404 | 5.310E-15 |
| *Naa38* | 3.051202 | 4.342E-14 |
| *Churc1* | 3.052335 | 5.097E-11 |
| *Cox8a* | 3.055425 | 6.677E-12 |
| *Glrx5* | 3.059174 | 1.828E-15 |
| *Arg1* | 3.061331 | 4.294E-08 |
| *Cd63* | 3.062242 | 2.659E-13 |
| *Cldn9* | 3.063528 | 1.205E-06 |
| *Lsm7* | 3.065082 | 6.941E-12 |
| *Mif* | 3.066907 | 1.228E-10 |
| *Trem2* | 3.073955 | 5.708E-11 |
| *Gm14393* | 3.074698 | 8.376E-05 |
| *Dnajc19* | 3.077972 | 4.780E-11 |
| *Oaz1* | 3.081794 | 2.613E-13 |
| *Agrp* | 3.09774 | 1.987E-09 |
| *Mcemp1* | 3.107169 | 5.027E-06 |
| *Avpi1* | 3.107869 | 2.466E-15 |
| *Rarres2* | 3.110642 | 1.780E-09 |
| *Hint2* | 3.115187 | 4.357E-15 |
| *Tm4sf5* | 3.118744 | 8.321E-17 |
| *Gm28040* | 3.121058 | 1.947E-09 |
| *Hist3h2ba* | 3.127754 | 1.438E-05 |
| *Sdf2l1* | 3.129004 | 5.907E-09 |
| *Tat* | 3.146246 | 2.591E-07 |
| *Psmb8* | 3.147936 | 7.469E-10 |
| *Ifi27l2a* | 3.16943 | 6.484E-12 |
| *Rab42* | 3.174873 | 1.343E-07 |
| *Atp5d* | 3.180267 | 9.164E-14 |
| *Hint1* | 3.185358 | 1.133E-14 |
| *Cytl1* | 3.191442 | 1.469E-06 |
| *C1qb* | 3.192879 | 6.773E-08 |
| *Ifitm10* | 3.194934 | 1.204E-05 |
| *1700023F06Rik* | 3.197022 | 1.881E-06 |
| *Olfr1033* | 3.199164 | 4.178E-04 |
| *Enho* | 3.203549 | 7.133E-18 |
| *Atox1* | 3.206574 | 3.371E-15 |
| *Cox4i1* | 3.207216 | 6.605E-15 |
| *Fxn* | 3.224763 | 7.006E-17 |
| *Crym* | 3.228602 | 8.060E-13 |
| *Elof1* | 3.229162 | 5.508E-15 |
| *Rbx1* | 3.232252 | 1.202E-11 |
| *Pin1* | 3.238805 | 1.157E-14 |
| *Atpif1* | 3.240258 | 7.276E-15 |
| *Cd3d* | 3.242073 | 5.663E-05 |
| *Phlda2* | 3.249858 | 2.224E-06 |
| *Fam151a* | 3.251433 | 7.769E-10 |
| *Alkbh7* | 3.253286 | 9.622E-15 |
| *Ccl8* | 3.260834 | 1.940E-07 |
| *Galk1* | 3.262103 | 5.946E-16 |
| *Dcxr* | 3.269063 | 7.504E-13 |
| *Rnasek* | 3.28195 | 1.151E-14 |
| *Tyrobp* | 3.286334 | 1.459E-08 |
| *Sec61g* | 3.288234 | 6.711E-12 |
| *Exosc5* | 3.291145 | 9.393E-14 |
| *Chchd2* | 3.292698 | 1.512E-13 |
| *Cldn3* | 3.292862 | 4.440E-14 |
| *Ndufa5* | 3.294991 | 9.155E-16 |
| *Tmem53* | 3.297853 | 3.739E-18 |
| *Padi4* | 3.311569 | 3.648E-05 |
| *Tmem202* | 3.319027 | 1.957E-08 |
| *Cystm1* | 3.323455 | 1.300E-14 |
| *Rnaset2b* | 3.329034 | 2.430E-13 |
| *Map1lc3a* | 3.336349 | 2.955E-16 |
| *Depp1* | 3.337624 | 2.142E-07 |
| *Chst4* | 3.349575 | 1.057E-15 |
| *Ndufs6* | 3.352948 | 5.158E-16 |
| *Mrps12* | 3.362227 | 1.601E-15 |
| *Timd4* | 3.36979 | 2.487E-06 |
| *Txnl4a* | 3.372442 | 2.779E-12 |
| *Stmn2* | 3.378577 | 3.705E-17 |
| *Ccl6* | 3.383409 | 3.806E-05 |
| *C2cd4b* | 3.383615 | 1.840E-07 |
| *Adig* | 3.392121 | 1.228E-18 |
| *S100a6* | 3.395828 | 8.661E-16 |
| *Gstp1* | 3.398866 | 2.255E-16 |
| *Tmem119* | 3.40352 | 1.312E-14 |
| *B4gat1* | 3.404928 | 2.769E-15 |
| *Fcer1g* | 3.41108 | 1.745E-10 |
| *Psmb5* | 3.414629 | 2.251E-15 |
| *Gstm3* | 3.416501 | 2.391E-07 |
| *Alad* | 3.417427 | 1.151E-14 |
| *Cfp* | 3.424523 | 8.132E-09 |
| *Ifitm1* | 3.425506 | 1.578E-13 |
| *Urah* | 3.432963 | 1.200E-16 |
| *Yif1b* | 3.437884 | 2.109E-17 |
| *Cd2* | 3.44856 | 4.579E-06 |
| *1110017D15Rik* | 3.453375 | 6.150E-16 |
| *H2afx* | 3.454189 | 3.428E-16 |
| *Ms4a4a* | 3.468176 | 3.465E-05 |
| *0610012G03Rik* | 3.477189 | 4.569E-16 |
| *Cox5a* | 3.488901 | 1.151E-14 |
| *Ndufa7* | 3.494243 | 5.158E-16 |
| *Fabp6* | 3.495744 | 2.675E-06 |
| *Hist1h4c* | 3.511617 | 1.757E-06 |
| *Rabac1* | 3.516738 | 2.357E-15 |
| *Fabp4* | 3.522664 | 1.549E-04 |
| *Syngr1* | 3.528195 | 1.244E-10 |
| *Rarres1* | 3.538777 | 6.420E-08 |
| *Car3* | 3.542451 | 4.224E-04 |
| *Ndufa13* | 3.546366 | 9.565E-16 |
| *Tomm6* | 3.546533 | 1.562E-18 |
| *S100a1* | 3.547019 | 1.790E-16 |
| *Smdt1* | 3.5574 | 1.856E-16 |
| *Ndufb7* | 3.567841 | 3.618E-16 |
| *Mettl7b* | 3.570081 | 2.565E-04 |
| *Cd3g* | 3.575253 | 8.141E-05 |
| *Ndufa11* | 3.582904 | 4.511E-16 |
| *Cdk2ap2* | 3.58359 | 6.826E-16 |
| *Tmem223* | 3.602372 | 3.624E-18 |
| *Has1* | 3.610272 | 8.195E-08 |
| *Crip2* | 3.621292 | 1.606E-14 |
| *Bola1* | 3.630994 | 3.739E-18 |
| *Pla2g4f* | 3.633739 | 1.885E-13 |
| *Pcbd2* | 3.645454 | 1.636E-17 |
| *Tppp3* | 3.665774 | 9.520E-17 |
| *Nkain4* | 3.666362 | 1.921E-18 |
| *Sys1* | 3.667001 | 2.176E-13 |
| *Ndufb9* | 3.68439 | 2.116E-17 |
| *Mrps24* | 3.690245 | 1.319E-19 |
| *Mrpl57* | 3.69518 | 2.875E-19 |
| *Ccl19* | 3.73628 | 2.830E-06 |
| *Fth1* | 3.758834 | 2.948E-14 |
| *Uqcrq* | 3.761045 | 8.517E-17 |
| *Angptl6* | 3.763757 | 1.218E-12 |
| *Kcnk7* | 3.766132 | 3.117E-13 |
| *Lypd2* | 3.780066 | 3.008E-08 |
| *Cd8b1* | 3.780329 | 3.600E-05 |
| *Ndufs7* | 3.79049 | 1.592E-16 |
| *Gm10131* | 3.809708 | 7.034E-07 |
| *Gnmt* | 3.81635 | 1.571E-07 |
| *Smim22* | 3.848401 | 3.826E-18 |
| *Sec61b* | 3.858679 | 1.457E-18 |
| *Krt14* | 3.862118 | 7.015E-04 |
| *Hspb1* | 3.865741 | 1.354E-18 |
| *Tmem37* | 3.86688 | 2.732E-12 |
| *Cox8b* | 3.873009 | 9.893E-08 |
| *Osr1* | 3.873421 | 5.901E-15 |
| *Fmc1* | 3.877933 | 7.006E-18 |
| *Cox14* | 3.884333 | 1.278E-18 |
| *Tmem256* | 3.884837 | 2.557E-15 |
| *Nnmt* | 3.886053 | 4.068E-16 |
| *Vsig4* | 3.895958 | 5.816E-04 |
| *Myl7* | 3.897983 | 1.419E-08 |
| *Mrpl54* | 3.909906 | 1.609E-18 |
| *Hist1h2bg* | 3.929304 | 6.890E-10 |
| *C1qtnf4* | 3.957926 | 9.772E-21 |
| *Gng13* | 3.971216 | 4.649E-09 |
| *Usp50* | 3.971628 | 8.002E-23 |
| *Uqcr10* | 3.973741 | 1.105E-18 |
| *Rpl10-ps3* | 3.974448 | 5.066E-11 |
| *Sap18b* | 3.994807 | 4.455E-11 |
| *Dpep2* | 4.014533 | 2.199E-07 |
| *Tmem238* | 4.016475 | 6.590E-19 |
| *Ndufb2* | 4.028744 | 1.370E-16 |
| *H2-T23* | 4.044973 | 1.746E-08 |
| *Ccl17* | 4.049967 | 2.883E-04 |
| *Cidec* | 4.065189 | 2.678E-07 |
| *Cd74* | 4.085347 | 2.220E-07 |
| *Ndufb6* | 4.086686 | 6.770E-19 |
| *Trappc6a* | 4.093049 | 8.606E-21 |
| *Fam25c* | 4.096153 | 8.661E-08 |
| *Ifitm2* | 4.102633 | 4.566E-17 |
| *Polr2l* | 4.105583 | 3.256E-19 |
| *Ltc4s* | 4.113518 | 1.825E-12 |
| *Gm10053* | 4.150926 | 8.128E-09 |
| *Igfbp6* | 4.151014 | 3.844E-18 |
| *Atp5k* | 4.156919 | 5.457E-18 |
| *2310039H08Rik* | 4.160621 | 3.739E-18 |
| *Romo1* | 4.179794 | 2.562E-17 |
| *Timm13* | 4.184486 | 6.630E-21 |
| *Cyba* | 4.186165 | 1.885E-13 |
| *Ifitm3* | 4.187896 | 4.856E-16 |
| *Apoe* | 4.198094 | 1.135E-05 |
| *Cebpb* | 4.19975 | 8.966E-19 |
| *Ftl1* | 4.226986 | 1.247E-16 |
| *Smim27* | 4.230412 | 6.299E-27 |
| *Cxcl13* | 4.294283 | 5.370E-09 |
| *Efcab10* | 4.310853 | 1.172E-15 |
| *Clec1b* | 4.317003 | 2.222E-06 |
| *Ubb* | 4.334267 | 2.436E-14 |
| *Ndufa2* | 4.35453 | 8.037E-19 |
| *Ndufaf8* | 4.36394 | 1.654E-21 |
| *Rps27rt* | 4.397801 | 1.175E-14 |
| *Clec4d* | 4.402485 | 3.498E-04 |
| *Ndufc1* | 4.413175 | 2.527E-22 |
| *H2-Ab1* | 4.441142 | 1.203E-08 |
| *Atp5g1* | 4.450985 | 3.241E-15 |
| *Cox6a1* | 4.456267 | 4.693E-20 |
| *H2-K1* | 4.462675 | 7.214E-08 |
| *H2-Q6* | 4.467304 | 2.315E-04 |
| *Gm12166* | 4.475387 | 6.034E-08 |
| *Nkg7* | 4.475403 | 2.876E-06 |
| *Lyz2* | 4.534285 | 2.105E-08 |
| *Tmem89* | 4.568638 | 1.548E-07 |
| *Hist1h4d* | 4.584569 | 2.463E-11 |
| *Mt1* | 4.596634 | 9.409E-20 |
| *Chchd10* | 4.602122 | 1.227E-22 |
| *Lrg1* | 4.61033 | 2.020E-14 |
| *Hist1h2ai* | 4.629186 | 1.658E-07 |
| *F10* | 4.636161 | 1.456E-05 |
| *Hcst* | 4.649535 | 1.449E-08 |
| *Hist1h2af* | 4.669354 | 2.929E-09 |
| *Msmp* | 4.714031 | 1.378E-09 |
| *Gm2000* | 4.820644 | 1.355E-26 |
| *Retn* | 4.867002 | 8.093E-07 |
| *Ccl5* | 4.906049 | 3.452E-06 |
| *Tspo* | 4.918093 | 6.563E-20 |
| *Mrpl53* | 4.995013 | 1.750E-25 |
| *Uqcr11* | 5.080031 | 6.854E-25 |
| *Cd5l* | 5.099993 | 1.425E-07 |
| *Ndufb8* | 5.124344 | 2.341E-22 |
| *Marco* | 5.172559 | 4.254E-05 |
| *Hbb-bs* | 5.182515 | 1.392E-13 |
| *Gm9844* | 5.187541 | 9.678E-14 |
| *Hist1h2ae* | 5.20089 | 8.290E-19 |
| *Saa3* | 5.355846 | 6.691E-07 |
| *Gm1673* | 5.376875 | 1.578E-30 |
| *Crip1* | 5.384363 | 5.832E-26 |
| *Tmem160* | 5.406645 | 1.015E-27 |
| *Gpha2* | 5.419507 | 1.770E-29 |
| *Apoc1* | 5.433335 | 8.567E-13 |
| *Gm6665* | 5.476712 | 2.260E-12 |
| *H2-D1* | 5.480538 | 5.492E-10 |
| *Ubc* | 5.579412 | 8.976E-20 |
| *Hist2h2ab* | 5.59277 | 1.603E-09 |
| *H2-Q7* | 5.599409 | 4.760E-06 |
| *Nrtn* | 5.723889 | 6.082E-29 |
| *Retnla* | 5.754104 | 7.007E-07 |
| *Lyz1* | 5.777658 | 2.712E-08 |
| *Mia* | 5.806654 | 6.989E-16 |
| *Cyp2e1* | 5.860698 | 2.796E-04 |
| *Gchfr* | 5.98622 | 6.590E-19 |
| *Camp* | 5.994271 | 2.828E-14 |
| *Apoc2* | 6.012227 | 3.139E-09 |
| *Slpi* | 6.205328 | 1.131E-23 |
| *H2afj* | 6.260593 | 4.734E-23 |
| *Lbhd2* | 6.276521 | 1.453E-10 |
| *Fcor* | 6.303485 | 2.146E-25 |
| *H2-Q10* | 6.665771 | 9.628E-07 |
| *Hbb-bt* | 6.704706 | 9.801E-16 |
| *Hist2h2ac* | 6.775139 | 4.914E-18 |
| *Fcna* | 6.818576 | 9.108E-10 |
| *Wfdc17* | 6.826961 | 4.548E-10 |
| *Gm8797* | 6.993175 | 2.226E-26 |
| *Hba-a2* | 7.058147 | 8.137E-09 |
| *Hba-a1* | 7.260924 | 1.228E-21 |
| *Gm20441* | 7.321428 | 5.241E-11 |
| *H2-Q1* | 7.562009 | 1.954E-15 |
| *Cfd* | 7.736237 | 6.877E-05 |
| *Wfdc21* | 7.743903 | 6.613E-08 |
| *Eno1b* | 7.912799 | 2.033E-28 |
| *Gm10591* | 7.930569 | 1.008E-10 |
| *Gm11032* | 7.967166 | 9.562E-06 |
| *Ftl1-ps1* | 7.972478 | 5.185E-40 |
| *Gm20390* | 9.123944 | 4.489E-17 |
| *Hist1h2an* | 9.132261 | 5.208E-09 |
| *Hist1h2ao* | 9.634973 | 1.520E-28 |
| *Apoa2* | 9.641362 | 1.404E-06 |
| *Hist1h2ah* | 10.36425 | 7.315E-17 |
| *Hist2h2aa1* | 10.74477 | 4.485E-47 |
| *Gm49450* | 10.90592 | 5.121E-31 |
| *Hist1h2ak* | 11.44872 | 1.318E-25 |
| *Apoc4* | 12.37873 | 1.765E-11 |
| *Hist1h2ag* | 12.55321 | 7.790E-20 |
| *Hist1h2ap* | 12.65978 | 7.249E-47 |
| *S100a8* | 12.80234 | 7.682E-08 |
| *Hist2h2aa2* | 14.46738 | 9.757E-50 |
